# Progressing nanotechnology to improve targeted cancer treatment: overcoming hurdles in its clinical implementation

**DOI:** 10.1186/s12943-023-01865-0

**Published:** 2023-10-09

**Authors:** Mohammad Chehelgerdi, Matin Chehelgerdi, Omer Qutaiba B. Allela, Renzon Daniel Cosme Pecho, Narayanan Jayasankar, Devendra Pratap Rao, Tamilanban Thamaraikani, Manimaran Vasanthan, Patrik Viktor, Natrayan Lakshmaiya, Mohamed J. Saadh, Ayesha Amajd, Mabrouk A. Abo-Zaid, Roxana Yolanda Castillo-Acobo, Ahmed H. Ismail, Ali H. Amin, Reza Akhavan-Sigari

**Affiliations:** 1 Novin Genome (NG) Institute, Research and Development Center for Biotechnology, Shahrekord, Chaharmahal and Bakhtiari Iran; 2 Young Researchers and Elite Club, Shahrekord Branch, Islamic Azad University, Shahrekord, Chaharmahal and Bakhtiari Iran; 3 Department of Pharmacy, Al-Noor University College, Nineveh, Iraq; 4 Department of Biochemistry, Universidad San Ignacio de Loyola (USIL), Lima, Peru; 5 Department of Pharmacology, SRM Institute of Science and Technology, SRM College Of Pharmacy, Chengalpattu District, Kattankulathur, Tamil Nadu 603203 India; 6 Department of Chemistry, Coordination Chemistry Laboratory, Dayanand Anglo-Vedic (PG) College, Kanpur-208001, U.P India; 7 Department of Pharmaceutics, SRM Institute of Science and Technology, SRM College Of Pharmacy, Chengalpattu District, Kattankulathur, Tamil Nadu 603203 India; 8 Keleti Károly Faculty of Business and Management, Óbuda University, Tavaszmező U. 15-17, 1084 Budapest, Hungary; 9 Department of Mechanical Engineering, Saveetha School of Engineering, SIMATS, Chennai, Tamil Nadu India; 10 Faculty of Pharmacy, Middle East University, Amman, 11831 Jordan; 11 Faculty of Organization and Management, Silesian University of Technology, 44-100 Gliwice, Poland; 12 Department of Mechanical Engineering, CEMMPRE, University of Coimbra, Polo II, 3030-788 Coimbra, Portugal; 13 Department of Biology, College of Science, Jazan University, 82817 Jazan, Saudi Arabia; 14 Universidad Nacional de San Agustin de Arequipa, Arequipa, Peru; 15 Deanship of Scientific Research, Umm Al-Qura University, Makkah, 21955 Saudi Arabia; 16 Department of Neurosurgery, University Medical Center, Tuebingen, Germany; 17 Department of Health Care Management and Clinical Research, Collegium Humanum Warsaw Management University Warsaw, Warsaw, Poland

**Keywords:** Nanotechnology, Cancer detection, Cancer treatment, Nanoscale targeting techniques, Protein engineering, Materials science, Nanocarriers, Medicinal purposes, Human trials

## Abstract

The use of nanotechnology has the potential to revolutionize the detection and treatment of cancer. Developments in protein engineering and materials science have led to the emergence of new nanoscale targeting techniques, which offer renewed hope for cancer patients. While several nanocarriers for medicinal purposes have been approved for human trials, only a few have been authorized for clinical use in targeting cancer cells. In this review, we analyze some of the authorized formulations and discuss the challenges of translating findings from the lab to the clinic. This study highlights the various nanocarriers and compounds that can be used for selective tumor targeting and the inherent difficulties in cancer therapy. Nanotechnology provides a promising platform for improving cancer detection and treatment in the future, but further research is needed to overcome the current limitations in clinical translation.

**Graphical Abstract:**

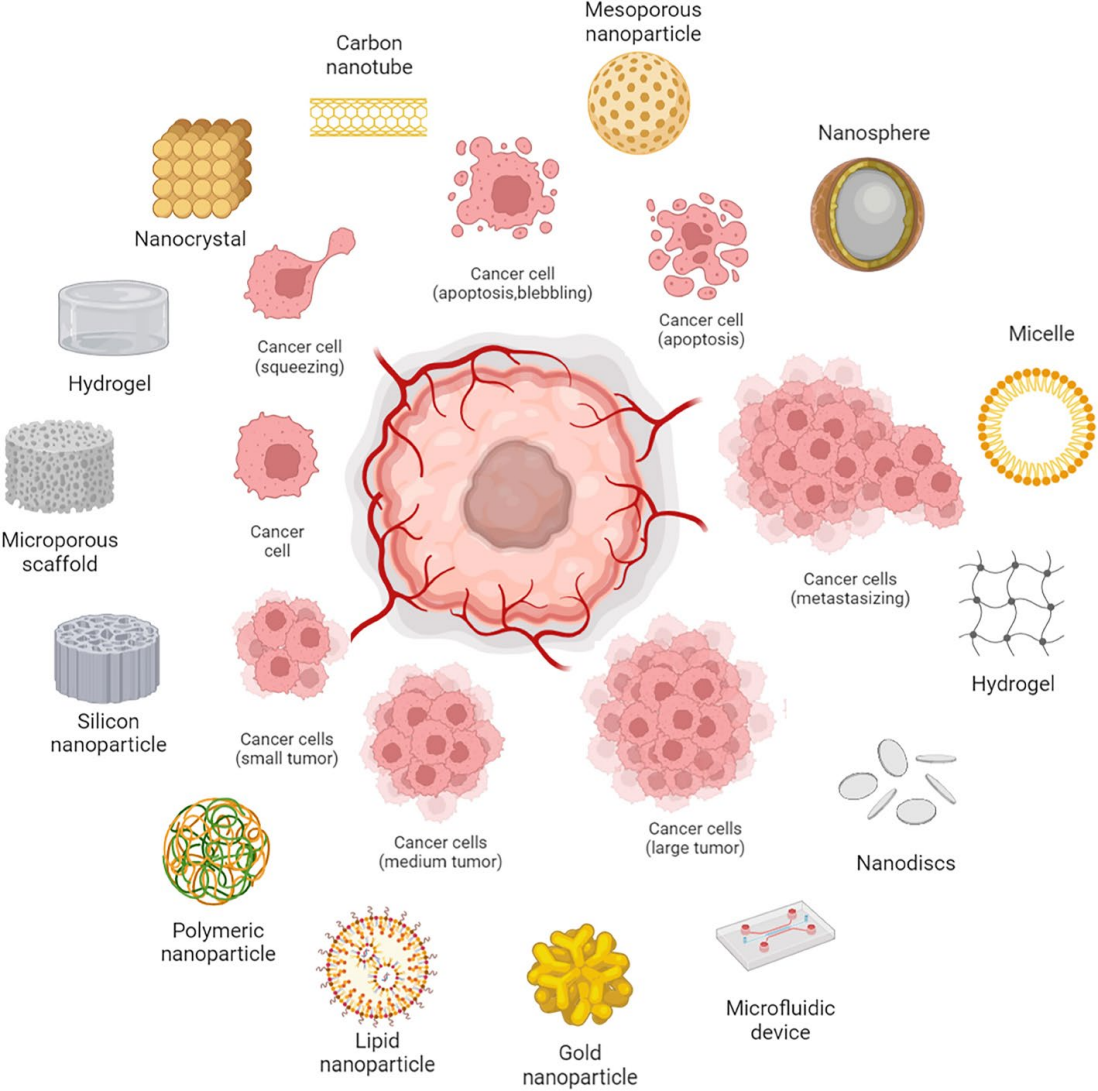

## Introduction

Cancer, an intricate ailment that has long posed formidable therapeutic challenges, demands novel approaches that can surmount the limitations of conventional treatments like chemotherapy and radiation therapy, which often inflict severe side effects and yield unsatisfactory outcomes [1]. In this landscape of medical exigency, nanotechnology has emerged as a promising paradigm for the detection and treatment of cancer. Nanotechnology harnesses the ability to engineer and manipulate materials on the nanoscale, typically within the realm of 1 to 100 nm. The unique physicochemical properties of these diminutive materials confer distinctive interactions with cells and tissues, thereby paving the way for innovative nanoscale targeting techniques that might catalyze transformative shifts in cancer diagnosis and therapy [2]. The current milieu of cancer therapy underscores the compelling need for groundbreaking methodologies, and this paper delves into the potentialities offered by nanotechnology to address this critical necessity [1].

Nanocarriers stand out as a focal point in the convergence of nanotechnology and cancer treatment. These minute carriers, adept at encapsulating therapeutic agents such as drugs or genes, present an array of advantages surpassing the confines of traditional treatments [3]. Key among these advantages are pinpoint accuracy in targeting cancer cells, mitigated harm to healthy cells, and amplified efficacy of therapeutic payloads [4]. The trajectory of nanocarrier-based cancer cell targeting is manifested through two principal avenues: passive targeting and active targeting. Passive targeting capitalizes on the distinctive attributes of tumor cells, such as their permeable blood vessels, to foster accumulation of nanocarriers within the tumor microenvironment [2, 4]. Conversely, active targeting involves surface modifications of nanocarriers with specific targeting ligands that bind to receptors decorating the surfaces of cancer cells, facilitating precise and enhanced cellular engagement [5].

Although diverse nanocarriers have traversed preclinical phases and garnered approvals for human trials, a mere fraction have secured authorization for clinical deployment, particularly those with molecular moieties designed for selective cancer cell interactions [4]. This juncture accentuates the intricacies of transitioning laboratory discoveries to effective clinical interventions, underscoring the imperative for further research to optimize the therapeutic potential of nanocarriers in the context of cancer therapy [6]. The marriage of nanotechnology and cancer treatment holds the promise of optimizing the efficacy of therapeutic agents, curbing collateral damage to healthy cells, and elevating patient prognoses [2]. However, navigating this promising terrain is not devoid of hurdles, encompassing the refinement of cost-effective and efficient nanocarriers, assurance of the safety profile of these carriers in human settings, and surmounting barriers obstructing the translation of laboratory insights to tangible clinical outcomes [6].

The primary scope of our article is to examine the advancements and challenges in utilizing nanotechnology for targeted cancer therapy. We will specifically focus on nanocarriers and compounds that demonstrate potential for selective tumor targeting. The nanocarriers covered will encompass liposomes, nanoparticles, and micelles, among others. As for authorized formulations, we will select those that have undergone clinical trials or have been approved for clinical use in targeting cancer cells. The criteria for selecting these authorized formulations will include their demonstrated efficacy in targeting cancer cells, their safety profile, and their potential for clinical translation. By providing this clarity, our review aims to shed light on the current landscape of nanotechnology-based cancer therapies and the criteria that underlie the selection of promising formulations for clinical application.

## Methods of passive and active targeting

**Fig. 1:**
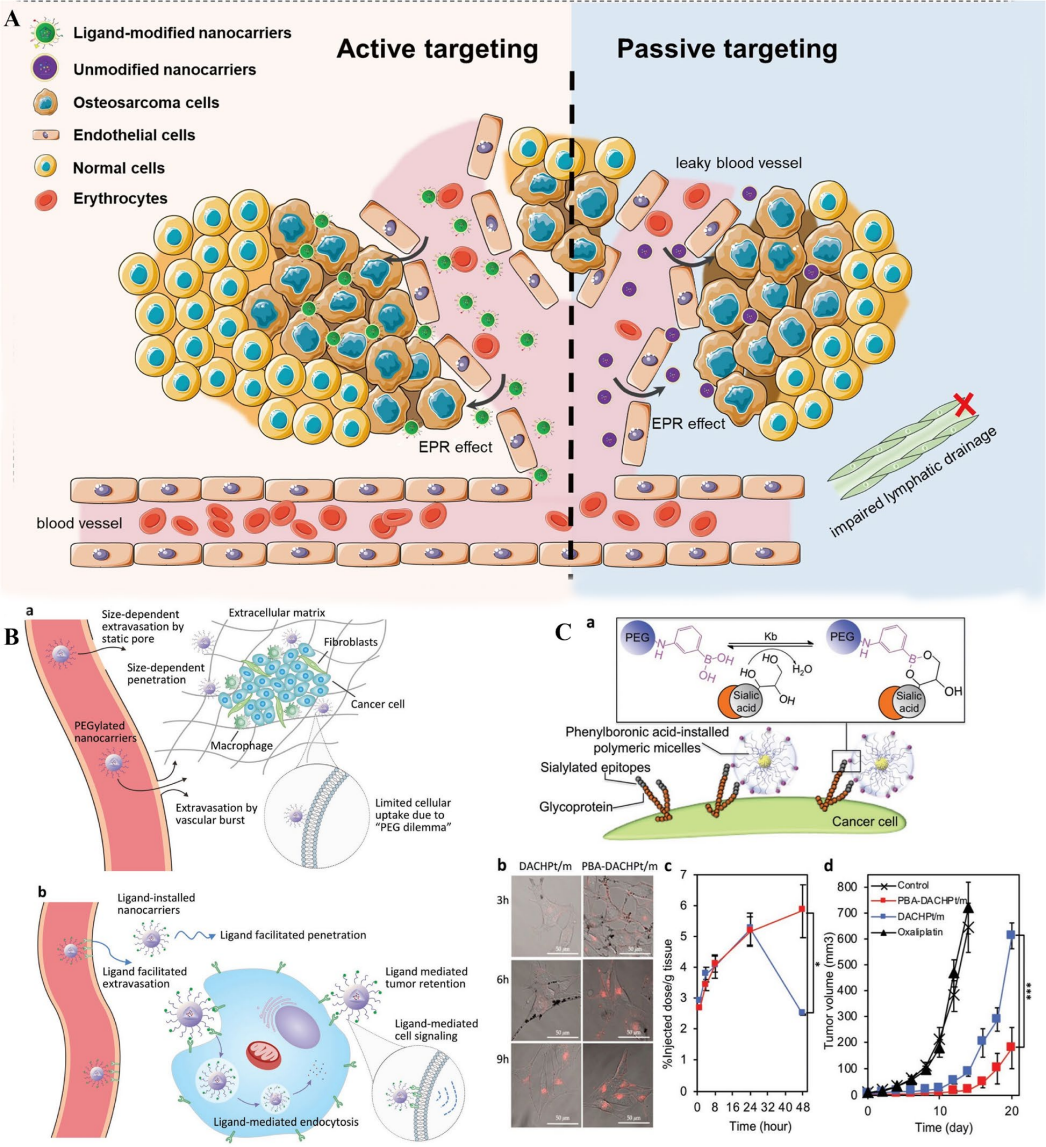
**A** An illustrative diagram depicting the concepts of active and passive targeting in nano-delivery systems for anti-tumor treatment. Passive targeting relies on the enhanced permeability and retention (EPR) effects, where nanocarriers circulate in the bloodstream, exit into the tumor tissue through the leaky tumor blood vessels, and accumulate there. On the other hand, nanocarriers modified with targeting ligands can specifically attach to receptors that are overexpressed on tumor cells, enabling localized drug delivery or internalization via receptor-mediated endocytosis. Reprint from [33] with a permission from Springer Nature. **B** A diagrammatic representation of a targeting ligand-conjugated nanocarrier. Tumor targeting by nanocarriers (**A**) and ligand-installed nanocarriers **B**). Reprint from [34] with a permission from Wiley. **C** The ability of ligand-installed nanocarriers to target cancer cells. **A** illustrates the use of phenylboronic-acid-installed DACHPt-loaded polymeric micelles (PBA-DACHPt/m) for targeting cancer cells that overexpress sialylated epitopes receptors; **B** shows the cellular uptake of micelles with and without PBA ligands by B16F10 cancer cells; **C** displays the tumor accumulation of PBA-DACHPt/m and DACHPt/m; and (**D**) exhibits the tumor suppression effect of PBA-DACHPt/m micelles against subcutaneous B16F10 tumor models. Reprint from [34] with a permission from Wiley

Nanocarriers are being increasingly investigated as a promising approach to cancer treatment, but they face numerous roadblocks on their journey to the targeted site [7]. In a recent study by Baker et al., the potential of smart nanocarriers in the targeted delivery of therapeutic nucleic acids for cancer immunotherapy has been explored. Cancer treatment has seen remarkable progress with the advent of immunotherapy, particularly through the use of antibodies targeting immune checkpoints. However, the field of cancer immunotherapy is evolving, with a growing emphasis on nucleic acid technology, including cancer vaccines, adoptive T-cell therapies, and gene regulation. Yet, these promising approaches face significant challenges related to their effective delivery to target cells, including issues such as in vivo decay, limited uptake by target cells, the need for nuclear penetration, and potential damage to healthy cells. The study highlights the pivotal role of advanced smart nanocarriers, such as lipids, polymers, spherical nucleic acids, and metallic nanoparticles, in overcoming these barriers. These nanocarriers offer a means to efficiently and selectively deliver nucleic acids to the desired cells and tissues, thereby improving the overall efficacy, reducing toxicity, and enhancing stability of cancer therapeutics in the context of immunotherapy. This research underscores the potential of nanotechnology as a promising approach in the ongoing battle against cancer [8]. Table 1 highlights the various nanocarrier types for cancer therapy and their respective properties. Mucosal barriers and non-specific absorption are just a few of the challenges encountered in employing nanocarriers for cancer therapy. To overcome these obstacles, a combination of rational nanocarrier design and a fundamental understanding of tumor biology is needed [6]. Tumors are characterized by a variety of symptoms, but two of the most prevalent ones are leaky blood vessels and poor lymphatic drainage. Nanocarriers can take advantage of these characteristics through the EPR (enhanced permeability and retention) effect, which allows them to escape into tumor tissues via leaky arteries and distribute drugs to the region surrounding the tumor cells [9]. In addition, the size of the nanocarrier is also important, with particles with diameters of 200 nm being found to be more efficient, although experiments utilizing liposomes of varying mean sizes imply that the threshold vesicle size for extravasation into tumors is 400 nm [10]. Although passive targeting methods form the backbone of therapeutic practice, they are not without their flaws. One major issue is the possibility that not all of the tumor's cells may be accessible for treatment [6, 10]. This can be due to the inability of certain pharmaceuticals to disperse well, making it difficult to control the process. The lack of control can lead to multiple-drug resistance (MDR), where chemotherapy treatments fail because the cancer is resistant to the drugs [11]. This is facilitated by the overexpression of transporter proteins on the surface of cancer cells that eliminate drugs from cells [5]. Figure 1 presents a study on the ability of ligand-installed nanocarriers to target cancer cells. Active targeting, where nanocarriers actively adhere to the cells they are targeting following extravasation, is one way to circumvent the limitations of passive targeting (Fig. 1A). Ligands, which are targeted agents, can be attached to the surface of the nanocarrier using various conjugation chemistry methods. Ligand-receptor interactions enable the nanocarrier to identify and attach to its intended cells [9]. The nanocarrier system is designed to target tumors, and the diagram shows two different mechanisms of tumor targeting: (Fig. 1B-A) nanocarriers that are capable of passive targeting, and (Fig. 1B-B) nanocarriers that are equipped with ligands for active targeting. In passive targeting, nanocarriers accumulate in tumors due to their small size and the leakiness of tumor blood vessels. In active targeting, ligands attached to the surface of the nanocarriers bind specifically to receptors on the surface of tumor cells, which results in the accumulation of the nanocarriers in the tumor and increased therapeutic efficacy. This nanocarrier system represents an exciting and promising approach to targeted drug delivery, offering the potential for more effective and less toxic cancer treatments. In (Fig. 1C-A), phenylboronic-acid-installed DACHPt-loaded polymeric micelles (PBA-DACHPt/m) are used to target cancer cells that overexpress sialylated epitopes receptors. The cellular uptake of micelles with and without PBA ligands by B16F10 cancer cells is shown in (Fig. 1C-B). The results demonstrate that PBA-DACHPt/m micelles have a higher uptake rate than those without PBA ligands. In (Fig. 1C-C), the tumor accumulation of PBA-DACHPt/m and DACHPt/m is displayed, and it is found that PBA-DACHPt/m micelles accumulate more in the tumor tissue than DACHPt/m micelles. Finally, in (Fig. 1C-D), the tumor suppression effect of PBA-DACHPt/m micelles against subcutaneous B16F10 tumor models are exhibited, indicating that PBA-DACHPt/m micelles possess superior tumor suppression ability. These findings suggest that the use of ligand-installed nanocarriers could potentially improve cancer therapy by enhancing drug delivery to the tumor site. Receptor-mediated internalization is generally necessary for nanocarriers to transport drugs into the cell. Target cells must have an overabundance of a surface marker compared to nontarget cells for maximum specificity, and targeted efficacy rises in tandem with binding affinity. However, there is evidence that a "binding-site barrier" may prevent nanocarriers from penetrating solid tumors when they have a high binding affinity [9]. While nanocarriers show great promise for cancer therapy, there are still many challenges that need to be addressed. The design of nanocarriers needs to be optimized to ensure better efficacy and safety in humans, and further research is needed to develop more efficient and cost-effective nanocarriers [6, 10]. A better understanding of tumor biology and the development of innovative targeting techniques will also be necessary to overcome the limitations of passive targeting and maximize the potential of nanocarriers for cancer treatment. Improving targeting may need a combination of affinity enhancement and multivalent binding effect enhancement (also known as avidity) [12]. Collective binding during multivalent contact is far stronger than binding during individual interactions [10, 12]. One of the promising ways to achieve multivalent binding is through the use of dendrimers, which are highly branched polymers that allow for the attachment of multiple targeting molecules. For instance, dendrimer nanocarriers conjugated to anywhere from three to fifteen folate molecules have shown a significant increase in binding affinity when bound to immobilized folate-binding proteins, as compared to free folate. Despite these advances, there are still several challenges that need to be addressed in the development of targeted nanocarriers. One major challenge is the issue of heterogeneity, where different regions of a tumor may have varying levels of expression of the target receptor [10, 12]. This can lead to ineffective or non-specific targeting, reducing the efficacy of the nanocarrier. Additionally, the development of drug resistance is a major concern, as cancer cells can quickly adapt and develop resistance to new drugs. The use of nanocarriers for targeted cancer therapy is an exciting area of research that offers significant potential for improving patient outcomes. While there are still many challenges to be overcome, the development of novel nanocarrier designs and improved understanding of tumor biology offer hope for the continued advancement of this promising field. With further research and development, it may be possible to create targeted nanocarriers that are highly effective at delivering drugs to cancer cells, minimizing side effects, and improving the overall efficacy of cancer treatment [9, 12].

**Table 1:** Comparison of nanocarrier types for cancer therapy

**Nanocarrier Type**	**Size Range**	**Surface Charge**	**Drug Payload Capacity**	**Targeting Mechanism**	**Biodegradability**	**Description**	**Novelty**	**Advantages**	**Disadvantages**	**Limitations/Challenges**	**References**
**Liposomes**	50–200 nm	Neutral	Low–High	Passive/Active	Biodegradable	Spherical structures composed of a lipid bilayer enclosing an aqueous core	First-generation nanocarriers for drug delivery, used in clinical practice	Good biocompatibility, low immunogenicity, versatility in drug loading and targeting	Short circulation time, potential drug leakage, lack of tumor specificity	Limited drug payload capacity, challenges in scaling up production, difficulty in achieving controlled drug release in vivo	[13]
**Polymeric nanoparticles**	10–200 nm	Variable	Low–High	Passive/Active	Biodegradable	Solid particles made of synthetic or natural polymers	Wide range of materials and formulations, suitable for various administration routes	High drug loading capacity, stable in circulation, tunable surface properties	Potential toxicity, burst release of drug, batch-to-batch variation, difficulty in achieving targeted drug delivery to tumors	Challenges in achieving controlled release, low targeting efficiency, limited biocompatibility of some materials	[14, 15]
**Dendrimers**	1–10 nm	Variable	Low-Moderate	Passive/Active	Non-biodegradable	Branched, highly branched or spherical molecules with defined size and shape	Highly customizable, multivalent surface chemistry, high drug loading capacity	High biotoxicity, low biodegradability, challenges in scaling up production	Limited blood circulation time, potential renal toxicity, difficulty in achieving targeted drug delivery to tumors	Limited targeting efficiency, challenges in achieving controlled release, potential immunogenicity	[15]
**Gold nanoparticles**	1–100 nm	Neutral	Low-Moderate	Passive/Active	Non-biodegradable	Spherical or rod-shaped particles made of gold	Excellent biocompatibility, high surface plasmon resonance effect, stability in biological fluids	Low drug loading capacity, limited tumor penetration, challenges in scaling up production	Potential toxicity, limited targeting efficiency, difficulty in achieving controlled drug release in vivo	Limited biocompatibility of some surface modifications, potential immunogenicity	[16, 17]
**Carbon nanotubes**	1–100 nm	Negative	Low–High	Passive/Active	Non-biodegradable	Hollow cylindrical structures made of carbon atoms	High aspect ratio, high drug loading capacity, potential for multi-functionalization	High toxicity, limited biocompatibility, challenges in achieving controlled release	Limited blood circulation time, potential clearance by the reticuloendothelial system, difficulty in achieving targeted drug delivery to tumors	Potential immunogenicity, difficulty in scaling up production	[18, 19]
**Iron oxide nanoparticles**	5–100 nm	Negative	Low-Moderate	Passive/Active	Biodegradable	Magnetic particles made of iron oxide	High targeting specificity, potential for MRI imaging and magnetic hyperthermia	Low drug loading capacity, limited blood circulation time, challenges in achieving controlled release	Potential toxicity, limited tumor penetration, difficulty in scaling up production	Potential immunogenicity, low biocompatibility of some surface modifications	[20]
**Quantum dots**	1–10 nm	Negative	Low-Moderate	Passive/Active	Non-biodegradable	Semiconductor nanocrystals	High brightness, tunable emission spectrum, potential for multiplexed imaging	High toxicity, potential for heavy metal leaching, challenges in achieving targeted drug delivery	Limited blood circulation time, potential clearance by the reticuloendothelial system, difficulty in scaling up production	Potential immunogenicity, limited tumor specificity	[21]
**Silica nanoparticles**	10–500 nm	Negative	Low–High	Passive/Active	Biodegradable	Solid particles made of silica	High drug loading capacity, good stability, tunable surface properties	Potential toxicity, limited blood circulation time, difficulty in achieving targeted drug delivery to tumors	Limited biocompatibility, challenges in achieving controlled release	Potential immunogenicity, limited tumor specificity	[22]
**Mesoporous silica nanoparticles**	20–200 nm	Negative	Low–High	Passive/Active	Biodegradable	Porous particles made of silica	High surface area, high drug loading capacity, tunable pore size and surface chemistry	Potential toxicity, limited blood circulation time, difficulty in achieving controlled drug release in vivo	Limited biocompatibility, challenges in achieving targeted drug delivery to tumors	Potential immunogenicity, limited tumor specificity	[23]
**Lipid-nucleic acid nanoparticles**	50–200 nm	Neutral	Low-Moderate	Active	Biodegradable	Nanoparticles made of lipids and nucleic acids	Suitable for nucleic acid delivery, good biocompatibility, low toxicity	Limited drug loading capacity, potential instability, challenges in achieving efficient delivery	Potential immunogenicity, limited blood circulation time	Limited targeting efficiency, difficulty in scaling up production	[24, 25]
**Protein nanoparticles**	2–200 nm	Variable	Low-Moderate	Passive/Active	Biodegradable	Nanoparticles made of proteins or peptides	Good biocompatibility, low toxicity, potential for targeted delivery	Limited drug loading capacity, challenges in achieving efficient drug release in vivo	Potential immunogenicity, limited stability, limited blood circulation time	Limited targeting efficiency, difficulty in scaling up production	[26, 27]
**Inorganic–organic hybrid nanoparticles**	10–200 nm	Variable	Low–High	Passive/Active	Biodegradable	Nanoparticles made of a combination of inorganic and organic components	Highly customizable, multifunctional, high drug loading capacity	Potential toxicity, limited blood circulation time, challenges in achieving controlled drug release in vivo	Limited biocompatibility, difficulty in achieving efficient targeting	Potential immunogenicity, limited tumor specificity	[28]
**Metal–organic frameworks**	10–500 nm	Variable	Low–High	Passive/Active	Biodegradable	Porous crystalline materials made of metal ions and organic ligands	Highly customizable, tunable pore size and surface chemistry, high drug loading capacity	Potential toxicity, limited blood circulation time, challenges in achieving efficient targeting	Limited biocompatibility, potential for drug leakage, limited stability	Potential immunogenicity, limited tumor specificity	[29]
**Exosomes**	30–150 nm	Negative	Low-Moderate	Active	Biodegradable	Small extracellular vesicles derived from cells	High biocompatibility, potential for targeted delivery, natural carriers of biological cargoes	Limited drug loading capacity, challenges in achieving efficient targeting, potential for premature drug release	Limited blood circulation time, difficulty in scaling up production	Limited targeting efficiency, potential for immune system recognition	[30]
**Bacterial nanoparticles**	10–300 nm	Negative	Low-Moderate	Active	Biodegradable	Nanoparticles produced by bacteria	High biocompatibility, potential for targeted delivery, easy to produce	Limited drug loading capacity, potential for immunogenicity, limited control over drug release	Limited blood circulation time, difficulty in achieving efficient targeting	Limited targeting efficiency, potential for clearance by the immune system	[31]
**Polymeric micelles**	10–100 nm	Variable	Low-Moderate	Passive/Active	Biodegradable	Spherical particles made of block copolymers	High drug loading capacity, good stability, easy to produce	Limited blood circulation time, challenges in achieving efficient targeting, potential for premature drug release	Limited biocompatibility, difficulty in achieving controlled release	Potential immunogenicity, limited tumor specificity	[32]

## Distinct categories of targeting agents

**Fig. 2:**
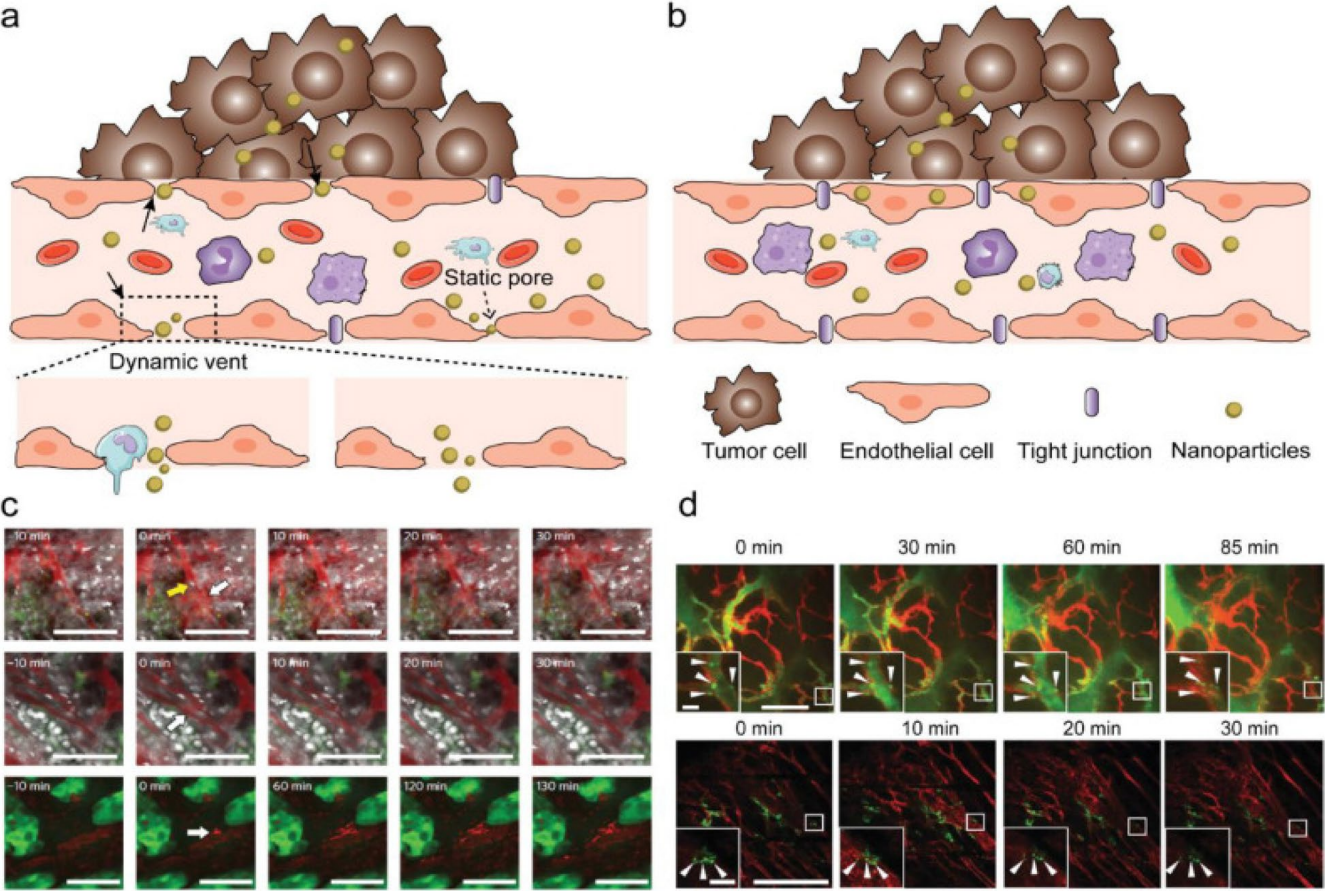
New insights on the transport of nanoparticles (NPs) through endothelial cells using intravital microscopy (IVM). Panel (**A**) shows NP transport through gaps between adjacent endothelial cells in dynamic vascular bursts, while panel (**B**) shows NP transport across the endothelial cell layer via transcytosis. Panel (**C**) presents representative images of eruptions occurring near and without leukocyte cells, respectively, using 70 nm Doxil particles and a BxPC3-GFP dorsal skinfold model. Panel (**D**) demonstrates colocalization of NPs with endothelial cells to form hotspots along the vessel lining in MMTVPyMT and 4T1 tumor models using 50 nm AuNPs conjugated with Alexa Fluor 647. The scale bars for all panels are provided, and insets are included where appropriate. Reprint from [47] with a permission from Elsevier

Targeting agents can be put into three broad categories: proteins (mostly antibodies and their fragments), nucleic acids (aptamers), and other receptor ligands (peptides, vitamins, and carbohydrates). In 1981, Milstein was the first to publicly discuss using a monoclonal antibody to kill cancer cells. The clinical viability of antibody-based tissue targeting has been shown over the last two decades, and the FDA has licensed 17 different mAbs. In 1997, FDA approval of the monoclonal antibody rituximab (trade name: Rituxan) for the treatment of patients with non-Hodgkin's lymphoma was granted. Afterwards, a year later, the anti-HER2 monoclonal antibody trastuzumab (Herceptin) was approved for use in the treatment of breast cancer [35]. In a recent groundbreaking study by Ferguson et al., a significant advancement in the field of nanomedicine has been achieved, addressing the persistent challenge of achieving precise drug delivery to specific target cells and organs. The research introduces a novel approach called Dual Affinity to RBCs and Target Cells (DART), which utilizes nanocarriers conjugated with two affinity ligands. One ligand binds to red blood cells (RBCs), while the other binds to target cells, specifically pulmonary endothelial cells in this study. This innovative strategy allows DART nanocarriers to initially bind to RBCs and subsequently transfer to the endothelial cells of the target organ, in this case, the lungs. Remarkably, within minutes of intravascular injection in mice, DART nanocarriers achieve an accumulation of nearly 70% of the injected dose in the target organ, a remarkable improvement compared to previous technologies. Humanized DART nanocarriers tested in ex vivo perfused human lungs replicate this success. Furthermore, DART demonstrates a six-fold enhancement in the selectivity of drug delivery to target endothelial cells over local phagocytes within the target organ. This groundbreaking advancement in both organ- and cell-type targeting holds tremendous promise for the localized delivery of drugs, particularly in the context of cancer treatment, where precise targeting is of paramount importance [36]. In 2004, the anti-VEGF monoclonal antibody bevacizumab (Avastin) was approved for use in the treatment of colorectal cancer. It was the first time a disease was treated using an angiogenesis inhibitor. It is estimated that over 200 distinct antibody-or antibody-fragment-based delivery techniques are now being evaluated in preclinical and clinical settings. Antibody engineering has advanced to the point where hybrid antibodies may be synthesized; these include chimeric mAbs, humanized mAbs (which have a higher human contribution), and antibody fragments [37]. For the sake of targeting, antibodies may be used either in their whole, unaltered form or as subunits. However, the availability of two binding sites (within a single antibody) leads to a larger binding avidity, making the use of full monoclonal antibodies preferable. Moreover, when immune cells bind to the Fc region of the antibody, a signaling cascade is initiated that ultimately kills cancer cells. This particular component is featured in the antibody. The Fc domain of an unmodified mAb, on the other hand, may bind to Fc receptors on normal cells like macrophages [35, 37]. This may boost the nanocarrier's uptake by the liver and spleen, as well as its immunogenicity (the ability to induce an immune response). Another advantage of employing full or complete antibodies is that they may be kept stable for long periods of time. Due to their decreased non-specific binding, modified antibody fragments such as antigen-binding fragments (Fab), dimers of antigen-binding fragments (F(ab)2), single-chain fragment variables (scFv), and others are safer for systemic injection. Phage display libraries that use a high throughput method can be used to rapidly identify antibodies or their fragments that bind to and internalize cancer cells. Despite the fact that antibody fragments such as antigen-binding fragments (Fab) and dimers of antigen-binding fragments (F(ab) can be used, this is not the case [38, 39]. With this method, several different antibodies may be generated, each with the ability to bind to the same set of target cells but with different epitopes (a part of a macromolecule that is recognized by antibodies; one receptor may have several epitopes that will be recognized by multiple antibodies). One example is the development of scFv antibodies with improved binding and internalization properties for prostate cancer cells using a selection process. It is possible to increase the efficiency of an antibody by directly conjugating a medicinal molecule to it for targeted distribution. Calicheamicin, a chemotherapeutic medication, was the first formulation approved for use in the clinic that specifically targets cancer cells [38, 39]. The combination of this drug (marketed under the trade name Mylotarg) with an anti-CD33 antibody makes cancer cells a clear target. A few examples include Zevalin and Bexxar, which use anti-CD20 antibodies to target cancer cells with radioisotopes. While the efficacy of these therapies has been established, certain studies have shown that they may have deadly adverse effects [40]. Non-specific binding between the agent of interest and non-target moieties on the cell surface is likely to be to blame for these effects. When the targeting agent is produced by healthy cells rather than cancerous ones, it may interact with the target. An immunoconjugate called BR96-doxorubicin, which consists of an antibody that targets and binds to the Lewis-Y antigen (expressed on 75% of all breast tumors), showed a strong anti-tumor effect in animal tumor models. To do this, doxorubicin was conjugated to an antibody that recognizes and binds to the Lewis-Y antigen. Compared to doxorubicin alone, BR96-doxorubicin showed promising results in these animal models with much reduced toxicity [38, 39]. However, canines had symptoms consistent with acute enteropathy. Conjugate binding to Lewis-Y-related antigens generated by untargeted gastrointestinal epithelial cells seems to be to blame for this phenomenon. The Phase II human clinical research using BR96-doxorubicin immunoconjugates showed modest anti-tumor activity and caused serious gastrointestinal harm, hence the trial was stopped. Selecting appropriate targets using genomics and proteomics technologies is an essential area of research. However, to date, no targets have been uncovered that are therapeutically useful. There seems to be more hope in the development of new technologies that may enhance selectivity and targeting efficacy while still making use of current targets [38, 39]. It is possible to create a new protein with the desired properties by fusing two or more genes, as in the case of fusion proteins. Molecular biology techniques may be used to successfully create protein-based ligand mimetics that mimic a receptor's structure. These mimetics may be developed in the same way that antibodies can be engineered to attach more strongly to their targets [41]. Dimerization of proteins or peptides may boost ligand affinity via a process known as divalency, which includes the simultaneous binding of a protein or peptide to an antibody's two Fc domains. Divalency is a means through which ligand affinity may be enhanced. For instance, increasing tumor localization in a mouse tumor model was seen, for instance, when a low-affinity scFv (also known as a diabody) was dimerized. In addition, it is possible to improve binding affinity and selectivity to cell surface receptors by designing proteins that identify a specific conformation of a target receptor [42]. The affinity for the target receptor, integrin LFA-1, was boosted 10,000-fold in a recent in vivo investigation employing a fusion protein comprised of a scFv antibody fragment to target and deliver small interfering RNA (siRNA) to lymphocytes. Using a fusion protein to specifically target and deliver siRNA to lymphocytes yielded the desired effect. Integrin LFA-1 is generally expressed on peripheral leukocytes in its low-affinity, non-adhesive form (white blood cells that have not been activated by cancer cells or pathogens that have entered the body) [43]. On the other hand, when the immune system is stimulated, this low-affinity, sticky version of integrin LFA-1 undergoes conformational modifications and becomes the high-affinity, adhesive form. Therefore, drugs may be delivered selectively to activated and sticky leukocytes by targeting the high-affinity form of LFA-1 [43]. In order to target certain conformations, it is feasible to create novel classes of targeting chemicals. Affibodies are one example; they are small protein domains that may be tailored to bind selectively to a wide range of target proteins in a manner that is sensitive to conformational changes. Multivalent effects include the use of several small proteins that act like antibodies to bind selectively to various receptors. The proteins that have this structure are called avimers [9]. Cancer markers include the protein carcinoembryonic antigen (CEA). In order to bind to CEA, scientists have employed nanobodies, which are heavy-chain antibodies that have been created to be one tenth of the size of an intact antibody with a missing light chain. It is not only antibodies that have benefited from high-throughput methods; aptamers and other targeting molecules have also been designed using rational approaches [9]. In vitro-selected aptamers are short oligonucleotides (oligonucleotides with just one strand of DNA or RNA) (1014–1015). Aptamers are selected for their broad specificity in terms of the targets they may bind to, which can vary from intracellular proteins and transmembrane proteins to soluble proteins and carbohydrates to small-molecule drugs [11]. Several aptamers that target specific cancer cell receptors have also been developed. Therefore, aptamer-conjugated nanoparticles may be an effective method of therapy [44]. So, for instance, nanoparticles encapsulating docetaxel (Dtxl) have been administered in vivo with high selectivity and efficacy. This was made feasible by adding an aptamer to the nanoparticles, which specifically targets the antigen on the surface of prostate cancer cells [15]. Figure 2-A shows NP transport through gaps between adjacent endothelial cells in dynamic vascular bursts, while Fig. 2-B demonstrates NP transport across the endothelial cell layer through transcytosis. In Fig. 2-C, representative images of eruptions occurring near and without leukocyte cells are presented, using 70 nm Doxil particles and a BxPC3-GFP dorsal skinfold model. Finally, Fig. 2-D shows the colocalization of NPs with endothelial cells to form hotspots along the vessel lining in MMTVPyMT and 4T1 tumor models using 50 nm AuNPs conjugated with Alexa Fluor 647. The scale bars for all panels are provided, and insets are included where appropriate. Common targeting strategies focus on the connections between growth hormones or vitamins and malignant cells. This is because cancer cells often overexpress nutrient receptors in an effort to maintain a steady metabolism despite their rapid division [45]. Epidermal growth factor (EGF) is able to suppress and reduce tumor expression of the EGF receptor, which is overexpressed in a variety of tumor cells, including those that cause breast and tongue cancer. The nutrient folic acid (folie) has also been used for cancer targeting since folate receptors (FRs) are often overexpressed in a range of tumor cells, such as ovarian, endometrial, and renal cancer [13, 14]. As with that, this one is predicated on the same idea. Due to increased metabolic rates, many tumor cells (including those responsible for pancreatic, colon, lung, and bladder cancer) express an increased number of Tf receptors (TfRs). Direct coupling of these targeted agents to nanocarriers delivering chemotherapies, such as medications, has been demonstrated to improve intracellular delivery and treatment effectiveness in animal tumor models. To make matters more complicated, metabolic rate-correlated receptors like folate and Tf are also expressed in rapidly proliferating healthy cells, including fibroblasts, epithelial cells, and endothelial cells. When attempting to target these receptors, this presents a challenge [9]. As a consequence, the medicine's effectiveness and toxicity might suffer from non-specific targeting. Many kinds of murine malignancies benefit from increased intracellular delivery of medications when peptides are utilized as targeting agents, such as arginine-glycine-aspartic acid (RGD), which is the ligand of the cell adhesion integrin v3 on endothelial cells. Nonetheless, RGD binds to integrins different than those seen on cancer cells, including integrins 51 and 41. Since this is a trait, it may limit its usefulness in certain contexts. Heparin sulfate, chondroitin sulfate, and hyaluronan (HA) are examples of extracellular matrices (ECMs) that are overexpressed in tumors and might serve as efficient targets for specific ECM receptors. This is in addition to the targetable cell surface antigens. In vivo, liposomes coated with HA stay in the body longer and can target tumors that have HA receptors [46]. Table 2 provides a comprehensive comparison of various cancer targeting agents, highlighting their respective target antigens, affinity, specificity, binding sites, and targeted therapy types. For instance, monoclonal antibodies (mAbs) targeting CD20 offer a highly specific first-line treatment for non-Hodgkin lymphoma and chronic lymphocytic leukemia, while antibody–drug conjugates (ADCs) target HER2-positive cancer cells for chemotherapy with reduced side effects. Bispecific T cell engagers (BiTEs) show high potency and lower toxicity compared to CAR T cell therapy, although they are limited to CD19-positive cancers. Peptide ligands, aptamers, and nanobodies offer alternative strategies, with their own advantages and limitations, such as low immunogenicity or limited penetration of solid tumors. Other approaches include CAR T cells, radioimmunotherapy (RIT), small molecule inhibitors, and viral vectors, each providing unique advantages in targeting specific cancer types or overcoming resistance. Furthermore, peptide nucleic acids (PNAs), aptamer-drug conjugates (ApDCs), peptide vaccines, and various nanoparticle-based therapies contribute to the diverse landscape of cancer targeting agents, all aiming to optimize efficacy, specificity, and safety while minimizing side effects and resistance development.

**Table 2:** Comparison of cancer targeting agents

**Targeting Agent Type**	**Target Antigen**	**Affinity**	**Specificity**	**Binding Site**	**Targeted Therapy Type**	**Description**	**Novelty**	**Advantages**	**References**
**Monoclonal antibodies (mAbs)**	CD20	High	Specific	Epitope on B-cell surface	Immunotherapy	First-line treatment for non-Hodgkin lymphoma and chronic lymphocytic leukemia	Highly specific to target antigen	Target other healthy cells with similar antigen, Costly production	[48]
**Antibody–drug conjugates (ADCs)**	HER2	High	Specific	Epitope on HER2-positive cancer cells	Chemotherapy	Targeted delivery of cytotoxic agents to HER2-positive cancer cells	Reduced side effects compared to traditional chemotherapy	Limited therapeutic window, Risk of resistance development	[49, 50]
**Bispecific T cell engagers (BiTEs)**	CD19 and CD3	High	Specific	Epitopes on B-cell and T-cell surfaces	Immunotherapy	Redirect T cells to attack CD19-positive B cells	High potency, Lower toxicity compared to CAR T cell therapy	Limited to CD19-positive cancers, Potential for cytokine release syndrome	[51]
**Peptide ligands**	VEGF receptor	Moderate	Specific	Ligand-binding site on VEGF receptor	Anti-angiogenic therapy	Inhibit angiogenesis by blocking VEGF receptor signaling	Low immunogenicity, Easier to produce than mAbs	Short half-life, Rapid clearance	[52]
**Aptamers**	PDGF	High	Specific	Binding site on PDGF	Anti-angiogenic therapy	Inhibit PDGF signaling to block angiogenesis	High binding affinity, Low immunogenicity, Easier to produce than mAbs	Short half-life, Limited in vivo stability	[44]
**Nanobodies**	EGFR	High	Specific	Epitope on EGFR	Immunotherapy	Target EGFR-positive cancer cells for imaging and therapy	Small size, High specificity, High in vivo stability	Limited penetration of solid tumors, Limited capacity for multivalent binding	[53]
**CAR T cells**	CD19	High	Specific	Epitope on B-cell surface	Immunotherapy	Genetically engineered T cells that express a chimeric antigen receptor (CAR) for CD19	High efficacy, Durable response, Curative potential for some hematological malignancies	Risk of severe toxicity including cytokine release syndrome and neurotoxicity, High cost	[54]
**Radioimmunotherapy (RIT)**	CD20	High	Specific	Epitope on B-cell surface	Radiation therapy	Combine the specificity of mAbs with the therapeutic potential of ionizing radiation	Selectively target and destroy cancer cells, Potential for long-term response	Limited to CD20-positive cancers, Risk of toxicity to normal tissue, Complex production process	[48, 54]
**Small molecule inhibitors**	BCR-ABL	High	Specific	Active site of BCR-ABL kinase	Targeted therapy	Inhibit the activity of cancer-promoting proteins	Oral administration, High selectivity, Overcome resistance to traditional chemotherapy	Limited to cancers driven by specific mutations, Development of resistance	[49, 50]
**Viral vectors**	HER2	High	Specific	Epitope on HER2-positive cancer cells	Gene therapy	Deliver therapeutic genes to HER2-positive cancer cells	High specificity and selectivity, Potential for long-term response	Limited to HER2-positive cancers, Potential for toxicity and immune response	[49, 50]
**Peptide nucleic acids (PNAs)**	KRAS	High	Specific	Target site on KRAS mRNA	Gene therapy	Inhibit the expression of cancer-promoting genes	High specificity, Stable in vivo, Overcome resistance to traditional chemotherapy	Limited to cancers driven by specific mutations, Development of resistance	[55]
**Aptamer-drug conjugates (ApDCs)**	PSMA	High	Specific	Binding site on PSMA	Chemotherapy	Targeted delivery of cytotoxic agents to PSMA-positive cancer cells	Reduced side effects compared to traditional chemotherapy, Easier to produce than mAbs	Limited therapeutic window, Risk of resistance development	[44]
**Peptide vaccines**	MUC1	Moderate	Specific	Epitope on MUC1-positive cancer cells	Immunotherapy	Activate the immune system to recognize and attack cancer cells	Induce long-lasting immune responses, Low toxicity	Limited to MUC1-positive cancers, Limited efficacy in solid tumors	[56]
**Liposomes**	Doxorubicin	Low	Non-specific	Passive targeting to tumors through the enhanced permeability and retention (EPR) effect	Chemotherapy	Deliver drugs to tumors with reduced side effects on healthy tissues	Easier to produce than mAbs, Versatile drug delivery system	Limited selectivity, Variable EPR effect in different cancers	[57]
**Gold nanoparticles**	EGFR	Moderate	Specific	Epitope on EGFR	Photothermal therapy	Absorb light to generate heat and destroy cancer cells	High biocompatibility, Versatile drug delivery system	Limited penetration of solid tumors, Limited efficacy in deep tissues	[58]
**Magnetic nanoparticles**	CD44	Low	Non-specific	Magnetic targeting to tumors with external magnetic fields	Chemotherapy	Deliver drugs to tumors with reduced side effects on healthy tissues	Easier to produce than mAbs, Minimal systemic exposure	Limited selectivity, Limited efficacy in deep tissues	[59]
**RNA interference (RNAi)**	Survivin	High	Specific	Target site on survivin mRNA	Gene therapy	Inhibit the expression of cancer-promoting genes	High specificity, Overcome resistance to traditional chemotherapy	Limited to cancers	[60]
**Aptamer-conjugated nanoparticles**	Nucleolin	High	Specific	Binding site on nucleolin	Chemotherapy	Targeted delivery of drugs to nucleolin-positive cancer cells	High specificity, Reduced side effects compared to traditional chemotherapy, Easier to produce than mAbs	Limited to nucleolin-positive cancers, Limited in vivo stability	[44]
**Antibody-nanoparticle conjugates**	CD20	High	Specific	Epitope on B-cell surface	Immunotherapy	Targeted delivery of nanoparticles to CD20-positive cancer cells for imaging and therapy	Increased tumor penetration and retention, High selectivity	Limited to CD20-positive cancers, Risk of immunogenicity	[61]
**Tumor-penetrating peptides**	iRGD	Moderate	Specific	Binding site on integrins and neuropilin-1	Chemotherapy	Enhance the penetration and accumulation of drugs in tumors	High specificity, Overcome barriers to drug delivery in solid tumors	Limited efficacy in deep tissues, Potential for off-target effects	[43]
**Nanobody-drug conjugates**	EGFR	High	Specific	Epitope on EGFR	Chemotherapy	Targeted delivery of cytotoxic agents to EGFR-positive cancer cells	Small size, High specificity, Reduced side effects compared to traditional chemotherapy	Limited to EGFR-positive cancers, Limited capacity for multivalent binding	[44]
**Dual-targeting antibodies**	CD3 and CD20	High	Specific	Epitopes on B-cell and T-cell surfaces	Immunotherapy	Redirect T cells to attack CD20-positive B cells	Increased efficacy, Overcome resistance to monoclonal antibodies	Limited to CD20-positive cancers, Potential for cytokine release syndrome	[61]
**Protein cages**	Ferritin	Low	Non-specific	Passive targeting to tumors through the EPR effect	Drug delivery	Deliver drugs to tumors with reduced side effects on healthy tissues	Easier to produce than mAbs, Biocompatible	Limited selectivity, Variable EPR effect in different cancers	[28]
**Aptamer-siRNA conjugates**	VEGF	High	Specific	Binding site on VEGF	Gene therapy	Inhibit VEGF expression to block angiogenesis	High specificity, Overcome delivery challenges	Limited to VEGF-driven cancers, Variable in vivo stability	[44]
**Therapeutic antibodies**	CTLA-4	High	Specific	Epitope on CTLA-4	Immunotherapy	Block inhibitory signals to activate T cells against cancer cells	High specificity, Durable response, Synergistic with PD-1 blockade	Risk of toxicity, Limited efficacy in solid tumors	[61]
**Bifunctional fusion proteins**	IL-2 and CD25	High	Specific	Epitopes on T-cell and cancer cell surfaces	Immunotherapy	Stimulate T-cell proliferation and activation against cancer cells	Increased efficacy, Reduced toxicity compared to systemic IL-2	Limited to IL-2-responsive cancers, Limited efficacy in solid tumors	[62]

### Proteins as targeting agents

Various proteins, including antibodies and engineered proteins, have been harnessed as targeting agents in nanotechnology-based cancer therapy [63]. These proteins can be designed to recognize specific antigens or receptors overexpressed on cancer cells. For instance, monoclonal antibodies can be conjugated to nanoparticles to enhance their tumor-targeting capabilities [64]. Additionally, protein engineering techniques, such as phage display and recombinant DNA technology, have enabled the development of novel proteins with high specificity for cancer-associated targets [65]. Monoclonal antibodies, derived from hybridoma cells or through recombinant technology, have been extensively utilized to recognize specific antigens or receptors that are overexpressed on the surface of cancer cells. These antibodies can be conjugated to nanoparticles, enhancing their ability to deliver therapeutic agents directly to the tumor site [66]. The key advantage of monoclonal antibodies lies in their high specificity, making them ideal for targeting specific cancer biomarkers. However, they face challenges related to limited tissue penetration and potential immunogenicity, which need to be carefully considered in their clinical application [67]. Engineered proteins, created through recombinant DNA technology, offer a customizable approach to cancer targeting. These proteins can be designed to bind selectively to cancer-associated markers, providing a versatile platform for both targeted therapy and diagnostic imaging [68]. Engineered proteins have the advantage of reduced immunogenicity compared to traditional antibodies. However, their production can be complex and costly, necessitating further optimization to streamline their manufacturing process [69]. Aptamers, another class of targeting agents, are single-stranded DNA or RNA molecules with unique three-dimensional structures that enable them to bind tightly to cancer-specific biomarkers. In vitro selection processes yield aptamers with high specificity, making them valuable tools for targeted drug delivery and imaging. Their reduced immunogenicity compared to antibodies is an attractive feature [70]. However, ensuring their stability in biological environments remains a challenge, requiring ongoing research efforts [71]. Small interfering RNAs (siRNAs) represent a different approach to targeting cancer cells at the genetic level [72]. SiRNAs, synthesized chemically or produced through recombinant technology, can silence genes responsible for cancer cell growth and survival. This precision in gene regulation offers the potential for gene therapy and the inhibition of cancer-related genes [73]. While siRNAs provide a powerful tool, efficient delivery and the risk of off-target effects are issues that need to be addressed [74]. Peptide ligands, often synthesized chemically or produced through recombinant methods, bind to specific cell surface receptors, contributing to targeted cancer therapy and improved cell penetration. Their customizable nature makes them versatile targeting agents, and they hold promise for multi-targeting strategies [75]. Nevertheless, challenges related to their stability in biological environments and efficient delivery systems must be overcome for optimal clinical use [76]. Small organic molecules represent a diverse group of targeting agents that can interact with specific signaling pathways involved in cancer progression. They offer the advantage of diverse chemical structures and drug-like properties, which can be leveraged for cancer therapy [74]. However, optimizing their specificity and selectivity while ensuring stability and delivery to the tumor site remains a focus of ongoing research. Fusion proteins combine the functions of targeting and therapeutic molecules, offering dual-action targeted therapy [76]. Created through recombinant DNA technology, these proteins enhance treatment efficacy while reducing side effects. Their design and production can be complex, necessitating careful consideration in clinical applications [67].

### Nucleic acids as targeting agents

Nucleic acids, specifically aptamers and siRNAs (small interfering RNAs), have emerged as promising targeting agents in the field of cancer therapy [77]. Aptamers are single-stranded DNA or RNA molecules with the unique capability to fold into specific three-dimensional structures. This structural versatility enables them to selectively bind to cancer-specific cell surface biomarkers, making them attractive candidates for targeted therapy [70]. Aptamers offer several advantages, including high specificity for their target biomarkers, low immunogenicity, and the potential for multi-targeting to address heterogeneous cancer populations [78]. However, the challenges associated with aptamer development, such as the selection and optimization of aptamers for specific targets, as well as ensuring their stability in biological environments, remain areas of active research. On the other hand, siRNAs are short double-stranded RNA molecules designed to silence specific genes involved in cancer cell growth and survival. They hold significant promise for personalized cancer therapy by allowing precise control over gene expression [79]. SiRNAs can be incorporated into nanocarriers for targeted delivery to cancer cells, offering the advantage of selective gene silencing. This approach can be particularly valuable for cancers driven by specific genetic mutations or overexpression of oncogenes [77]. However, efficient intracellular delivery of siRNAs remains a challenge, as does minimizing off-target effects that can potentially disrupt normal cellular processes [80]. Examples of nucleic acids in cancer therapy include the use of aptamers targeting specific cancer-associated biomarkers. For instance, the PSMA aptamer has been employed for prostate cancer targeting, while the MUC1 aptamer has shown promise in breast cancer targeting [81, 82]. In the case of siRNAs, researchers have explored their potential in targeting critical genes in cancer, such as the use of siRNAs against the BCR-ABL fusion gene in chronic myeloid leukemia and siRNAs targeting KRAS mutations in pancreatic cancer. These nucleic acid-based targeting agents represent innovative approaches to cancer therapy, offering the potential for enhanced specificity and reduced off-target effects [79]. However, addressing challenges related to aptamer and siRNA development, intracellular delivery, and safety will be crucial for realizing their full therapeutic potential in clinical settings. Researchers continue to work on optimizing these strategies and advancing the field of nucleic acid-based cancer therapeutics [82, 83].

## Availability of nanocarriers, section

**Fig. 3:**
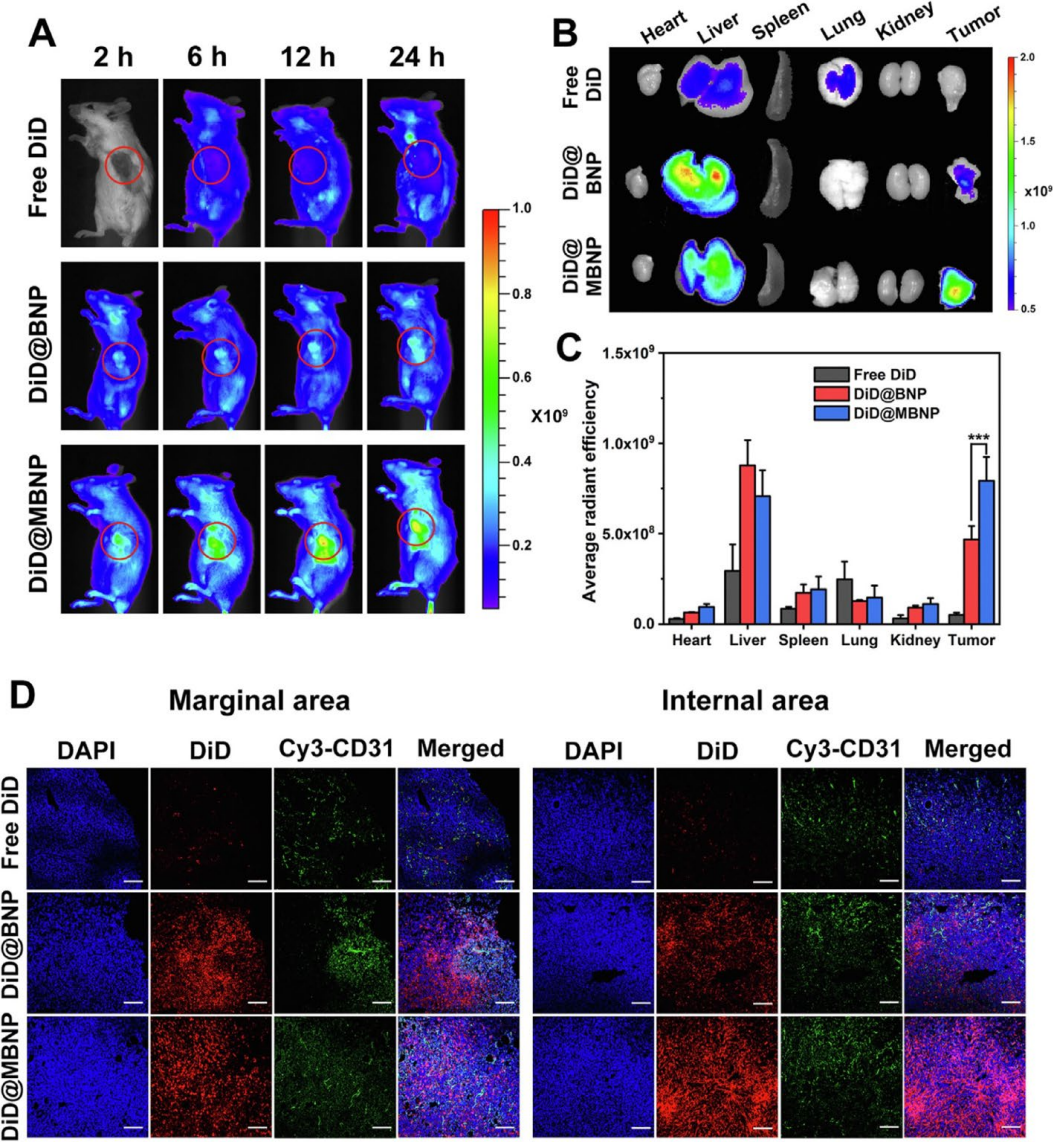
The results of the in vivo biodistribution of nanocarriers. The study involved the use of DiD-loaded formulations, and the images obtained from 4T1 tumor-bearing mice were taken at different times post-administration. The red circles in the images indicate the tumor sites. Additionally, ex vivo imaging of isolated tumors and organs from the mice was performed 24 h after administration. The semiquantification of fluorescence intensity was also done, and the results showed statistically significant differences (*P* < 0.05, *P* < 0.01, *P* < 0.001) among the three formulations. Finally, the fluorescent distribution of Free DiD, DiD@BNP, and DiD@MBNP in the frozen sections of tumors was analyzed, with blue indicating the cell nucleus, red indicating DiD, and green indicating CD31. The scale bars used in the images are 100 mm. Reprint from [111] with a permission from Elsevier

Nanocarriers are substances between one and one hundred nanometers in size, and they may carry a wide variety of drugs and imaging agents. They may be employed for targeting thanks to the high ligand density that can be established on their surfaces according to their huge surface area in relation to their volume [84]. In a recent study conducted by Sultan et al., significant progress has been made in the development of targeted delivery formulations for combating cancer. Specifically, the study focused on the characterization of cisplatin-loaded chitosan nanoparticles (CCNP) and cisplatin-loaded chitosan nanoparticles surface-linked to rituximab (mAbCCNP). These formulations exhibited notable physicochemical properties, with CCNP having a zetapotential (ZP) value of 30.50 ± 5.64 mV and a particle size of 308.10 ± 1.10 nm, while mAbCCNP had a ZP value of 26.90 ± 9.09 mV and a slightly larger particle size of 349.40 ± 3.20 nm. Importantly, both CCNP and mAbCCNP demonstrated controlled release kinetics of cisplatin, suggesting their potential as effective delivery systems. In vitro cytotoxicity studies on MCF-7 ATCC human breast cancer cells revealed that CCNP exhibited significant cytotoxicity with an IC50 of 4.085 ± 0.065 µg/mL, while mAbCCNP, designed for targeted delivery, did not induce any cytotoxic effects. Although the results indicated that CCNP was more successful due to rituximab's lack of specificity against MCF-7 ATCC human breast cancer cells, this study underscores the promising role of nanocarriers in cancer treatment, offering a potential avenue for more effective and targeted therapy [85]. Nanocarriers may also be used to increase the local concentration of the medication by transporting the medicine in the nanocarrier and releasing it slowly once the nanocarrier has linked to its target. Nanocarrier properties such as size, shape, surface charge, surface functionalization, drug payload, biodegradability, and shape stability directly influence the interaction with physiological factors like blood flow rate, lymphatic drainage, plasma protein corona, and renal function. The blood circulation time of nanocarriers, including half-life and clearance rate, protein binding, and tissue-specific accumulation, play a crucial role in determining their biodistribution. Tumor microenvironment factors, such as tumor perfusion, extracellular matrix, pH gradients, and cellular uptake, also impact the delivery and effectiveness of nanocarriers. Lastly, the administration route, encompassing intravenous, intratumoral, oral, local, and active targeting methods, significantly affects the biodistribution of nanocarriers within the body. Nanocarriers are a versatile and innovative approach to drug delivery with distinct characteristics and advantages. Their nanoscale size, controlled release kinetics, biocompatibility, targeted drug delivery capabilities, and long-term stability make them a promising choice for enhancing the precision and effectiveness of drug therapy in various medical applications [80]. Controlled release kinetics is another key feature of nanocarriers. These systems provide precise control over the rate and duration of drug release, making them ideal for sustained drug delivery. For example, nanocarriers based on polymers like PLGA (Poly(lactic-co-glycolic acid)) can offer prolonged drug release, which is essential for maintaining therapeutic drug levels in the body over extended periods. Biocompatibility is a vital advantage of nanocarriers. These systems are designed to be non-toxic and to minimize adverse effects on the body. This characteristic reduces the risk of immune responses and makes nanocarriers a safe option for drug delivery [78]. Lipid-based nanocarriers, for instance, have demonstrated high biocompatibility, making them suitable for various pharmaceutical applications [86]. Targeted drug delivery is a hallmark feature of nanocarriers. They have the unique ability to deliver drugs specifically to targeted cells or tissues, enhancing drug efficacy and reducing toxicity to healthy tissues [80]. Antibody–drug conjugates, a type of nanocarrier, exemplify this feature, as they are designed to selectively target cancer cells, thereby improving the precision of cancer therapy. Long-term stability is also a benefit of nanocarriers. These systems can extend the shelf-life of drugs and maintain drug stability over time. For instance, polymeric micelles, a type of nanocarrier, have demonstrated excellent stability, ensuring that pharmaceutical agents remain effective even after prolonged storage [86]. Table 3 highlights the various factors affecting nanocarrier biodistribution, which can be categorized into nanocarrier properties, physiological factors, blood circulation time, tumor microenvironment, and administration route. Figure 3 displays the results of an in vivo biodistribution study of nanocarriers. The study utilized DiD-loaded formulations, and images were obtained from mice with 4T1 tumors at various times after administration. The red circles in the images indicate the tumor sites. Furthermore, the study involved ex vivo imaging of isolated tumors and organs from the mice 24 h after administration. The semiquantification of fluorescence intensity was conducted, revealing significant differences (*P* < 0.05, *P* < 0.01, *P* < 0.001) among the three formulations. Finally, the distribution of Free DiD, DiD@BNP, and DiD@MBNP was examined in the frozen sections of tumors, with blue indicating the cell nucleus, red indicating DiD, and green indicating CD31. The scale bars used in the images are 100 mm. Overall, the results of this study provide valuable insights into the biodistribution of nanocarriers, which can inform the development of more effective therapeutic interventions for cancer treatment. Nanocarriers include a wide variety of different structures, including polymerconjugates, polymeric nanoparticles, lipid-based carriers like liposomes and micelles, dendrimers, carbon nanotubes, and gold nanoparticles (including nanoshells and nanocages). Medication delivery, imaging, photothermal ablation of malignancies, radiation sensitizers, apoptosis detection, and sentinel lymph node mapping are just some of the many uses for these nanocarriers that have been studied [87]. The use of these conjugates is very helpful for focusing on tumor blood vessels. Anti-endothelial immunoconjugates, fusion proteins, and caplostatin, the first polymer-angiogenesis inhibitor conjugate, are all examples of these molecules.Chemically conjugated polymers containing medicines are typically considered to be new chemical entities (NCEs) [88]. This is due to the fact that their pharmacokinetic characteristics vary greatly from those of the original drug. Polymer-drugconjugates have been developed primarily using just four medicines (doxorubicin, camptothecin, paclitaxel, and platinate) and four polymers (N-(2-hydroxylpropyl) methacrylamide (HPMA)copolymer, poly-L-glutamic acid, poly(ethylene glycol) (PEG), and Dextran) [36]. Despite several new pharmacological targets and cutting-edge chemicals, the use of polymers in the creation of nanoparticle-based drug carriers has been the subject of the vast majority of research. Adsorption of anticancer drugs to polyalkylcyanoacrylate nanoparticles has been recorded as far back as 1979. This utilization dates back to when these particles were first developed for use in the treatment of cancer. The article was among the first to detail their use in cancer treatment [15]. Experiments on tissue distribution and efficacy using a tumor model were conducted after Couvreur et al. revealed the release mechanism of the medications from the polymer in calf serum. This finding allowed for the development of doxorubicin-loaded nanoparticles, which were tested in the middle of the 1980s [2, 3]. To encapsulate pharmaceuticals without chemical alteration, polymeric nanoparticles may be made from either synthetic polymers like poly(lactic acid) (PLA) and poly(lactic co-glycolic acid) or from natural polymers like chitosan and collagen. Nanoparticles may originate from either synthetic or natural polymers [89]. The drugs may be released gradually over time by a variety of mechanisms, including surface or bulk erosion, diffusion through the polymer matrix, swelling followed by diffusion, and environmental response. Several types of multifunctional polymeric nanoparticles are already being evaluated in both pre-clinical and clinical settings [6]. The usage of polymer-based nanocarriers raises concerns due to polymers' inherent structural heterogeneity, which is shown, for example, in a high polydispersity index (the ratio of the weight-and-number-average molecular weight, Mw/Mn) [15]. However, there have been isolated cases of polymeric nanoparticles exhibiting a nearly homogeneous size distribution. Lipid-based carriers have several desired biological properties, including universal biocompatibility, biodegradability, drug isolation from the environment, and the capacity to entrap hydrophilic and hydrophobic drugs. The size, charge, and surface functionality of lipid-based carriers may be easily modified by incorporating agents into the lipid membrane or modifying the surface chemistry [90]. There are several approaches to achieving this goal. Some examples of amphiphile-based particles include micelles, liposomes, and polymersomes. One or more concentric lipid bilayers enclose an inner aqueous phase to form spherical structures known as liposomes [57]. These structures are self-closing and sphere-shaped. Today, regulatory authorities have provided their stamp of approval to enable liposomes to include a broad array of chemotherapeutics. Polymersomes are made up of synthetic polymer amphiphiles, most of which are PLA-based copolymers. Although their design is similar to that of liposomes, polymersomes are not comprised of lipids [57]. On the other hand, similar to the situation with polymer therapies, there are presently no treatments that have been clinically authorized that entail active cellular targeting for lipid-based carriers [57]. Pharmaceutical carriers for water-insoluble medications have been successfully implemented via the use of micelles, which are self-assembling closed lipid monolayers with a hydrophobic core and a hydrophilic shell [91]. Hydrophilic micelles are encased in a hydrophobic core. They belong to the class of amphiphilic colloids, which are able to self-assemble from amphiphilic or surface-active chemicals (surfactants) under certain circumstances (such as concentration and temperature) [91]. Clinical trials are now being conducted on polymeric micelles like NK911, a block copolymer comprising PEG and poly (aspartic acid). NK911 was studied as a possible treatment for advanced pancreatic cancer; it consists of a bound doxorubicin fraction (45%) and a free drug [32]. A micelle NK105, which contains the drug paclitaxel, has also been investigated as a possible carrier for the treatment of cancers of the pancreas, colon, and stomach. The challenges that arise from utilizing lipid-based nanocarriers are indicative of those that arise when using other focused nanocarriers, such as polymeric nanoparticles [61]. For instance, the reticuloendothelial defense system efficiently clears the bloodstream of injected particles regardless of the particles' composition [29]. The non-specific absorption by the mononuclear phagocytic system (MPS) and the instability of the carrier, which may result in burst drug release, are further challenges that need to be addressed before these carriers may be employed in clinical settings [92]. Because of their extensive background, liposomal carriers are a good example of the challenges and solutions that have been explored throughout the development of nanocarriers [93]. By stabilizing and shielding micelles and liposomes against opsonization, the process by which plasma protein deposition signals Kupffer cells in the liver to remove the carriers from circulation, PEG, for example, has been demonstrated to extend the duration a chemical spends in circulation [94]. However, two examples of liposomes used in clinical settings are the PEG-free Daunosome and Myocet, which have a diameter of 80–90 nm. Even though not as much as PEGylated liposomes like Doxil and Caelyx, these liposomes have been shown to have longer circulation times [94]. In addition to the need for rapid clearance, the rapid burst release of the chemotherapeutic drugs from the liposomes presents a challenge. For instance, doxorubicin may have been encapsulated in the liposomal aqueous phase with an ammonium sulfate gradient to avoid this phenomenon [93]. This method results in the stable trapping of the medication, with little leakage of the drug during circulation; this holds true even after prolonged circulation [93, 94]. Clinical investigations have revealed that liposomal systems accumulate preferentially in tumors, and the toxicity of the cargo they transport is much reduced as a result of the EPR effect. A liposome that circulates for a long time may lead to the drug being released in an undesirable region, a phenomenon known as extravasation [95]. Most patients who receive PEGylated liposomal doxorubicin report experiencing palmar-plantar erythrodysesthesia (PPE), also called the hand-foot condition. Dosage and administration schedule adjustments may help patients who have PPE, a dermatologic toxicity reaction. PPE is a side effect that may happen after receiving high dosages of many types of chemotherapy at once [95]. Additional challenges with liposome application in clinical settings include the high production cost of liposomes, the quick oxidation of certain phospholipids, and the lack of controlled-release characteristics in encapsulated medications. Using a "polymercore/lipid shell" (a combination of polymers and phospholipids) as a delivery agent may allow for the synchronized release of two distinct drugs [96]. Once the nanoparticle has been localized to the tumor site through the EPR effect, it will begin to produce both an anti-angiogenesis agent from its outer phospholipid shell and a chemotherapeutic substance from its inner polymeric nanoparticle in response to local hypoxia [97]. Reduced toxicity and improved anti-metastatic effects were shown in two different mouse tumor models using this approach, demonstrating the value of a mechanism-based design for targeted nanocarriers [96]. Organic nanoparticles include dendrimers, viral capsids, and nanostructures produced from biological building materials like proteins, Abraxane, an albumin-bound paclitaxel nanoparticle formulation, was approved by the FDA in 2005 as a second-line therapeutic option for patients with metastatic breast cancer [98]. Abraxane was created as an answer to the insoluble problems seen with paclitaxel. As a result of its use, dangerous solvents like Cremophor EL (polyoxyethylated castor oil) are no longer required for the delivery of Taxol. Creating dendrimers from scratch is a cutting-edge topic in polymer chemistry [95]. Dendrimers are manmade macromolecules with a branching, tree-like structure. For several reasons, including their small size (5 nm), high water solubility, well-defined chemical structures, biocompatibility, and rapid clearance from the blood through the kidneys, polyamidoamine dendrimers have been shown to have potential for use in biomedical applications [99]. Dendrimer-methotrexate conjugates delivered in vivo by multivalent targeting have been shown to reduce tumor development by a factor of ten. This may be compared to the shrinkage of tumors that occurs when free systemic methotrexate is administered at the same molar concentration. This finding prompted other preliminary studies, and many different dendrimers are being looked at as potential cancer therapies. Additional resources provide a comprehensive overview of these dendrimers [99]. Despite their potential advantages, large-scale production of dendrimers is complicated by their higher price tag compared to other nanoparticles and the necessity for many iterations during the synthesis process [100]. Metal nanoparticles make up the bulk of inorganic nanoparticles and may be produced with near-perfect monodispersity [29]. Inorganic materials have been the focus of many studies for applications including magnetic resonance imaging and high-resolution superconducting quantum interference devices. Furthermore, inorganic particles may be functionalized to include specific chemicals and pharmaceuticals. Some specialized kinds of inorganic nanoparticles, such as nanoshells and gold nanoparticles, have just lately been manufactured [29]. The same carrier might be used for both imaging and therapy in nanoshells on the order of 100–200 nm in size. They have a silica core and a metal exterior. Nanoshells' optical resonances can be adjusted to absorb or scatter electromagnetic radiation across a wide range of frequencies [23]. The near-infrared region (820 nm, 4 W cm-2) of the electromagnetic spectrum allows for the most efficient transmission of light through tissue [23]. Absorbing nanoshells may be used in treatments that rely on hyperthermia. Nanoshells would be used to absorb radiation and heat the surrounding cancer tissue in these types of therapies [101]. The enhanced contrast that scattering nanoshells provide makes them a useful tool for imaging applications. The new cancer treatment uses infrared (NIR) light absorption by nanoshells as its basis. Tumors implanted in mice are killed selectively thanks to the rapid local heating triggered by this therapy [101]. In tissues heated past the point of thermal damage, coagulation, cell shrinkage, and loss of nuclear staining were observed [93]. Despite being treated at the same temperature, control tissues showed no signs of damage. Similar methods use gold nanocages, which are even smaller than nanoshells (less than 50 nm) [90, 102]. These gold nanocages may be engineered to generate heat in response to NIR light. As a result, they may be beneficial for hyperthermia-based therapies. In contrast to nanoshells and nanocages, pure gold nanoparticles may be easily manufactured and controlled [103, 104]. Non-specific interactions that generate toxicity in healthy tissues may limit the usefulness of many types of nanoparticles. However, the use of inorganic particles for photo-ablation greatly reduces the amount of non-specific toxicity that may occur due to light's localized nature [90, 102]. However, for systemic targeting of particular cancer cells, inorganic particles may not provide any benefits over other forms of nanoparticles. Inorganic particles that build up in the body can cause long-term harm because they don't break down and aren't small enough for the body to get rid of them easily [102].

**Table 3:** Factors affecting nanocarrier biodistribution

**Nanocarrier Properties**	**Physiological Factors**	**Blood Circulation Time**	**Tumor Microenvironment**	**Administration Route**	**References**
**Size and shape**	Blood flow rate and vessel permeability	Half-life and clearance rate	Tumor perfusion and oxygenation	Intravenous, intratumoral, intraperitoneal, etc	[105]
**Surface charge and coating**	Lymphatic drainage and lymph node accumulation	Protein binding and opsonization	Extracellular matrix and cell adhesion molecules	Oral, nasal, pulmonary, transdermal, etc	[106]
**Surface functionalization**	Plasma protein corona and immune system response	Blood–brain barrier penetration	pH and redox gradients	Local, regional, systemic, etc	[106]
**Drug payload and release mechanism**	Renal and hepatic function	Tissue-specific accumulation and clearance	Cellular uptake and trafficking	Active targeting, passive targeting, etc	[106, 107]
**Biodegradability and toxicity**	Interstitial fluid pressure and flow	Cellular metabolism and excretion	Tumor heterogeneity and evolution	Single dose, repeated dose, etc	[56, 108]
**Shape stability**	Inflammatory response and cytokine release	Vascular permeability and leakiness	Stromal cells and immune cells	Direct injection, inhalation, etc	[109]
**Aggregation and stability**	Oxygen and carbon dioxide transport	Extravasation and interstitial diffusion	Hypoxia and acidity	Local hyperthermia, phototherapy, etc	[84]
**Magnetism and targeting**	Enzyme activity and expression	Receptor density and internalization	Angiogenesis and lymphangiogenesis	Ultrasound, magnetic, etc	[110]
**Encapsulation and surface modification**	Protease activity and inhibition	Immune checkpoint expression and regulation	Immunosuppression and immunostimulation	Combination, alternating, etc	[109]
**Controlled release and activation**	Nutrient and oxygen deprivation	Apoptosis and necrosis	Resistance and tolerance	Adjuvant therapy, radiation, etc	[45]

## Multidrug resistance and its consequences

Nanoprecipitation and self-assembly techniques, on the other hand, offer medium-scale manufacturing with moderate cost and quality control. Large-scale production is achievable through methods such as spray drying and solvent casting, although they may compromise quality control and flexibility [112–114]. Innovations like flash nanoprecipitation and hydrodynamic focusing microfluidics yield high-quality nanoparticles with excellent control and flexibility, while hot melt extrusion caters to large-scale production with high quality control. Each manufacturing method has its specific applications, and researchers must carefully weigh the factors of scale, cost, quality control, yield, and flexibility when selecting the most suitable technique for their needs. Table 4 presents a comparison of various nanocarrier manufacturing techniques, each with its unique set of advantages and drawbacks. Emulsion and microfluidics methods, for example, boast high quality control and flexibility but are limited to small-scale production. Pharmaceuticals can also be delivered to cells via targeted nanocarriers that are absorbed by the cells rather than through diffusion. This technique has the potential to allow selected carriers to circumvent the action of multiple drug resistance transporters (MDRtransporters), which are integral membrane proteins. MDRtransporters are implicated in the efflux of many chemotherapeutic agents from cancer cells. An increased concentration of enzymes that may neutralize chemotherapy drugs has been related to the complex molecular basis of cancer medicine resistance [115]. The discovery of this fact is due to the study of the molecular basis of cancer medication resistance. However, most of the time, this occurs due to an overexpression of MDR transporters, which actively pump chemotherapeutic drugs out of the cell and reduce the quantity of drug present inside the cell to levels below the deadly threshold [102]. In a recent study conducted by Maliyakkal et al., a promising approach to enhance the efficacy of cisplatin in the treatment of glioblastoma multiforme (GBM) was investigated. Cisplatin is a potent anticancer drug commonly used for GBM therapy; however, its clinical effectiveness has been hampered by low therapeutic ratios, toxicity, and multidrug resistance (MDR) issues. To address these limitations, the researchers developed a novel system utilizing cisplatin-loaded polymeric nanoplatforms (CSP-NPs) designed for active targeting within GBM. These CSP-NPs were characterized extensively and demonstrated a smooth surface, appropriate particle size, zeta potential, polydispersity index, drug entrapment efficiency, and drug content. Importantly, CSP-NPs exhibited an initial burst effect followed by sustained drug release, resulting in dose and time-dependent cytotoxicity and apoptosis induction in human GBM cells. Furthermore, these nanocarriers significantly enhanced the uptake and intracellular accumulation of anticancer drugs while also reversing the activity of MDR transporters (ABCB1 and ABCG2) in GBM cells. This research highlights the potential of nanocarriers as a promising strategy to overcome the limitations of current chemotherapy approaches in the treatment of GBM, offering a more effective and specific therapeutic option [116]. Medication-resistant cells that strongly express MDR transporters will survive chemotherapy treatment because they are more sensitive to the effects of the drug. It's because not all cancer cells express the MDR transporters. It is possible that chemotherapy will not be effective against recurring cancers since the tumor population is dominated by drug-resistant cells. The MDR transporters that have gotten the greatest interest from scientists include P-glycoprotein (also known as MDR1 or ABCB1), the multidrug resistance-related proteins (MRPs), of which MRP1 (or ABCC1) has been investigated the most, and the breast cancer resistance protein (ABCG2) [5]. Though structurally diverse, these proteins all have the same function of clearing chemotherapeutic drugs from the body's cells. Multiple studies have shown that MDR transporters may be avoided with the use of nanocarriers. SP1049C is a doxorubicin-containing non-ionic (sometimes called a pluronic or poloxamer) block-copolymer. It consists of a hydrophobic body and a hydrophilic extension [13]. SP1049C is now being evaluated in clinical trials for its potential to reverse p-glycoprotein-mediated drug resistance in a mouse model of leukemia. Cell uptake of doxorubicin-loaded liposomes through the foliate receptor was shown to be unaffected by P-glycoprotein (Pgp)-mediated drug efflux in an MDR cell line. This contrasts with the absorption of free doxorubicin. These results may be attributed to folic acid receptors [57]. In resistant human myelogenous leukemia cell lines, cytotoxicity was increased when vincristine-loaded lipid nanoparticles were coupled to an anti-Pgp monoclonal antibody (MRK-16). The Pgp-mediated efflux of vincristine is inhibited by MRK-16, which causes this reaction. The goal was to undo the effects of MDR. Possible answers to the issue of MDR have been looked at, and these include polymer therapeutics, polymeric nanoparticles, lipid nanocapsules, and micelles. These tests have been done in cell cultures or in animal models of cancer. Targeted nanocarriers for selective drug delivery and multidrug resistance pump inhibitors may be able to solve some of the problems caused by resistant tumors [48].

**Table 4:** Comparison of nanocarrier manufacturing techniques

**Manufacturing Method**	**Scale**	**Cost**	**Quality Control**	**Yield**	**Flexibility**	**References**
**Emulsion**	Small	Low	High	Low	High	[117]
**Nanoprecipitation**	Medium	Moderate	Moderate	Moderate	High	[118]
**Microfluidics**	Small	High	High	High	High	[118]
**Electrospinning**	Small	High	High	High	Low	[118]
**Self-Assembly**	Medium	High	Low	High	Moderate	[119]
**Spray Drying**	Large	Moderate	Low	High	Low	[120]
**Solvent Casting**	Large	Moderate	High	Moderate	Moderate	[120]
**Superparamagnetic Iron Oxide Nanoparticle Synthesis by Co-precipitation**	Small	Low	High	High	Low	[121]
**Microemulsion Method for Solid Lipid Nanoparticle Synthesis**	Small	Moderate	High	Moderate	High	[57]
**Flash NanoPrecipitation for Polymer Nanoparticle Synthesis**	Medium	High	High	High	High	[122]
**Hydrodynamic Focusing Microfluidics for Liposome Synthesis**	Small	High	High	High	High	[123]
**Electrospray for Protein Nanoparticle Synthesis**	Small	High	High	Low	High	[100]
**Ultrasonic Atomization for Polymeric Micelle Synthesis**	Medium	Moderate	Moderate	Moderate	Moderate	[123]
**Hot Melt Extrusion for Lipid Nanoparticle Synthesis**	Large	High	High	High	Moderate	[57]
**Flash Nanocomplexation for RNA Nanoparticle Synthesis**	Medium	High	Moderate	High	High	[100]

• Manufacturing Method: Different nanocarrier manufacturing methods used in the industry

• Scale: The manufacturing scale range of each method, categorized as small, medium, and large

• Cost: The estimated cost range of manufacturing nanocarriers by each method, categorized as low, moderate, and high

• Quality Control: The level of quality control required for each method, categorized as low, moderate, and high

• Yield: The expected nanocarrier yield by each method, categorized as low, moderate, and high

• Flexibility: The degree of flexibility offered by each manufacturing method in terms of nanocarrier properties and customizability, categorized as low, moderate, and high

## Cancer therapy using nanomaterials

**Fig. 4:**
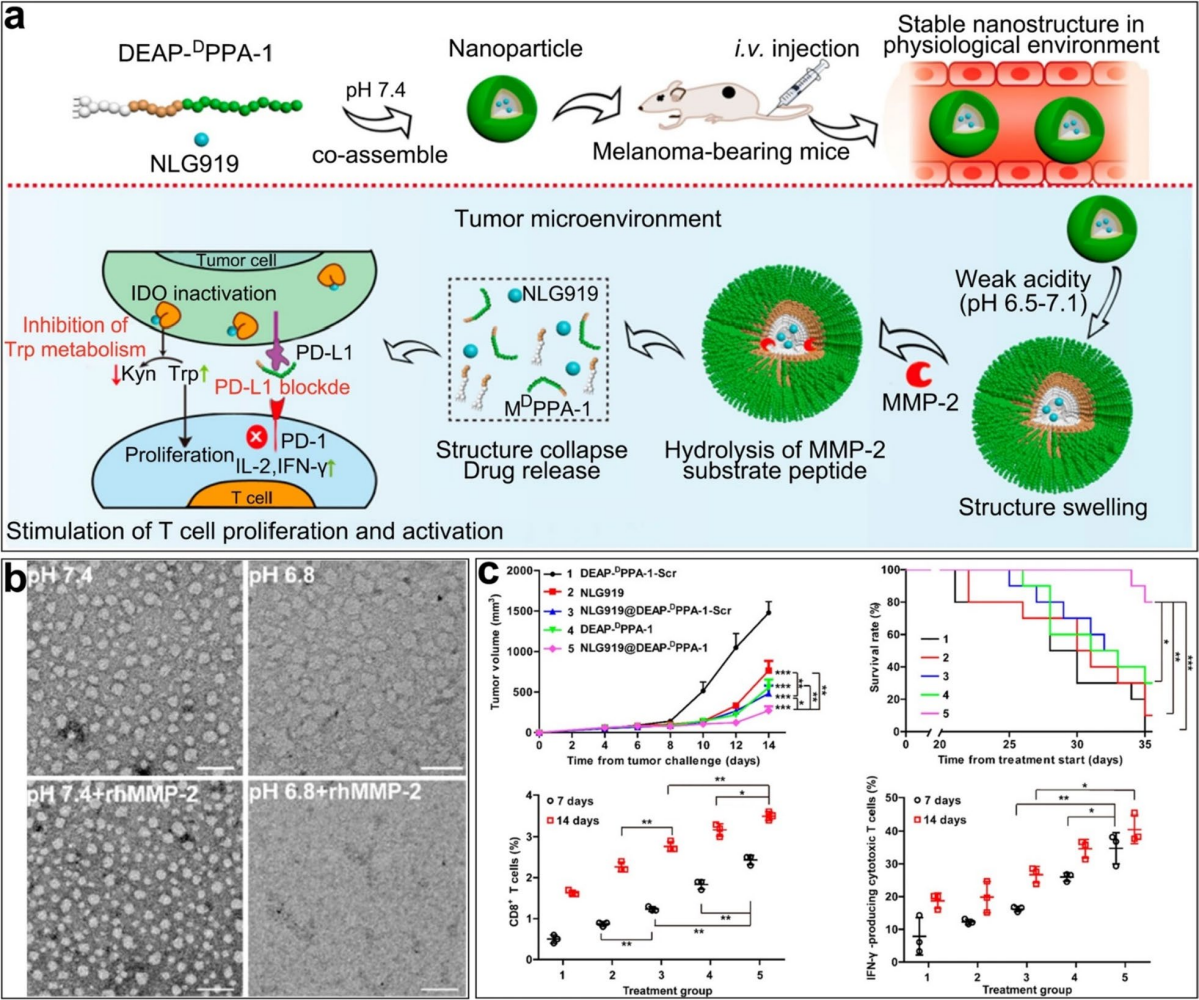
The schematic of cancer immunotherapy using NLG919@DEAP-DPPA-1 nanoparticles. The multifunctional peptide demonstrates its antitumor mechanism in the tumor microenvironment. Transmission electron microscopy images of nanoparticles under various pH conditions, with or without recombinant human MMP-2 (rhMMP-2), are shown. The treatment efficacy of peptide nanoparticles and the measurement of CD8 + T cells in melanoma-bearing mice are also presented. The scale bars for the TEM images are 100 nm. Reprint from [129] with a permission from Springer Nature

**Fig. 5:**
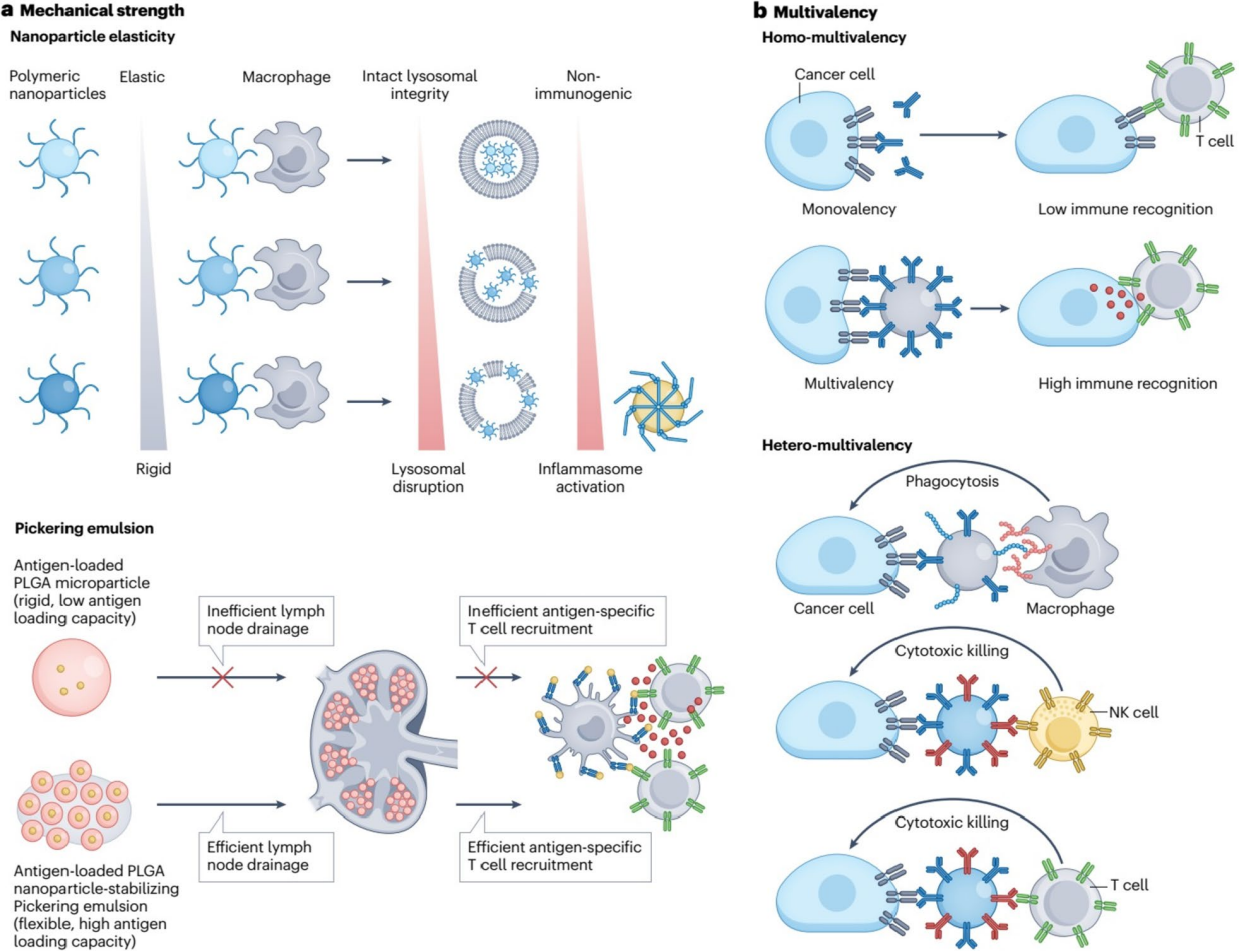
The impact of mechanical strength and multivalency of nanomaterials on cancer immunotherapy outcomes. a, the stiffness of polymeric nanoparticles influences the stability of lysosomes, which is related to inflammasome activation in cancer immunotherapy. The flexibility of these nanomaterials governs their adaptability and lateral movement, which in turn affects their ability to load antigens and target lymph nodes. b, Nanoparticles with multiple binding sites can trigger immune signaling or promote the attraction of immune cells within the tumor environment. Nanoparticles with uniform multiple binding sites improve T cell immune recognition by inhibiting immune checkpoints. In contrast, nanoparticles with varied multiple binding sites facilitate interactions between cancer cells and immune cells, resulting in tumor-specific immune responses. Reprint from [130] with a permission from Springer Nature

Cancer is a devastating disease that affects millions of people worldwide. Traditional cancer therapies such as chemotherapy and radiation therapy have significant limitations, including non-specific targeting and high toxicity to healthy cells. The development of nanotechnology has offered new approaches for cancer detection and treatment. Nanotechnology refers to the science of manipulating materials at the nanoscale, typically in the range of 1–100 nm [124, 125]. The small size of nanomaterials allows them to interact with cells and tissues in unique ways, enabling them to be used for targeted drug delivery and cancer therapy. Nanocarriers, which are tiny particles that can be loaded with therapeutic agents, are a key area of research in the field of nanotechnology for cancer treatment [61]. One of the challenges in the development of nanocarriers for cancer therapy is the ability to target cancer cells specifically, while avoiding healthy cells. Passive targeting methods, such as the enhanced permeability and retention (EPR) effect, take advantage of the unique characteristics of tumor cells, such as their leaky blood vessels and poor lymphatic drainage [126]. In contrast, active targeting involves modifying the surface of the nanocarrier with specific targeting molecules that bind to receptors on cancer cells, enabling them to be more effectively targeted. Nanotechnology has been used in various forms for cancer therapy, including polymeric nanoparticles, monoclonal nanoparticle antibodies, lipid-based nanomaterials, nanoemulsions, dendrimers, and nano-scale carbon materials [126]. These materials have been used to improve drug delivery to cancer cells, reduce toxicity to healthy cells, and improve patient outcomes. Liposomes, for instance, show sustained release of doxorubicin through a pH gradient mechanism in acidic environments, maintaining stability. Polymeric nanoparticles offer controlled diffusion of paclitaxel in neutral pH conditions, remaining stable throughout the process. Dendrimers, on the other hand, release methotrexate through swelling in a pulsatile manner under basic conditions, though they are unstable. Nanoemulsions demonstrate pulsatile partitioning of docetaxel in acidic environments, while gold nanoparticles can release curcumin through a photothermal-triggered mechanism under neutral pH conditions. These nanocarriers exhibit various release rates, pH sensitivities, and stabilities, contributing to their diverse applications in drug delivery systems. Table 5 outlines various nanocarrier drug release kinetics, which have been studied to optimize the delivery of different drug types. One of the most promising areas of research in cancer therapy and nanotechnology development is the use of immunotherapy in combination with nanocarriers. Immunotherapy is a type of cancer treatment that stimulates the body's immune system to attack cancer cells [127]. By combining immunotherapy with nanocarriers, it is possible to increase the efficacy of the treatment and reduce toxicity to healthy cells. Another promising area of research is the development of nanotechnology-based diagnostic tools for cancer detection. Figure 4 depicts the schematic of cancer immunotherapy using NLG919@DEAP-DPPA-1 nanoparticles. The multifunctional peptide showcased its antitumor mechanism in the tumor microenvironment. The figure also displays transmission electron microscopy images of the nanoparticles under various pH conditions, with or without recombinant human MMP-2 (rhMMP-2). Additionally, the treatment efficacy of peptide nanoparticles and the measurement of CD8 + T cells in melanoma-bearing mice are presented. The scale bars for the TEM images are 100 nm, providing a clear visual representation of the nanoparticles. Nanoscale sensors and probes can be used to detect cancer cells in blood samples or to visualize tumors in the body, enabling early diagnosis and treatment. Despite the many advantages of using nanotechnology in cancer therapy, there are still challenges that need to be addressed [61]. These challenges include the development of more efficient and cost-effective nanocarriers, ensuring the safety of nanocarriers in humans, and improving the understanding of tumor biology. The development of new and innovative nanocarriers and the continued improvement of targeted drug delivery systems will be essential to the future of cancer therapy and the continued development of nanotechnology [127]. The development of nanotechnology has offered a promising new approach to cancer detection and treatment. With further research and development, it may be possible to create targeted nanocarriers that are highly effective at delivering drugs to cancer cells, minimizing side effects, and improving the overall efficacy of cancer treatment. The use of nanotechnology in cancer therapy has the potential to revolutionize the field of oncology and offer renewed hope to patients with this devastating disease [128]. Figure 5 highlights the critical role of mechanical strength and multivalency of nanomaterials in shaping cancer immunotherapy outcomes. The stiffness of polymeric nanoparticles plays a pivotal role in determining lysosome stability, which directly influences inflammasome activation, a crucial step in cancer immunotherapy. Flexible nanomaterials exhibit better adaptability and lateral movement, optimizing antigen loading and targeting lymph nodes more efficiently. Furthermore, nanoparticles with multiple binding sites can significantly enhance immune signaling or attract immune cells to the tumor environment. Uniform multiple binding sites on these nanoparticles improve T cell immune recognition by inhibiting immune checkpoints. On the other hand, nanoparticles featuring varied multiple binding sites foster interactions between cancer cells and immune cells, ultimately leading to tumor-specific immune responses.

**Table 5:** Nanocarrier drug release kinetics

**Nanocarrier Type**	**Drug Type**	**Release Mechanism**	**Release Rate**	**pH Sensitivity**	**Stability**	**References**
**Liposomes**	Doxorubicin	pH Gradient	Sustained	Acidic	Stable	[13]
**Polymeric nanoparticles**	Paclitaxel	Diffusion	Controlled	Neutral	Stable	[90, 102]
**Dendrimers**	Methotrexate	Swelling	Pulsatile	Basic	Unstable	[99]
**Nanoemulsions**	Docetaxel	Partitioning	Pulsatile	Acidic	Stable	[117]
**Gold nanoparticles**	Curcumin	Photothermal	Triggered	Neutral	Stable	[90, 102]
**Iron oxide nanoparticles**	Doxorubicin	Magnetic field	Sustained	Neutral	Stable	[121]
**Lipid-based**	Paclitaxel	Diffusion	Sustained	Acidic	Stable	[57]
**Polymer-lipid hybrid**	Cisplatin	Hydrolysis	Burst	Neutral	Unstable	[57]
**Carbon-based**	Cisplatin	Adsorption	Triggered	Neutral	Unstable	[90, 102]
**Gold nanorods**	Doxorubicin	Photothermal	Pulsatile	Neutral	Stable	[90, 102]
**Silica nanoparticles**	Curcumin	pH-Responsive	Sustained	Neutral	Stable	[88]
**Polymer nanoparticles**	Paclitaxel	Erosion	Sustained	Neutral	Stable	[90, 102]
**Liposomes**	Curcumin	Gradient	Sustained	Neutral	Stable	[13]
**Gold nanoparticles**	Doxorubicin	Photothermal	Pulsatile	Neutral	Stable	[90, 102]
**Polymeric micelles**	Docetaxel	Solubilization	Controlled	Neutral	Stable	[91]
**Carbon nanotubes**	Cisplatin	Diffusion	Sustained	Neutral	Unstable	[90, 102]
**Metal–organic frameworks**	Methotrexate	Degradation	Sustained	Basic	Unstable	[101]
**Polymeric nanoparticles**	Doxorubicin	Degradation	Sustained	Neutral	Stable	[90, 102]
**Liposomes**	Methotrexate	pH Gradient	Controlled	Acidic	Stable	[13]
**Dendrimers**	Paclitaxel	Swelling	Controlled	Neutral	Unstable	[99]
**Nanoemulsions**	Curcumin	Partitioning	Sustained	Neutral	Stable	[117]
**Liposomes**	Doxorubicin	pH Gradient	Sustained	Acidic	Stable	[13]
**Polymeric nanoparticles**	Paclitaxel	Diffusion	Controlled	Neutral	Stable	[90, 102]
**Dendrimers**	Methotrexate	Swelling	Pulsatile	Basic	Unstable	[99]
**Nanoemulsions**	Docetaxel	Partitioning	Pulsatile	Acidic	Stable	[117]
**Gold nanoparticles**	Curcumin	Photothermal	Triggered	Neutral	Stable	[117]
**Iron oxide nanoparticles**	Doxorubicin	Magnetic field	Sustained	Neutral	Stable	[121]
**Lipid-based**	Paclitaxel	Diffusion	Sustained	Acidic	Stable	[57]
**Polymer-lipid hybrid**	Cisplatin	Hydrolysis	Burst	Neutral	Unstable	[57]
**Carbon-based**	Cisplatin	Adsorption	Triggered	Neutral	Unstable	[90, 102]
**Gold nanorods**	Doxorubicin	Photothermal	Pulsatile	Neutral	Stable	[58]
**Silica nanoparticles**	Curcumin	pH-Responsive	Sustained	Neutral	Stable	[88]

**Fig. 6:**
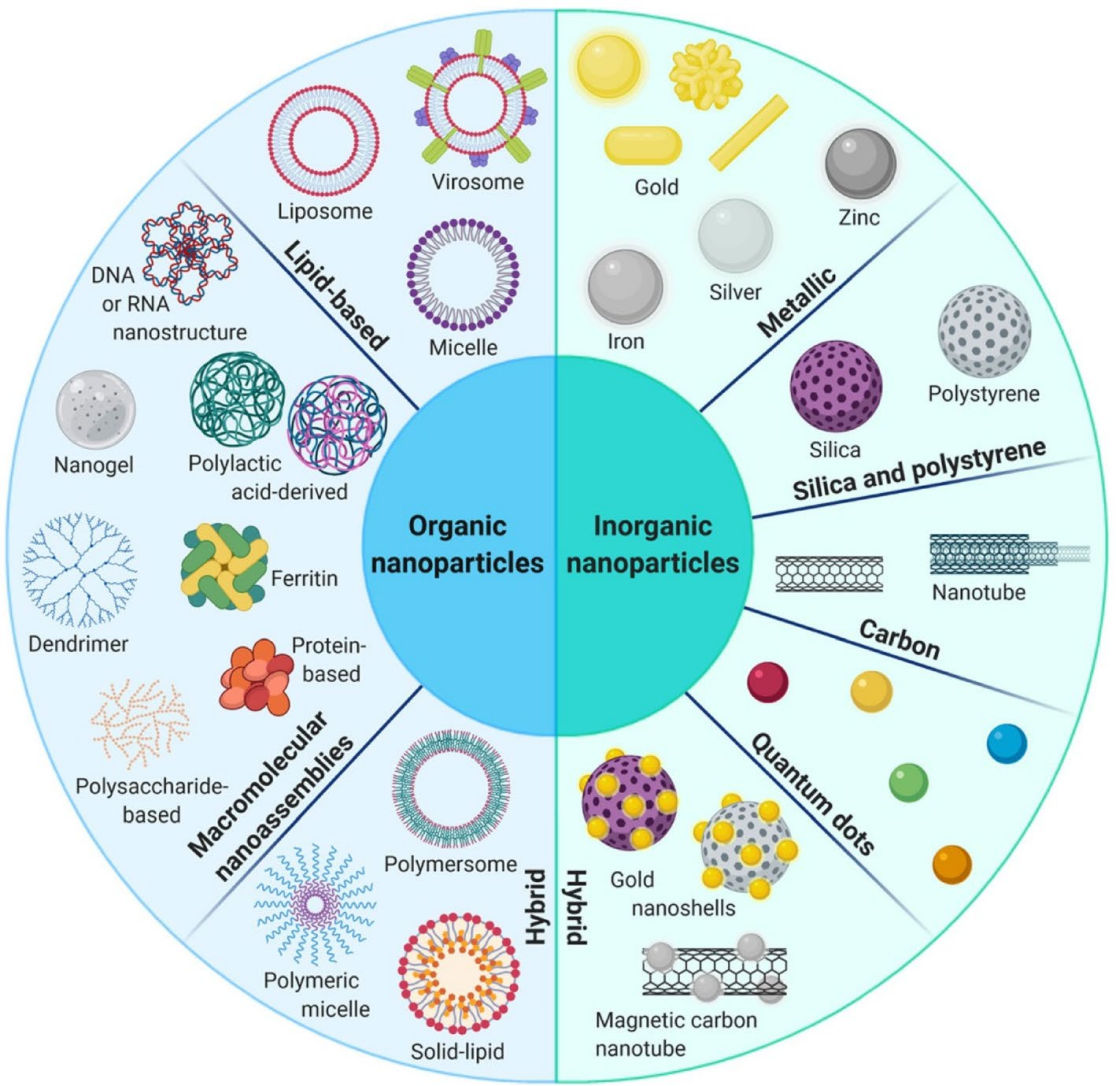
Chemically modified nanoparticles intended for use in cancer therapy. These nanoparticles can be classified as either inorganic or organic. Inorganic nanoparticles, such as metallic, silica, carbon, and quantum dots, are highly stable and possess electronical and optical properties that make them useful for cancer imaging and theragnostic. However, their solid cores may lead to the rapid degradation of conjugated therapeutic molecules in vivo. Organic nanoparticles, on the other hand, such as lipid-based and macromolecular assemblies, are less stable but have good biocompatibility and provide multiple opportunities for drug functionalization either on their surface or within their interior. Hybrid nanoparticles are a combination of both inorganic and organic nanoparticles and offer improved biocompatibility and stability. Reprint from [131] with a permission from Springer Nature

**Fig. 7:**
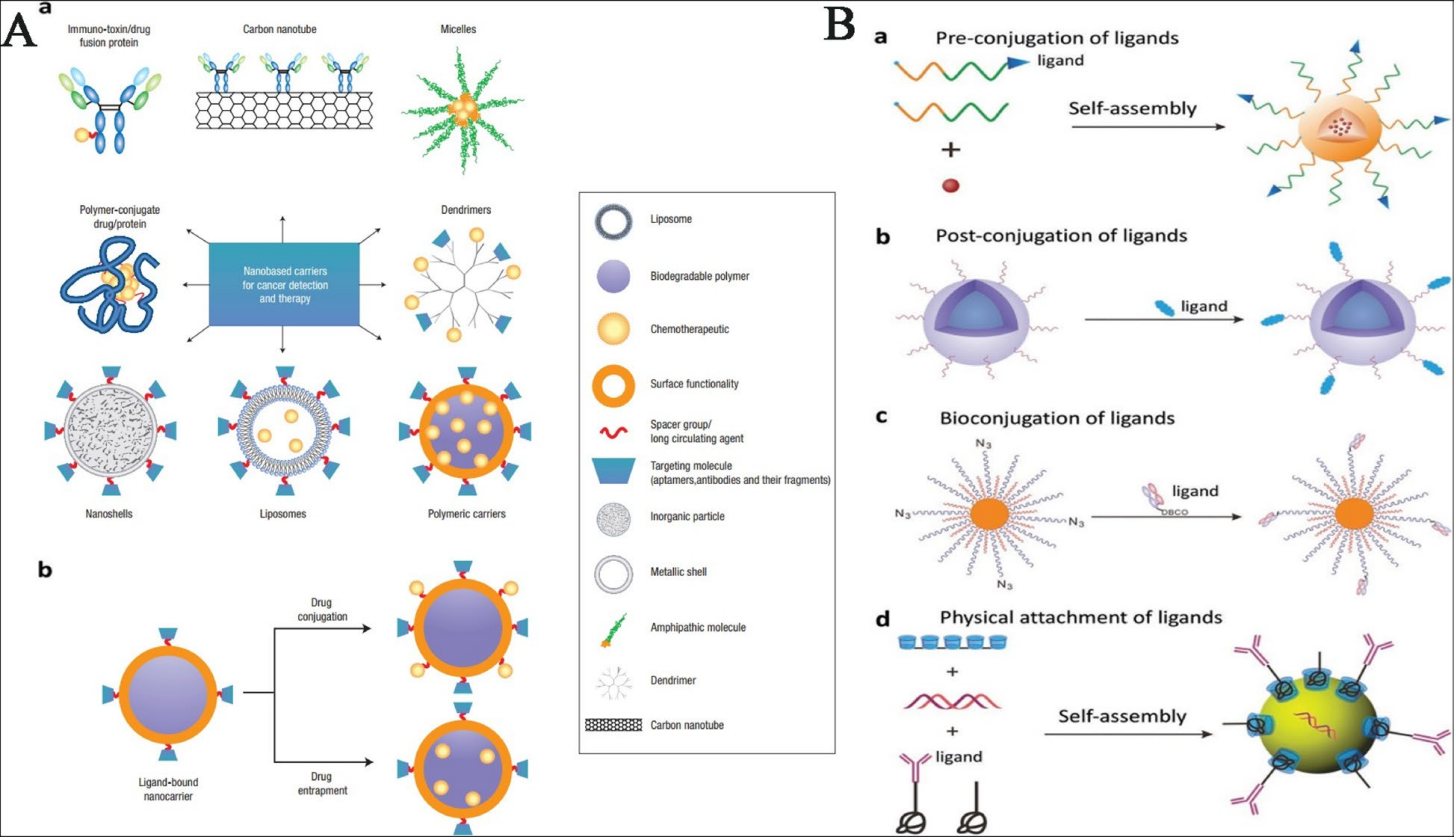
**A** Different types of nanocarriers that can be used for targeting cancer. The main components of these delivery agents usually consist of a nanocarrier, a targeting moiety that is connected to the nanocarrier, and a cargo, which can be the desired chemotherapeutic drugs. The diagram shows a range of possible delivery agents, and a schematic representation of the drug conjugation and entrapment processes. In some cases, the chemotherapeutic drugs can be bound to the nanocarrier, such as in polymer-drug conjugates, dendrimers, and some particulate carriers, while in other cases they can be trapped inside the nanocarrier. Reprint from [132] with a permission from Springer Nature. **B** The various approaches for installing targeting ligands onto nanocarriers. The strategies are divided into four categories: preconjugation (**A**), postconjugation (**B**), bioconjugation (**C**), and physical attachment (**D**). Reprint from [34] with a permission from Wiley

The nanoparticles are classified as either inorganic or organic. Inorganic nanoparticles, which include metallic, silica, carbon, and quantum dots, are highly stable and possess unique electronical and optical properties, which make them useful for cancer imaging and theranostics. However, the solid cores of inorganic nanoparticles may lead to the rapid degradation of conjugated therapeutic molecules within the body. Organic nanoparticles, such as lipid-based and macromolecular assemblies, offer good biocompatibility and provide numerous opportunities for drug functionalization on their surface or within their interior. Although organic nanoparticles are less stable than inorganic nanoparticles, they still offer several advantages in cancer therapy. Hybrid nanoparticles, which are a combination of both inorganic and organic nanoparticles, offer improved biocompatibility and stability, making them an excellent choice for cancer therapy. Figure 6 illustrates the use of chemically modified nanoparticles in cancer therapy. Figure 7-A illustrates different types of nanocarriers that can be utilized to target cancer cells. These delivery agents typically comprise of three main components: a nanocarrier, a targeting moiety, and a cargo, which may include chemotherapeutic drugs. The figure depicts various potential delivery agents, along with a schematic representation of the drug conjugation and entrapment processes. Certain nanocarriers, including polymer-drug conjugates, dendrimers, and particulate carriers, can directly bind chemotherapeutic drugs. In contrast, other nanocarriers trap the drugs within them. The diverse range of delivery agents shown in the figure offers an array of possibilities for targeted cancer therapy, highlighting the potential for nanocarrier-based drug delivery systems in the treatment of cancer. Preconjugation involves the conjugation of targeting ligands to the surface of nanocarriers before their assembly. Postconjugation, on the other hand, involves the attachment of targeting ligands to the nanocarrier surface after their formation. Bioconjugation is a strategy that uses biological recognition and binding mechanisms to attach the targeting ligands to the nanocarriers. Finally, physical attachment involves non-covalent interactions between the targeting ligands and the nanocarriers, such as electrostatic interactions and hydrophobic interactions. The selection of an appropriate strategy depends on the specific application and the properties of the nanocarriers and targeting ligands. Figure 7-B illustrates the different methods used for installing targeting ligands onto nanocarriers, which are categorized into four groups: preconjugation, postconjugation, bioconjugation, and physical attachment. Table 6 presents an overview of various nanocarriers utilized in clinical trials for cancer therapy.

**Table 6:** Clinical trials of nanocarriers for cancer therapy

**Study Title**	**Types of Cancer**	**Interventions**	**Phase**	**Outcome**	**Pateints**	**Sex**	**NCT Number**
**Paclitaxel Albumin-Stabilized Nanoparticle Formulation, Gemcitabine, and Bevacizumab in Treating Patients With Metastatic Breast Cancer**	Breast Cancer	Biological: bevacizumab, Drug: gemcitabine hydrochloride, Drug: paclitaxel albumin-stabilized nanoparticle formulation	Phase 2	6-month Progression-free Survival (PFS) Rate, Overall Survival Time, PFS Time	50	All	NCT00662129
**ABI-007 (Nab-Paclitaxel) and Gemcitabine in Treating Women With Metastatic Breast Cancer**	Breast Cancer	Drug: Gemcitabine, Drug: Paclitaxel protein-bound particles for injectable suspension (albumin-bound)	Phase 2	Proportion of Patients With Confirmed Responses, Progression-free Survival, Overall Survival, and Adverse Event	50	Female	NCT00110084
**Nanoparticle Albumin-Bound (Nab) Paclitaxel/Cyclophosphamide in Early-Stage Breast Cancer**	Breast Cancer	Drug: nab paclitaxel, Drug: Cyclophosphamide, Drug: Trastuzumab	Phase 2	Number of Participants Who Remained Alive Without Evidence of Recurrence as a Measure of Tolerability of Adjuvant Nab Paclitaxel, Disease-free Survival, Overall Survival	63	All	NCT00629499
**S0800, Nab-Paclitaxel, Doxorubicin, Cyclophosphamide, and Pegfilgrastim With or Without Bevacizumab in Treating Women With Inflammatory or Locally Advanced Breast Cancer**	Breast Cancer	Biological: bevacizumab, Biological: pegfilgrastim, Drug: cyclophosphamide, and 2 more…	Phase 2	Number of Patients With Pathological Complete Response Rate, Overall Survival, Event-free Survival, and Number of Adverse Events That Are Possibly, Probably or Definitely Related to Study Drug	215	Female	NCT00856492
**Carboplatin, Paclitaxel, and Bevacizumab in Treating Patients With Locally Recurrent or Metastatic Breast Cancer**	Breast Cancer	Biological: bevacizumab, Drug: Carboplatin, Drug: ABI-007	Phase 2	Progression-free Survival, Response Rate at End of Treatment, Overall Survival	32	All	NCT00654836
**Carboplatin and Paclitaxel Albumin-Stabilized Nanoparticle Formulation Followed by Radiation Therapy and Erlotinib in Treating Patients With Stage III Non-Small Cell Lung Cancer That Cannot Be Removed By Surgery**	Lung Cancer	Drug: carboplatin, Drug: erlotinib hydrochloride, Drug: paclitaxel albumin-stabilized nanoparticle formulation, Radiation: radiation therapy	Phase 2	Overall Survival at 12 Months, Response Rate, Progression-free Survival	78	All	NCT00553462
**Paclitaxel Albumin-Stabilized Nanoparticle Formulation and Carboplatin in Treating Patients With Stage IIIB, Stage IV, or Recurrent Non-Small Cell Lung Cancer**	Lung Cancer	Drug: carboplatin, Drug: paclitaxel albumin-stabilized nanoparticle formulation, Genetic: protein expression analysis, and 3 more…	Phase 2	Overall Response Rate Defined as Complete or Partial Response as Assessed by RECIST Version 1.0 Criteria, Progression Free Survival, Overall Survival, and Incidence and Intensity of Adverse Events Graded According to NCI CTCAE v. 3.0	63	All	NCT00729612
**Phase II NCT (Neoadjuvant Chemotherapy) w/ Weekly Abraxane in Combination With Carboplatin & Bevacizumab in Breast Cancer**	Breast Cancer	Drug: bevacizumab, Drug: carboplatin, Drug: nab-paclitaxel, and 2 more…	Phase 2	Number of Patients With Pathologic Complete Response (pCR), Side Effects of Weekly Nab-paclitaxel, Carboplatin and Bevacizumab, Evaluation of Dynamic Contrast-enhanced Magnetic Resonance Imaging in Assessing pCR at Baseline and After 2 Cycles of Neoadjuvant Therapy, and Overall Expression of LZTS1 Before and After Neoadjuvant Therapy as Assessed by Immunohistochemistry	33	Female	NCT00675259
**Paclitaxel Albumin-Stabilized Nanoparticle Formulation in Treating Patients With Previously Treated Advanced Non-small Cell Lung Cancer**	Recurrent Non-Small Cell Lung Carcinoma, Stage IV Non-Small Cell Lung Cancer	Other: Laboratory Biomarker Analysis, Drug: Paclitaxel Albumin-Stabilized Nanoparticle Formulation	Phase 2	Overall Response Rate (Complete and Partial Response) Defined by RECIST 1.1 Criteria, Overall Percentage of Patients Experiencing Toxicity Within a Clinically Significant Category Defined as Neutropenia, Neutropenic Fever, or Neuropathy, Overall Survival	26	All	NCT01620190
**Abraxane Therapy in Patients With Pancreatic Cancer Who Failed First-Line Gemcitabine Therapy**	Pancreatic Cancer	Drug: Abraxane	Phase 2	Overall Survival Rate at 6 Months, Number of Participants Showing Complete or Partial Response, Number of Participants Showing Stable Disease	20	All	NCT00691054
**Phase 1/2 Study of ABI-009 in Nonmuscle Invasive Bladder Cancer**	Non-muscle Invasive Bladder Cancer (NMIBC)	Drug: ABI-009, Drug: Gemcitabine	Phase 1, Phase 2	Phase 1: Dose Limiting Toxicities (DLT) Following Intravesical Administration of ABI-009, Phase 2: Number of Participants Achieving a Complete Response Following Intravesical Administration of ABI-009 and Gemcitabine, Phase 1: Number of Participants Achieving a Complete Response Following Intravesical Administration of ABI-009	21	All	NCT02009332
**Doxorubicin Hydrochloride, Cyclophosphamide, and Filgrastim Followed By Paclitaxel Albumin-Stabilized Nanoparticle Formulation With or Without Trastuzumab in Treating Patients With Breast Cancer Previously Treated With Surgery**	Estrogen Receptor-positive Breast Cancer, HER2-positive Breast Cancer, and 6 more…	Drug: doxorubicin hydrochloride, Drug: cyclophosphamide, Biological: filgrastim, and 4 more…	Phase 2	Disease-free Survival Following a Dose-intensive Weekly Regimen of Adriamycin + Oral Cyclophosphamide Augmented With G-CSF Support Followed by Abraxane and Herceptin, Delivered Dose Intensity of the Regimen, Toxicity Associated With This Regimen	60	Female	NCT00407888
**Phase II Study With Abraxane, Bevacizumab and Carboplatin in Triple Negative Metastatic Breast Cancer**	Breast Cancer	Drug: Abraxane, Drug: Bevacizumab, Drug: Carboplatin	Phase 2	Best Clinical Response Expressed as Percentage of Participants Treated With Combination Regimen of Weekly Abraxane® and Carboplatin Plus Biweekly Bevacizumab to Treat Women With Stage IV or Inoperable Stage III "Triple Negative" Metastatic Breast Cancer, Median Proportion Progression-free as Estimated by Kaplan–Meier Methods, To Evaluate Sequential Plasma Samples for Presence of Selected Angiogenic Markers	41	Female	NCT00479674
**Nab-Paclitaxel and Bevacizumab Followed By Bevacizumab and Erlotinib in Metastatic Breast Cancer**	Estrogen Receptor-negative Breast Cancer, HER2-negative Breast Cancer, and 3 more…	Drug: paclitaxel albumin-stabilized nanoparticle formulation, Biological: bevacizumab, Drug: erlotinib hydrochloride	Phase 2	Progression-free Survival (PFS), Overall Survival, Percentage of Participants With Response	59	Female	NCT00733408
**Sargramostim and Paclitaxel Albumin-Stabilized Nanoparticle Formulation in Treating Patients With Advanced Ovarian Cancer, Fallopian Tube Cancer, or Primary Peritoneal Cancer That Did Not Respond to Previous Chemotherapy**	Brenner Tumor, Fallopian Tube Cancer, Ovarian Clear Cell Cystadenocarcinoma, and 9 more…	Biological: sargramostim, Drug: paclitaxel albumin-stabilized nanoparticle formulation, Other: laboratory biomarker analysis, and 2 more…	Phase 2	Time to Progression, Response Rate, Correlation Between Circulating Monocytes and Time to Progression	21	All	NCT00466960
**Paclitaxel Albumin-Stabilized Nanoparticle Formulation and Gemcitabine Hydrochloride With or Without WEE1 Inhibitor AZD1775 in Treating Patients With Previously Untreated Pancreatic Cancer That Is Metastatic or Cannot Be Removed by Surgery**	Metastatic Pancreatic Adenocarcinoma, Stage III Pancreatic Cancer AJCC v6 and v7, Stage IV Pancreatic Cancer AJCC v6 and v7, and 1 more…	Drug: AZD1775, Drug: Gemcitabine, Drug: Nab-paclitaxel	Phase 1, Phase 2	Number of Participants With Dose Limiting Toxicities (DLT), To Determine the Pharmacokinetics of AZD1775 in Combination With Nab-paclitaxel and Gemcitabine, Progression-free Survival	8	All	NCT02194829
**Paclitaxel, Nab-paclitaxel, or Ixabepilone With or Without Bevacizumab in Treating Patients With Stage IIIC or Stage IV Breast Cancer**	Estrogen Receptor Negative, Estrogen Receptor Positive, HER2/Neu Negative, and 6 more…	Biological: Bevacizumab, Drug: Ixabepilone, Other: Laboratory Biomarker Analysis, and 3 more…	Phase 3	Progression Free Survival, Objective Tumor Response Rate, Time to Treatment Failure	799	All	NCT00785291
**Paclitaxel Albumin-Stabilized Nanoparticle Formulation in Treating Patients With Recurrent or Persistent Ovarian Epithelial Cancer, Fallopian Tube Cancer, or Primary Peritoneal Cancer**	Fallopian Tube Carcinoma, Primary Peritoneal Carcinoma, Recurrent Ovarian Carcinoma	Drug: Paclitaxel Albumin-Stabilized Nanoparticle Formulation	Phase 2	Tumor Response, Frequency and Severity of Observed Adverse Effects, Progression-free Survival, Overall Survival	51	Female	NCT00499252
**Study of Albumin-bound Paclitaxel (Abraxane) in Combination With Carboplatin and Herceptin in Patients With Advanced Breast Cancer**	Breast Cancer	Drug: Albumin-bound paclitaxel, Drug: Carboplatin, Drug: Herceptin®	Phase 2	Percentage of Participants Who Achieved an Objective Confirmed Complete or Partial Overall Response, Percentage of Participants With a Total Response, Time to Disease Progression	32	Female	NCT00093145
**A Phase I/II Clinical Trial of Vidaza With Abraxane in Patients With Advanced/Metastatic Solid Tumors and Breast Cancer**	Advanced or Metastatic Solid Tumors, Advanced or Metastatic Breast Cancer	Drug: Azacitidine (Vidaza), Drug: Nab-paclitaxel (Abraxane)	Phase 1, Phase 2	Phase I: Percentage of Participants Responding to Treatment, Phase II: Percentage of Participants With Objective Response Rate (ORR) Measured Using RECIST 1.0 Criteria, Number of Participants With ER + Status, Progression-free Survival	30	All	NCT00748553
**A Phase 2 Study of CRLX101(NLG207) in Patients With Advanced Non-Small Cell Lung Cancer**	Non-Small Cell Lung Cancer	Drug: CRLX101, Other: Best Supportive Care	Phase 2	To Compare Overall Survival of Patients Treated With CRLX101 + BSC to Those Patients Treated With BSC Only, Assess Objective Response Rate (ORR) of CRLX101 + BSC Compared to BSC Only	157	All	NCT01380769
**Nab-Paclitaxel, Cisplatin, and Cetuximab With Concurrent Radiation Therapy for Locally Advanced Head and Neck Cancer**	Head and Neck Cancer	Biological: Cetuximab, Drug: Cisplatin, Drug: Nab-Paclitaxel, Radiation: intensity-modulated radiation therapy	Phase 1, Phase 2	Phase I Maximum Tolerated Dose of Nab-Paclitaxel, Phase II 2-year Progression-free Survival, Phase II 2-year Local Control, Phase II 2-year Overall Survival	37	All	NCT00851877
**Pre-Operative Staging of Pancreatic Cancer Using Superparamagnetic Iron Oxide Magnetic Resonance Imaging (SPIO MRI)**	Pancreatic Cancer	Drug: Superparamagnetic Iron Oxide Magnetic Resonance Imaging	Phase 4	To Determine the Sensitivity High Resolution Magnetic Resonance Imaging With Lymphotrophic Superparamagnetic Nanoparticles to Identify Small and Otherwise Undetectable Lymph Node Metastases., To Determine the Specificity of High Resolution Magnetic Resonance Imaging With Lymphotrophic Superparamagnetic Nanoparticles to Identify Small and Otherwise Undetectable Lymph Node Metastases	35	All	NCT00920023
**Gemcitabine Hydrochloride, Cisplatin, and Nab-Paclitaxel in Treating Patients With Advanced or Metastatic Biliary Cancers**	Stage III Intrahepatic Cholangiocarcinoma AJCC v7, Stage IIIA Gallbladder Cancer AJCC v7, Stage IIIB Gallbladder Cancer AJCC v7, and 6 more…	Drug: Cisplatin, Drug: Gemcitabine Hydrochloride, Other: Laboratory Biomarker Analysis, Drug: Nab-paclitaxel	Phase 2	Median Progression Free Survival (PFS), Median Overall Survival (OS), Number of Participants With Treatment Response Rate	62	All	NCT02392637
**Phase II Lapatinib Plus Nab-Paclitaxel As First And Second Line Therapy In her2 + MBC**	Neoplasms, Breast	Drug: Lapatinib/nab-Paclitaxel	Phase 2	Overall Tumor Response (OR), Overall Survival (OS), Duration of Response (DOR)	60	Female	NCT00709761
**A Phase III Study of NK105 in Patients With Breast Cancer**	Breast Cancer Nos Metastatic Recurrent	Drug: NK105, Drug: Paclitaxel	Phase 3	Progression Free Survival, Overall Survival, Overall Response Rate,	436	Female	NCT01644890
**S1505: Combination Chemotherapy or Gemcitabine Hydrochloride and Paclitaxel Albumin-Stabilized Nanoparticle Formulation Before Surgery in Treating Patients With Pancreatic Cancer That Can Be Removed by Surgery**	Pancreatic Adenocarcinoma, Resectable Pancreatic Carcinoma	Drug: Fluorouracil, Drug: Gemcitabine Hydrochloride, Drug: Irinotecan Hydrochloride, and 3 more…	Phase 2	Overall Survival (OS), Number of Patients With Grade 3 Through Grade 5 Adverse Events That Are Related to Study Drug, Number of Patients Going to Surgery for Resection After Preoperative Chemotherapy	147	All	NCT02562716
**Induction Chemotherapy With ACF Followed by Chemoradiation Therapy for Adv. Head & Neck Cancer**	Head and Neck Neoplasms	Drug: paclitaxel albumin-stabilized nanoparticle formulation, Drug: Cisplatin, Drug: Fluorouracil, and 3 more…	Phase 2	Percentage of Participants With Complete Response (CR) by Clinical Exam at Primary Tumor Site, Percentage of Participants With Partial Response (PR) at Primary Tumor Site, Number of Participants Per Anatomic Tumor Response by CT Scan	30	All	NCT01566435
**Topical Imiquimod and Abraxane in Treating Patients With Advanced Breast Cancer**	Male Breast Cancer, Recurrent Breast Cancer, Skin Metastases, and 1 more…	Drug: imiquimod, Drug: Abraxane, Other: laboratory biomarker analysis, and 2 more…	Phase 2	Anti-tumor Effects of Imiquimod as Assessed by Modified World Health Organization (WHO) Criteria, Safety and Systemic Toxicity as Assessed by a Review of Medical History, Physical Exam, Systems, Performance Status, and Clinical Labs (CBC and CMP), Pathologic Response by Immunohistochemical (IHC)as Assessed by Skin Punch Biopsy of the Target Lesion	15	All	NCT00821964
**Phase 1 Trial of PAN-301–1 (SNS-301) in Cancer Patients**	Prostate Cancer	Biological: PAN-301–1	Phase 1	Safety Assessed by Development of Adverse Events and Dose-limiting Toxicity to Determine Maximum Tolerated Dose, Safety Assessed by Administration Site Reactions, Abnormal Laboratory Values and/or Clinically Significant Changes in Physical Examinations	18	All	NCT00262916

The use of nanocarriers in drug delivery has revolutionized the field of medicine by providing targeted and controlled release of therapeutic agents. This critical analysis aims to shed light on various approved nanocarriers, evaluating their effectiveness, limitations, and potential side effects based on clinical trial data [133]. Understanding the practical implications of these formulations is crucial for optimizing drug delivery strategies [134]. Lipid nanoparticles, including liposomes and lipid-based nanocarriers, have gained widespread acceptance due to their biocompatibility and versatility. They effectively encapsulate hydrophobic drugs and improve their solubility, enhancing drug bioavailability [135]. However, their stability can be a concern, leading to premature drug release [98]. Clinical trials have reported minor side effects, such as infusion-related reactions, but overall, lipid nanoparticles have demonstrated remarkable potential in delivering a range of therapeutics [136]. Polymeric micelles, formed from amphiphilic block copolymers, offer promising drug delivery platforms. They enhance the solubility of poorly water-soluble drugs, improving their bioavailability [137]. However, their stability and drug-loading capacity can be limiting factors, and clinical trials have reported challenges in maintaining therapeutic concentrations [138]. Additionally, the long-term safety profile of some polymers remains under investigation [100]. Gold nanoparticles and gold nanorods have shown promise in photothermal therapy and imaging applications. While they offer precise control over drug release through external stimuli, their clinical utility has been limited due to concerns about toxicity [139]. Clinical trials have raised questions about the long-term impact of gold nanoparticles on the body and the potential for immune responses [140]. Mesoporous silica nanoparticles offer a unique drug delivery platform with high drug-loading capacity and tunable release kinetics [23]. However, their relatively large size may limit their ability to target specific tissues or cells. Clinical trials have provided valuable insights into their safety and have identified potential side effects, such as gastrointestinal disturbances [141]. Albumin-bound nanoparticles have been developed to improve the delivery of hydrophobic drugs. They enhance drug stability and can accumulate in tumor tissues through the enhanced permeability and retention effect [142]. Clinical trials have reported relatively few adverse effects, but there is ongoing research to optimize their efficacy [143]. PLGA nanoparticles have been extensively studied for their controlled drug release capabilities. They offer biodegradability and can be tailored for various drug types [89]. However, their effectiveness can be compromised by rapid drug release or poor drug loading. Clinical trials have identified potential challenges in achieving consistent therapeutic outcomes [144]. Carbon nanotubes have shown promise in drug delivery, but concerns regarding their biocompatibility and toxicity have limited their clinical translation [145]. Clinical trials have revealed safety concerns, particularly in long-term exposure scenarios [146]. Iron oxide nanoparticles, including magnetic nanoparticles, have been explored for targeted drug delivery and imaging [147]. While they offer precise control over drug release, there are concerns about their potential toxicity, especially with long-term use. Clinical trials have highlighted the need for comprehensive safety assessments [148]. Quantum dots have unique optical properties, making them valuable for imaging and diagnostics [149]. However, concerns about their potential toxicity, particularly due to heavy metal components, have raised questions about their clinical use. Lipid-polymer hybrid nanoparticles represent a promising approach by combining the advantages of both lipid and polymeric nanocarriers [150]. They offer improved stability and drug-loading capacity, making them suitable for a wide range of drugs. However, clinical trials are needed to assess their long-term safety and potential side effects. Calcium phosphate nanoparticles have shown potential in gene delivery and vaccine formulations. They offer biocompatibility and controlled release properties. However, clinical trials have highlighted challenges in achieving efficient transfection and potential immunogenicity concerns [151]. Liposome-encapsulated nanoparticles combine the advantages of liposomes and nanoparticles, enhancing drug delivery efficiency [152]. Clinical trials have reported favorable safety profiles, but their efficacy in specific therapeutic applications may vary [153]. Calcium phosphate-coated iron oxide nanoparticles provide a versatile platform for imaging and drug delivery. However, their clinical translation may be hindered by concerns about long-term toxicity and potential side effects [154]. Self-assembling peptide nanofibers offer a unique approach for drug delivery and tissue engineering. Clinical trials have demonstrated their biocompatibility, but further research is needed to assess their long-term effects [155]. Lipid-polymer-metal hybrid nanoparticles combine the properties of lipids, polymers, and metals for multifunctional drug delivery systems. Clinical trials are essential to evaluate their safety and effectiveness in complex therapeutic applications [156]. Gold nanoparticles have found applications in drug delivery, imaging, and therapy. Clinical trials have identified potential toxicity issues, especially with larger particles, emphasizing the importance of rigorous safety assessments [157]. Magnetic nanoparticles have shown potential in drug targeting and imaging. Clinical trials have reported some concerns about their potential impact on the immune system and long-term biocompatibility [158]. Silica nanoparticles have been explored for their drug delivery capabilities. Clinical trials have reported some safety concerns related to their size and surface properties, highlighting the need for careful design and optimization [159]. Polymeric nanoparticles with surface modification offer tailored drug delivery solutions. Clinical trials have revealed promising outcomes in specific applications, but their safety and efficacy may vary depending on the modification and drug being delivered [160]. Graphene oxide nanoparticles and carbon quantum dots have shown potential in drug delivery and imaging. However, concerns about their biocompatibility and potential toxicity have limited their clinical adoption [161]. Nanogels represent a versatile platform for drug delivery, with tunable properties. Clinical trials are necessary to assess their safety and effectiveness in different therapeutic contexts [162, 163]. Cationic liposomes and chitosan nanoparticles offer unique advantages for gene and nucleic acid delivery. Clinical trials have demonstrated their safety and efficacy, but further optimization is needed for broader clinical applications [164]. Dendrimer-encapsulated nanoparticles and micelle-encapsulated nanoparticles provide controlled drug release capabilities. Clinical trials are essential to evaluate their safety and effectiveness in specific drug delivery scenarios [165].

## Various nano-formulations: revolutionizing drug delivery

Nanotechnology has ushered in a new era in science and medicine, bringing forth innovative solutions to longstanding challenges across various domains, with drug delivery standing out prominently [166]. Through the precise manipulation of materials at the nanoscale, nanotechnology has sparked considerable interest for its potential to revolutionize drug delivery. Nano-formulations hold the promise of significantly improving the bioavailability, efficacy, and safety of drugs [59]. By exploiting the unique properties of nanoparticles, such as their high surface area and tunable characteristics, scientists and researchers have developed novel drug delivery systems that can target specific cells or tissues, reduce side effects, and enhance therapeutic outcomes. This breakthrough in nanotechnology not only opens new avenues for personalized medicine but also holds the potential to transform the way we approach healthcare and treatment strategies in the future [20].

### Polymeric nanoparticles

**Fig. 8:**
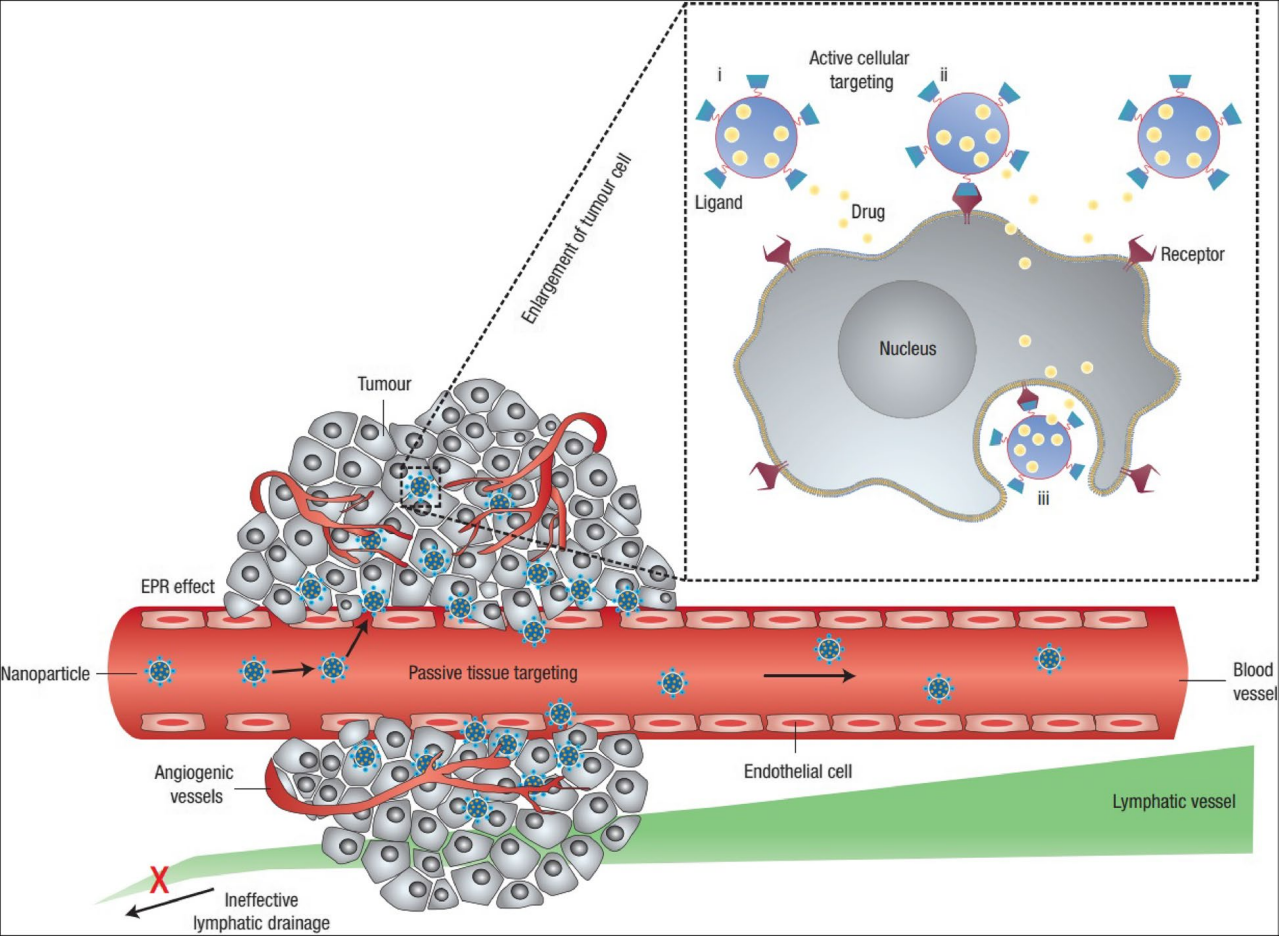
Various ways in which nanocarriers can transport drugs to tumors, using polymeric nanoparticles as a representative example. To achieve passive tissue targeting, the nanoparticles extravasate through the tumor vasculature due to increased permeability and inefficient lymphatic drainage (ePr effect). Active cellular targeting can be accomplished by modifying the surface of the nanoparticles with ligands that promote recognition and binding to specific cells. Nanoparticles can then either (i) release their contents in close proximity to the target cells; (ii) adhere to the cell membrane and serve as an extracellular sustained-release drug reservoir; or (iii) become internalized by the cell. Reprint from [132] with a permission from Springer Nature

The term "nanoparticle" is used to describe any particle with a size on the nanometer scale. Metal nanoparticles, polymeric nanoparticles (PNPs), monoclonal antibody nanoparticles (mAbNPs), extracellular vesicles (EVs), and PNPs have all been the subject of much study (NPs). PNPs are colloidal macromolecules between ten and one thousand nanometers in size [57, 58]. PNPs serve as drug transporters, delivering chemotherapy chemicals directly to tumor locations before gradually releasing them. When medications are enclosed inside nanoparticles or connected to the surfaces of nanoparticles, a nanocapsule or nanosphere is formed [48]. Nanoparticle building blocks have experienced multiple evolutions throughout time. Early efforts to create nanoparticles relied on the use of nonbiodegradable polymers such as polymethyl methacrylate (PMMA), polyacrylamide, polystyrene, and polyacrylates [167]. To reduce toxicity and prolong inflammation, it is important to eliminate any polymeric nanoparticles generated from these substances as soon as feasible [88]. The difficulties in degrading, excreting, or physically removing these polymer-based nanoparticles from tissues, which had previously posed a health risk, have been addressed [84]. These nanoparticles accumulated because they were so difficult to remove. Because of advances in biodegradable polymer manufacture, toxicity has been decreased, while drug release kinetic patterns have been enhanced, and biocompatibility has been widened [168]. Table 7 outlines the biocompatibility of various nanocarrier materials, which play a crucial role in drug delivery systems. Figure 8 depicts the various mechanisms by which nanocarriers can deliver drugs to tumours.

**Table 7:** Biocompatibility of nanocarrier materials

**Nanocarrier Material**	**Biocompatibility Score**	**Cytotoxicity**	**Immunogenicity**	**Hemocompatibility**	**Clearance Pathway**	**Description**	**Novelty**	**Advantages**	**References**
**Liposomes**	High	Low	Low	Low	RES	Phospholipid bilayer vesicles with aqueous core	First generation nanocarriers	Biodegradable, versatile, well-established	[13]
**Polymeric Nanoparticles**	Medium to High	Variable	Variable	Variable	RES, Renal, or Lymphatic	Nanoparticles composed of synthetic or natural polymers	Tailorable size and surface properties, good drug payload capacity	Potential toxicity, batch-to-batch variability	[90, 102]
**Gold Nanoparticles**	Low to Medium	Low to Moderate	Low	Low to Moderate	RES or Renal	Nanoparticles made of gold with surface coatings	Good biocompatibility, versatile	Potential for accumulation in organs, poor biodegradability	[58]
**Carbon Nanotubes**	Low to Medium	Variable	Variable	Variable	RES, Pulmonary, or Renal	Cylindrical carbon structures with high aspect ratio	High drug payload capacity, tunable properties	Potential toxicity, poor biodegradability	[169]
**Magnetic Nanoparticles**	Low to Medium	Variable	Variable	Variable	RES or Renal	Nanoparticles with magnetic properties	Good targeting ability, potential for imaging	Potential for accumulation in organs, poor biodegradability	[59]
**Calcium Phosphate Nanoparticles**	High	Low	Low	Low	RES or Renal	Nanoparticles composed of calcium and phosphate	Good biocompatibility, potential for bone-targeted therapy	Limited drug payload capacity, potential for precipitation	[124, 125]
**Protein-Based Nanocarriers**	High	Low	Low	Low	RES or Renal	Nanocarriers composed of proteins	High biocompatibility, potential for targeted therapy	Limited stability, high production cost	[170]
**DNA Nanocarriers**	Medium	Low	Low	Low	Renal or Hepatic	Nanocarriers made of DNA or DNA-based materials	Potential for gene therapy, good biodegradability	Limited drug payload capacity, potential for immunogenicity	[171]
**Lipid Nanoparticles**	High	Low	Low	Low	RES or Renal	Nanoparticles composed of lipids	Versatile, easy to produce, good stability	Limited drug payload capacity, potential for lipid accumulation	[99]
**Iron Oxide Nanoparticles**	Low to Medium	Low	Low	Low to Moderate	RES or Renal	Nanoparticles with magnetic properties	Good targeting ability, potential for imaging	Potential for accumulation in organs, poor biodegradability	[121]
**Silica Nanoparticles**	Low to Medium	Variable	Variable	Variable	RES or Renal	Nanoparticles composed of silica	High drug payload capacity, good stability	Potential for toxicity, poor biodegradability	[88]
**Polyethylene Glycol Nanoparticles**	High	Low	Low	Low	Renal or Hepatic	Nanoparticles with PEG coatings	Good biocompatibility, long circulation time	Potential for instability, potential for immune response	[172]
**Albumin Nanoparticles**	High	Low	Low	Low	RES or Renal	Nanoparticles composed of albumin	Good biocompatibility, potential for targeted therapy	Limited drug payload capacity, potential for instability	[27]
**Polysaccharide Nanoparticles**	High	Low	Low	Low	RES or Renal	Nanoparticles composed of polysaccharides	Good biocompatibility, potential for targeted therapy	Limited drug payload capacity, potential for batch-to-batch variability	[173]
**Graphene Oxide Nanoparticles**	Low to Medium	Variable	Variable	Variable	RES, Pulmonary, or Renal	Nanoparticles composed of graphene oxide	High drug payload capacity, good stability	Potential for toxicity, poor biodegradability	[174]
**Chitosan Nanoparticles**	High	Low	Low to Moderate	Low	Renal or Hepatic	Nanoparticles composed of chitosan	Good biocompatibility, potential for targeted therapy	Potential for aggregation, limited drug payload capacity	[84]
**Quantum Dots**	Low to Medium	Low to Moderate	Low to Moderate	Low to Moderate	RES or Renal	Nanoparticles made of semiconducting materials	Good imaging properties, potential for targeted therapy	Potential for toxicity, poor biodegradability	[21, 103, 104]
**DNA Origami Nanoparticles**	Medium	Low	Low	Low	Renal or Hepatic	Nanocarriers made of DNA and folded into specific shapes	Potential for targeted therapy and gene delivery	Limited stability, potential for immunogenicity	[175]
**Metal–Organic Frameworks**	Medium to High	Variable	Variable	Variable	RES or Renal	Nanocarriers composed of metal ions and organic ligands	Tailorable properties, potential for drug delivery and imaging	Potential for toxicity, limited biodegradability	[101]
**Self-Assembling Peptides**	High	Low	Low	Low	RES or Renal	Peptides that self-assemble into nanocarriers	Good biocompatibility, potential for targeted therapy	Limited drug payload capacity, potential for instability	[87]
**Lipid-Polymer Hybrid Nanoparticles**	High	Low	Low	Low	RES or Renal	Nanoparticles composed of lipids and polymers	Tailorable size and surface properties, good drug payload capacity	Potential for instability, potential for lipid accumulation	[57]
**Carbon Quantum Dots**	Low to Medium	Low	Low	Low	RES or Renal	Nanoparticles composed of carbon materials	Good imaging properties, potential for drug delivery	Potential for toxicity, poor biodegradability	[176]

**Fig. 9:**
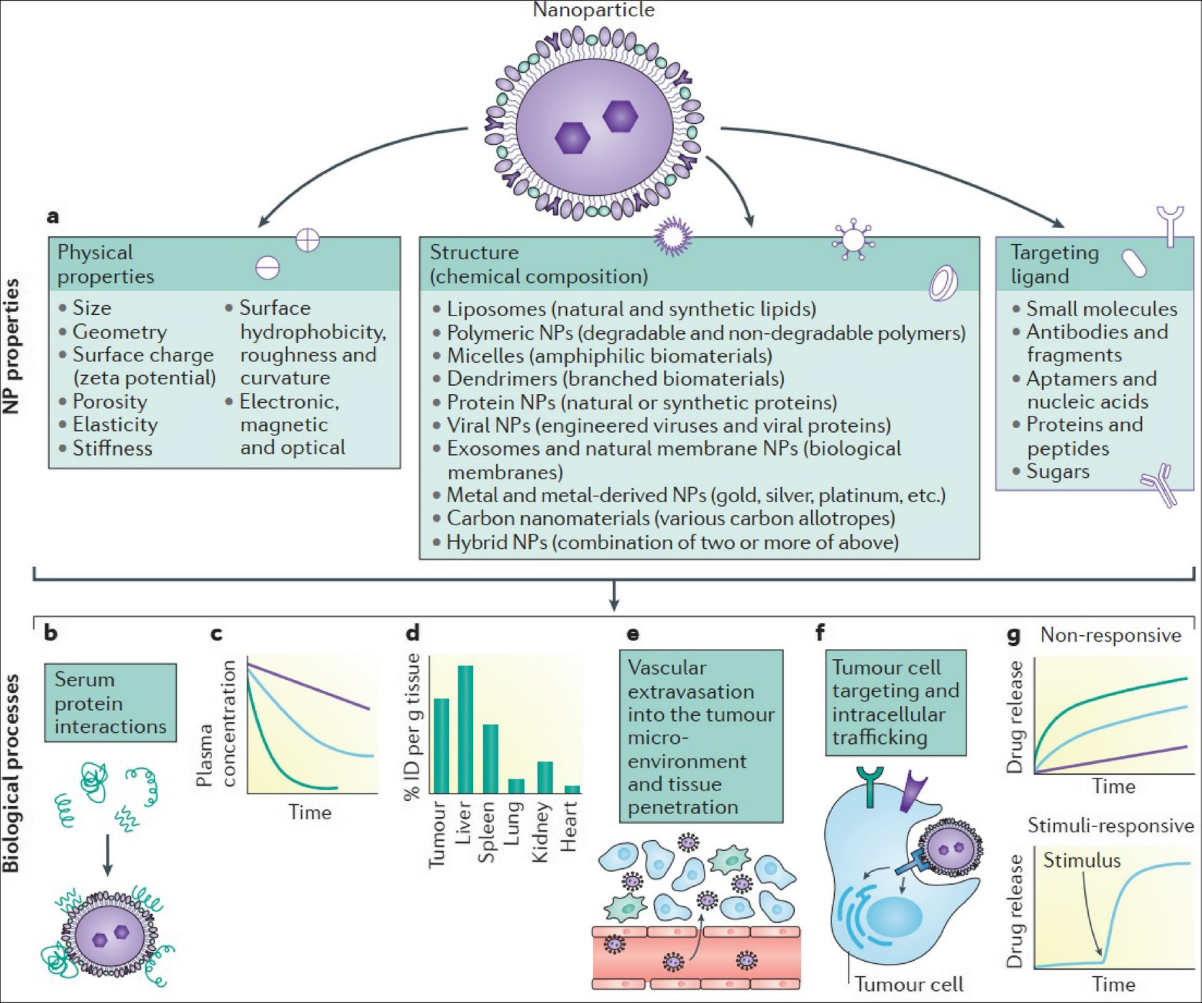
The characteristics of nanoparticles impact their ability to be delivered systemically to tumors. Nanoparticles are composed of various materials and possess different physical and chemical attributes, such as size, shape, surface properties, and flexibility, and can be modified with diverse ligands to target tumors. These properties influence the biological mechanisms involved in delivering nanoparticles to tumors, including interactions with serum proteins, circulation in the bloodstream, distribution throughout the body, penetration through the tumor's blood vessels and tissues, targeting of tumor cells, and intracellular movement. Additionally, nanoparticles can be engineered to control the release of their contents. Reprint from [177] with a permission from Springer Nature

The diagram shows polymeric nanoparticles represented as circles that can passively target the tumour tissue by extravasating through the tumour vasculature and ineffective lymphatic drainage (ePr effect). In addition to passive targeting, the diagram illustrates active cellular targeting by functionalizing the surface of the nanoparticles with ligands that promote cell-specific recognition and binding. The inset in the diagram shows that the nanoparticles can release their contents in close proximity to the target cells, attach to the cell membrane and act as an extracellular sustained-release drug depot, or internalize into the cell. Natural polymers, including chitosan, alginate, gelatin, and albumin, as well as synthetic polymers like polylactic acid (PLA), poly(lactic-co-glycolic acid), poly(amino acids), and poly(-caprolactone) (PCL), are all examples of such polymers [168]. These recently produced polymeric nanoparticles provide special advantages according to their architecture and characteristics. PNPs are a practical method for making unstable pharmaceuticals more stable [20]. Chemical medicines may be given orally or intravenously with PNPs, and they have a higher loading capacity than free pharmaceuticals. It has been shown that adding dexamethasone or tocopheryl succinate to cisplatin-loaded PNPs may mitigate the ototoxicity that results from cisplatin usage in chemotherapy [20]. This property protects drugs against degradation, which lessens the chance of their adverse effects on non-target tissues. Medication distribution, for instance, often employs one of two methods: active targeting or passive targeting [44]. Overactive angiogenesis gives an advantage that is objectively known as EPR when there is a robust extracellular matrix present, making it more difficult for drugs to reach the tissue [125]. Growing tumors have high energy and oxygen needs. Meanwhile, tumor-induced angiogenesis generates many immature vasculatures, which obstruct lymphatic drainage [13]. This leakage in the blood vessel wall makes it possible for chemical drugs to reach tumors. Figure 9 highlights the importance of the characteristics of nanoparticles in their ability to be delivered systemically to tumors. These nanoparticles are made up of different materials and possess unique physical and chemical attributes, such as size, shape, surface properties, and flexibility. Furthermore, these nanoparticles can be customized with a variety of ligands to target specific tumors. The diverse properties of these nanoparticles influence the biological mechanisms involved in their delivery to tumors, such as their interactions with serum proteins, their distribution throughout the body, their penetration through the tumor's blood vessels and tissues, their targeting of tumor cells, and their intracellular movement. Moreover, the nanoparticles can be engineered to control the release of their contents, enhancing their efficacy in treating cancer. However, the particle size of the drug is crucial since regular particles cannot enter malignant cells unless they are very small. However, because of impaired lymphatic drainage, nanoparticles and their associated chemical medication carriers may rapidly permeate targeted areas and concentrate there [105].

**Fig. 10:**
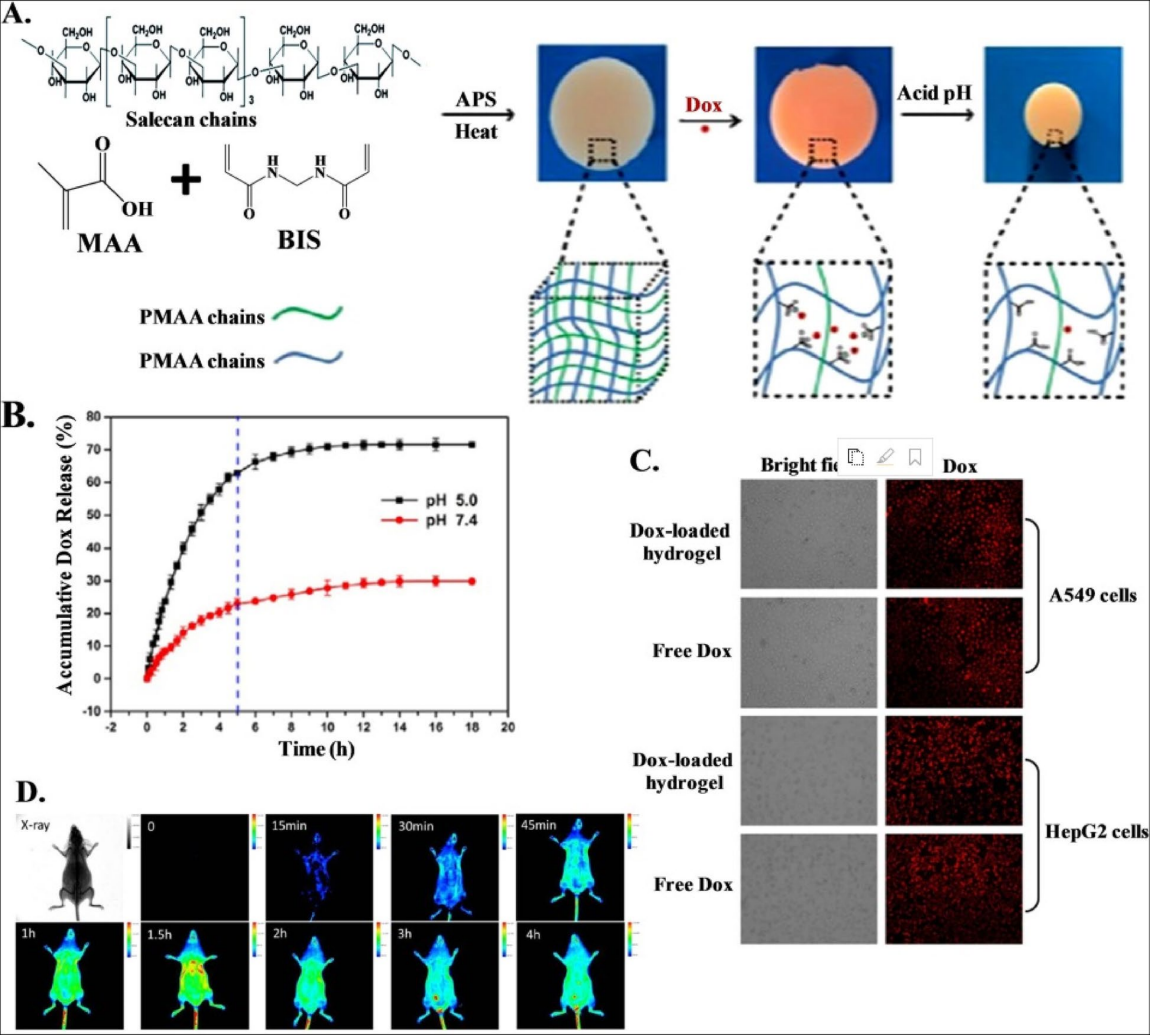
The use of hydrogel as a vehicle for controlling drug delivery. **A** the process of preparing and releasing drugs from Salecan/PMAA semi-IPN hydrogels; **B** the in vitro behavior of Dox release from the semi-IPN sample under two different pH values; **C** images obtained using fluorescent microscopy of A549 and HepG2 cells after 4 h of incubation with 6 μg/mL free Dox solutions and the extract liquid of Dox-loaded hydrogel; and (**D**) real-time fluorescence images of FITC-labeled PMAA nanohydrogels in ICR mice. Reprint from [180] with a permission from Springer Nature

**Fig. 11:**
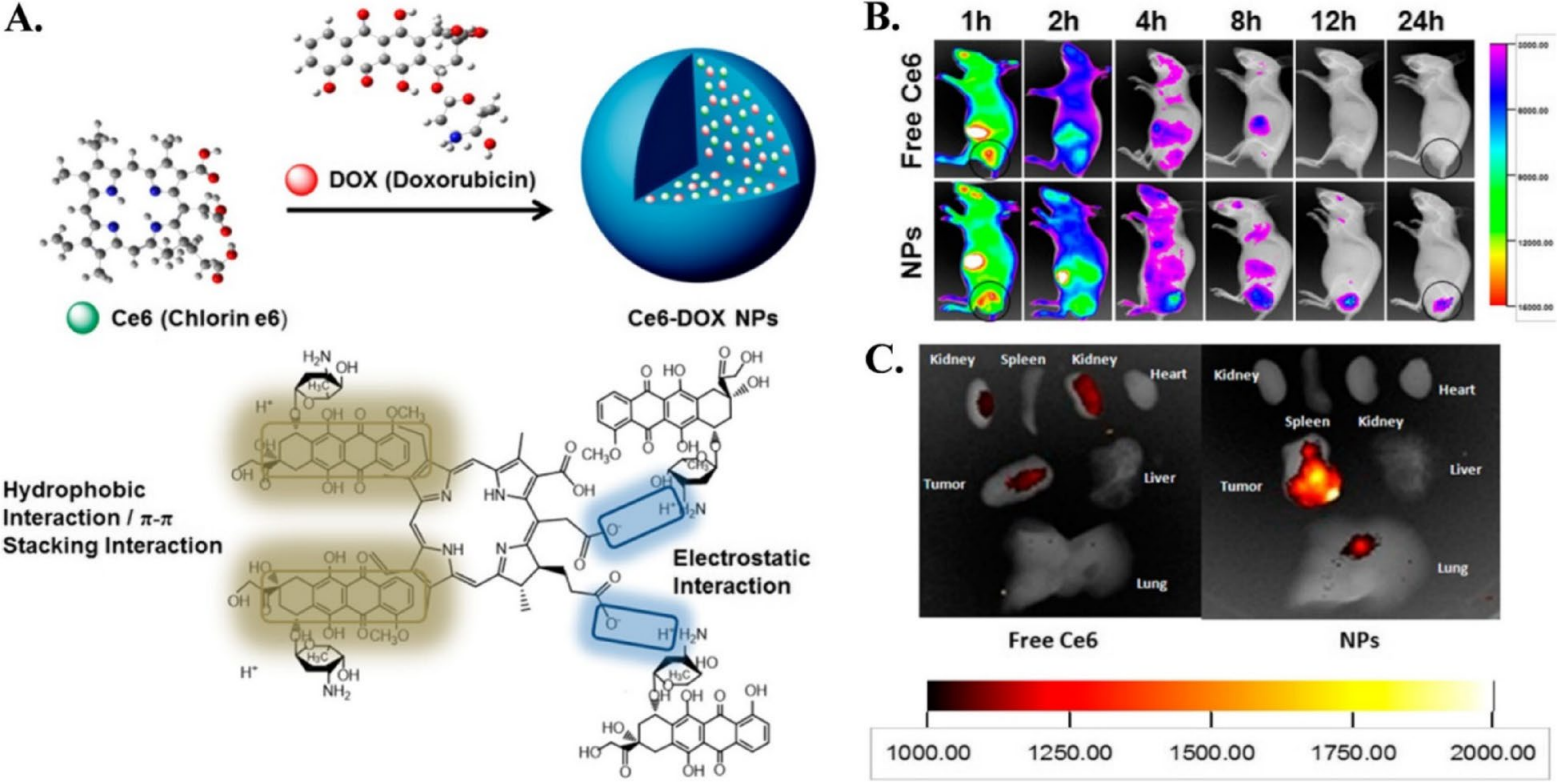
The co-assembly of a drug and a photosensitizer to improve tumor size imaging during treatment. **A** A diagram illustrating the creation of carrier-free nanoparticles (NPs) through the co-assembly of DOX and Ce6. **B** In vivo fluorescence images of free Ce6 solution and Dox/Ce6 nanoparticles (NPs) are presented. The black circles indicate the tumor tissue. **C** Representative ex vivo fluorescence images of the tumor and organs from Balb/c nude mice xenografted with MCF-7 tumor, 24 h after injection, are displayed. Reprint from [180] with a permission from Springer Nature

The high surface-to-volume ratio of PNPs is similar to that of nanoscale particles, making it easy to attach targeting polymers to the particle's surface [13]. Bioavailability may be improved by coating polymers with polysorbates since this makes use of the surfactant activity of polysorbates by solubilizing and fluidizing endothelial cell membranes [10, 12]. By having a coating on their surface, PNPs are better able to interact with the endothelial cells that make up the blood–brain barrier (BBB), facilitating their endocytosis. Because new nanocarriers work differently than traditional chemical treatments, polymeric nanoparticles may carry a wide variety of chemicals to specific areas [88]. Anticancer medicines, small interfering RNAs (siRNA), radionuclides, and ultrasonic wave-reactive polymeric nanoparticles are all examples of such compounds. Fluorescent polymeric nanoparticles have been shown to be valuable tools in the area of theragnostics. The phrase "theragnostic" refers to a procedure that combines "diagnosis" and "treatment" in the same sentence. Fluorescent polymeric nanoparticles (FNPs) have recently come to the forefront as a promising new therapeutic material. Complex nanomaterial structures might be designed to serve dual diagnostic and therapeutic purposes [142]. Fluorescent protein networks (FNPs) are typically constructed from biocompatible biopolymers, inorganic quantum dots, organic dyes, and fluorescent proteins [49]. To improve nanomedicine's efficacy against cancer, drugs might be loaded through bonds or hydrophobic contacts in fluorescence tests. Not only is this in addition to imaging for tumors, but it is an integral part of it. Delivering siRNA more efficiently in vivo has been shown using cyclodextrin polymer (CDP)-based nanoparticles. Research has revealed that adamantane-polyethylene glycol (AD-PEG) modified with transferrin and adamantane-PEG-transferrin (AD-PEG-Tf) are both effective in vivo nucleic acid delivery vehicles [49]. Nanoparticles might be used to encapsulate radionuclides like I125 by a technique called electrophilic aromatic substitution, which leads to high radiochemical yields. This easy procedure might be used to keep the radioactive substance in the core where it is most stable [178, 179]. Dey created an 11 nm-diameter, self-assembling peptide/protein nanoparticle. This nanoparticle performed well in terms of biocompatibility and in vivo stability, suggesting it might be useful for drug delivery in cancer therapy [57]. Figure 10 illustrates the use of hydrogel as a means to control drug delivery. The process of preparing and releasing drugs from Salecan/PMAA semi-IPN hydrogels is shown in (Fig. 10-A). In (Fig. 10-B), the in vitro behavior of Dox release from the semi-IPN sample under two different pH values is depicted. The images obtained using fluorescent microscopy of A549 and HepG2 cells after 4 h of incubation with 6 μg/mL free Dox solutions and the extract liquid of Dox-loaded hydrogel are shown in (Fig. 10-C). Lastly, real-time fluorescence images of FITC-labeled PMAA nanohydrogels in ICR mice are presented in (Fig. 10-D). The use of hydrogels as a vehicle for controlling drug delivery is a promising method that can improve the efficacy and safety of drug therapies by providing controlled and sustained release of drugs at specific sites. Recently, ultrasound-sensitive polymeric nanoparticles have emerged as a useful tool for cancer diagnosis and treatment. Applications for ultrasound-interactive nanoparticles have multiplied [89]. Ultrasound is employed in the synthesis of NPs to improve their distribution efficiency; this, in turn, reduces the likelihood of adverse effects from the increased ability to overcome barriers to cancer therapy. These include the nuclear membrane, interstitium, interstitial fluid, and the endothelium that lines blood vessels and tissue [15]. Figure 11 shows a new method for improving tumor size imaging during treatment. The approach involves the co-assembly of a drug called DOX and a photosensitizer named Ce6 to form carrier-free nanoparticles. The nanoparticles were tested in vivo and ex vivo on Balb/c nude mice with MCF-7 tumor xenografts. The in vivo fluorescence images show that the Dox/Ce6 nanoparticles produced a much stronger fluorescence signal in the tumor tissue compared to the free Ce6 solution. The ex vivo fluorescence images also reveal that the nanoparticles have accumulated significantly in the tumor tissue and not in other organs. This co-assembly of the drug and the photosensitizer could potentially improve the monitoring of tumor response to treatment and thus improve cancer management. To further facilitate the controlled release of chemical treatments, ultrasound may be used as a planned trigger. This is possible because ultrasonic can generate a heat effect, which may finally cause the nanoparticles to fracture [15]. Because ultrasound has a heating impact, it allows for this to happen. There is evidence that certain polymeric nanoparticles undergo hazardous breakdown and toxic monomer aggregation, so additional study is needed to improve the manufacturing of these nanoparticles and their chemical characteristics [110].

Lymphatic drainage refers to the natural clearance mechanism of the lymphatic system, responsible for draining interstitial fluid and foreign particles from tissues [181]. In the context of nanomedicine delivery, lymphatic drainage plays a critical role. It leads to the rapid clearance of nanoparticles from the injection site, reducing their retention and bioavailability at the target site. This can be particularly problematic for nanomedicines designed for targeted drug delivery or immunotherapy in lymph nodes [182]. Additionally, if nanomedicines enter the systemic circulation due to lymphatic drainage, there's an increased risk of systemic toxicity and side effects associated with these therapeutic agents [183]. Vessel wall leakage, or the permeability of blood vessel walls, has a significant impact on the distribution of nanomedicines in the body [184]. The enhanced permeability and retention (EPR) effect, a result of vessel wall leakage in tumor vasculature, can be harnessed for the targeted delivery of nanomedicines to cancerous tissues. This effect allows nanomedicines to accumulate in tumor tissues, improving treatment efficacy for cancer patients [185]. However, vessel wall leakage in normal vasculature can also lead to non-specific drug delivery to healthy tissues, increasing the risk of off-target effects [184]. The extent of vessel wall leakage can vary based on factors like inflammation, disease state, and the formulation of the nanomedicine. Researchers and scientists have developed various strategies to mitigate the impact of lymphatic drainage and vessel wall leakage on nanomedicine delivery. These strategies include engineering nanoparticles with surface modifications that help evade lymphatic drainage, thus prolonging their circulation time and improving targeting. Utilizing targeting ligands for active uptake by specific cells or tissues is another approach, enhancing precision and reducing off-target effects [183]. Nanocarrier design plays a role in exploiting the EPR effect while minimizing non-specific leakage. Additionally, developing drug formulations with controlled release profiles can help sustain therapeutic effects and reduce systemic toxicity. Several case studies exemplify the impact of lymphatic drainage and vessel wall leakage on nanomedicine delivery. Liposomal doxorubicin (Doxil), for instance, effectively utilizes the EPR effect to target tumor tissues in cancer therapy [185]. PEGylated nanoparticles are designed to prolong circulation by reducing lymphatic drainage, thus enhancing retention at the target site. Antibody–drug conjugates (ADCs) in targeted cancer therapy demonstrate specific cell targeting, minimizing off-target effects [184]. Liposomal amphotericin B (AmBisome) for fungal infections showcases controlled release, which not only ensures sustained therapeutic effects but also minimizes systemic toxicity. These case studies illustrate how different nanomedicine formulations and strategies can be tailored to optimize drug delivery based on the interplay between lymphatic drainage and vessel wall leakage [183].

The advancement of nanotechnology in cancer therapy represents a remarkable leap forward in the quest to combat this devastating disease [64]. The use of nanoscale materials and engineered carriers has introduced a level of precision and specificity that was previously unimaginable in cancer treatment [126]. One of the key qualities of nanotechnology in this context is its ability to target cancer cells with unprecedented accuracy [186]. By designing nanoparticles or nanocarriers that can selectively seek out and bind to cancerous cells while sparing healthy tissue, nanotechnology offers a highly targeted approach to therapy. This selectivity minimizes the collateral damage associated with conventional treatments like chemotherapy, reducing side effects and improving the overall quality of life for cancer patients [63]. Furthermore, nanotechnology has the potential to enhance the delivery of therapeutic agents to tumor sites. These nanocarriers can carry a variety of payloads, including chemotherapy drugs, antibodies, or nucleic acids, and release them specifically within the tumor microenvironment. This not only increases the effectiveness of the treatment but also reduces the systemic exposure to toxic agents, mitigating adverse effects [187]. The ability to encapsulate and deliver drugs precisely where they are needed within the body has the potential to significantly improve the efficacy of cancer therapies while minimizing the harm to healthy tissues [188]. The impact of nanotechnology in cancer therapy is already being felt in the realm of clinical translation [186]. While there have been numerous exciting developments in the laboratory, translating these findings to the clinic remains a complex challenge [63]. Regulatory approvals, safety assessments, and scalability are among the hurdles that researchers and pharmaceutical companies must overcome [186]. Nevertheless, several nanocarriers and formulations have successfully made their way into clinical trials and, in some cases, received authorization for clinical use [187]. These early successes demonstrate the tangible impact of nanotechnology in cancer therapy, offering patients new hope and treatment options. As researchers continue to refine and expand upon these technologies, the future holds even greater promise for harnessing nanotechnology's full potential in the fight against cancer. In addition to improving the precision and effectiveness of cancer treatment, nanotechnology is also contributing to advancements in cancer diagnosis and monitoring [64]. Nanoscale materials can be engineered to detect specific biomarkers or tumor-associated molecules at incredibly low concentrations. This capability has paved the way for highly sensitive diagnostic tests and imaging techniques that can detect cancer at its earliest stages when treatment is often most successful [187]. These diagnostic tools not only aid in early detection but also allow for real-time monitoring of a patient's response to therapy, enabling healthcare providers to make timely adjustments to treatment plans [186]. Furthermore, the interdisciplinary nature of nanotechnology has fostered collaborations between experts in various fields, such as chemistry, biology, physics, and engineering [187]. This interdisciplinary approach has accelerated progress in cancer research and led to innovative solutions that would not have been possible without nanotechnology. It has also spurred the development of novel theranostic approaches, where diagnostics and therapy are combined into a single nanoscale system, offering a holistic approach to cancer care [63]. Despite these promising developments, challenges in clinical translation persist [186]. Issues related to the long-term safety and biocompatibility of nanomaterials, as well as concerns about potential unforeseen side effects, must be thoroughly addressed [64]. Additionally, the cost of manufacturing and scaling up nanocarrier production can be prohibitive. Regulatory agencies around the world are working to establish clear guidelines for the approval of nanotechnology-based cancer therapies, but the process remains complex [186].

### Monoclonal nanoparticle antibodies

**Fig. 12:**
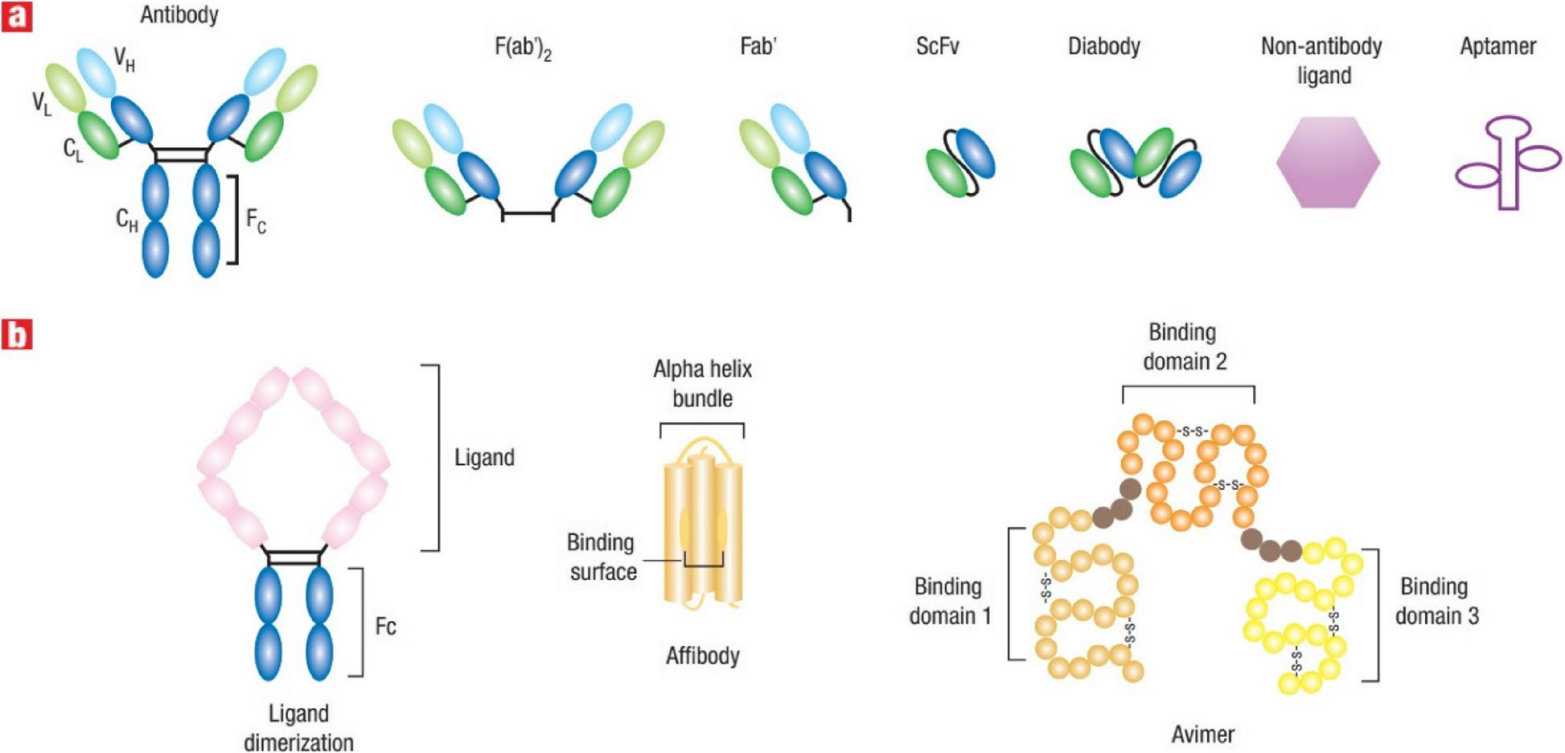
Different types of targeting agents and strategies to enhance their affinity and selectivity in two parts. Part a shows various targeting molecules, such as monoclonal antibodies or fragments, non-antibody ligands, and aptamers. Antibody fragments, such as F(ab')2 and Fab', are generated by enzymatic cleavage, while molecular biology techniques produce Fab', scFv, and bivalent scFv (diabody) fragments. The antibody structure comprises the variable heavy chain (vH), variable light chain (vL), constant heavy chain (CH), and constant light chain (CL). Non-antibody ligands consist of vitamins, carbohydrates, peptides, and other proteins. Aptamers can be made up of DNA or RNA. Part b outlines methods to enhance affinity and selectivity, such as ligand dimerization or screening for conformation-sensitive targeting agents like affibodies, avimers, and nanobodies. Ligand dimerization involves linking two ligands together, which increases binding affinity. Conformation-sensitive targeting agents are proteins that recognize specific three-dimensional structures and differentiate between closely related molecules. Intact antibodies and their fragments are also useful for enhancing affinity and selectivity. Reprint from [132] with a permission from Springer Nature

**Fig. 13:**
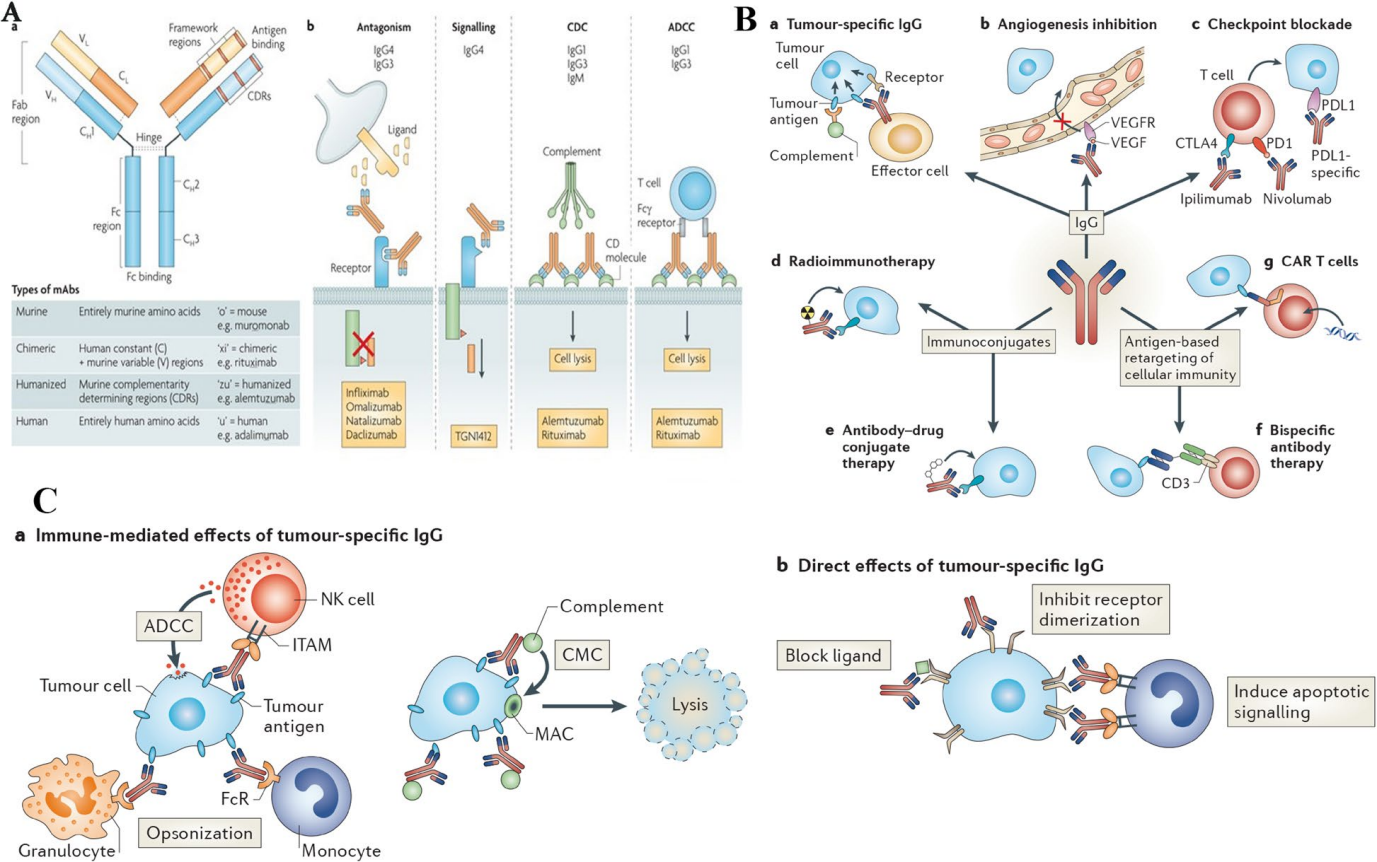
**A** The evolution and characteristics of monoclonal antibodies (mAbs) in terms of their structure and function. The different types of mAbs that have been developed over time, starting from murine mAbs and progressing to chimeric mAbs, humanized mAbs, and fully human mAbs. Reprint from [191] with a permission from Lancet Publishing Group. **B** Various strategies employed in monoclonal antibody (mAb) cancer therapeutics. Various strategies employed in monoclonal antibody (mAb) cancer therapeutics include targeting specific cancer cell surface antigens, blocking signaling pathways crucial for tumor growth, enhancing the immune system's ability to recognize and destroy cancer cells, and conjugating mAbs with toxins to deliver targeted cytotoxic effects. These diverse approaches have contributed to the success of mAb therapies in treating cancer. Reprint from [192] with a permission from Springer Nature. **C** The mechanisms of action of monoclonal antibodies (mAbs) that specifically target cancer cells. These mAbs exert their antitumor effects through various means, which are commonly studied in laboratory settings. However, determining the individual contributions of these mechanisms to the clinical responses observed during mAb therapy is challenging. Reprint from [192] with a permission from Springer Nature

Figure 12 illustrates the various types of targeting molecules that can be used in medicine, including monoclonal antibodies, non-antibody ligands, and aptamers. Enzymatic cleavage or molecular biology techniques can be used to create antibody fragments such as F(ab')2, Fab', scFv, and bivalent scFv (Diabody). Non-antibody ligands can include vitamins, carbohydrates, peptides, and other proteins, while aptamers can be composed of either DNA or RNA. The panel also shows how affinity and selectivity can be improved through ligand dimerization or by screening for conformational-sensitive targeting agents such as affibodies, avimers, nanobodies, as well as intact antibodies and their fragments. These techniques can help to create more effective and precise targeting agents for use in medical treatments. There have been some promising recent advancements in the realm of mAb nanoparticles. Due to their specific targeting ability and anti-tumor efficacy, monoclonal antibodies (mAbs) are widely utilized in the area of targeted treatment [189]. The use of mAbs in the creation of novel anti-tumor nanoplatforms has also been a driving force in the field in recent years. Improved specificity and reduced toxicity may be obtained by directing the drug combination toward antigens that are differentially expressed between malignant and healthy cells [189]. An antibody–drug conjugate (ADC) is a method of boosting the effectiveness of anticancer medications in treatment. Cytotoxic medicines are attached to mAbs [49, 50]. Patients with breast cancer and an overexpression of human epidermal growth factor receptor 2 are often administered the monoclonal antibody Herceptin (or trastuzumab) (HER2) [190]. The use of trastuzumab (Tmab) in the ADC system has been studied, and the findings imply increased therapy efficacy compared to utilizing Tmab alone [49, 50]. Using paclitaxel (PTX) as the core medication and trastuzumab as the surface modification, Abedin et al. developed an antibody–drug nanoparticle [189]. This kind of nanoparticle proved effective in its targeting of breast cancer cells. Better anti-tumor activity was shown with the NP complex compared to either PTX or trastuzumab alone, and less cytotoxicity was seen in the control of human breast epithelial cells when using the NP complex [35]. Numerous studies are currently being conducted on trastuzumab nanoparticles (NPs) based on the ADC mechanism as potential nanoplatforms in the treatment of cancer [189]. Two HER2-positive cell lines and one HER2-negative cell line were given the novel NP, PTX, and trastuzumab, respectively. The results were promising: the NP complex showed better anti-tumor efficacy than PTX or trastuzumab [49, 50]. Figure 13-A provides an overview of the structural development of mAbs and highlights their various functions, which can range from antagonism to signaling, mediated by specific regions within the mAb structure. The structure of an immunoglobulin G (IgG) mAb is schematically represented in Fig. 13-A-a. It consists of a Fab region and an Fc region. The Fab region contains variable (V) regions that bind to specific targets, and it has undergone modifications in the development of mAbs. Murine mAbs initially had fully murine V regions, while chimeric mAbs had murine V regions grafted onto human constant (C) regions. Humanized mAbs retained a human Ig scaffold, with only the complementarity-determining regions (CDRs) derived from murine origin. Finally, fully human mAbs were generated, indicating that their entire structure is derived from human components. The Fc region of a mAb includes the hinge and constant heavy-chain domains (CH2 and CH3) and serves various functions such as complement fixation or binding to Fc receptors. The nomenclature of mAbs reflects their type, with indicators like 'xi' for chimeric mAbs (e.g., rituximab). Figure 13-A-b of the figure highlights the functions of mAbs, which are influenced by specific CDRs within the Fab region. Some mAbs can bind to ligands or receptors, preventing their stimulation and exhibiting antagonism. Examples of ligand-binding mAbs are infliximab and omalizumab, while receptor-binding mAbs include natalizumab and daclizumab. On the other hand, certain mAbs can induce signal transduction by binding to receptors. TGN1412, a CD28 superagonist, is an example of a mAb that activates T-cells without the need for T-cell receptor ligation. The Fc region of mAbs controls additional functions, such as complement-dependent cytotoxicity (CDC), antibody-dependent cell-mediated cytotoxicity (ADCC), and antibody-dependent cellular phagocytosis. CDC involves cell lysis through complement activation, while ADCC involves the binding of mAbs to Fc receptors, leading to cell lysis. Furthermore, the binding of mAbs to the neonatal Fc receptor influences their transport across cell barriers and affects their half-life [191]. Figure 13-B illustrates various monoclonal antibody-based therapeutic strategies for cancer treatment. The immunoglobulin G (IgG) molecules can bind to cancer cells (Fig. 13-B-a) and trigger immune effector cells to carry out antibody-dependent cellular cytotoxicity (ADCC). They can also induce complement-mediated cytotoxicity (CMC) or directly induce the death of cancer cells through signaling pathways (e.g., herceptin and rituximab). In addition, IgG mAbs can hinder angiogenesis (Fig. 13-B-b) (e.g., bevacizumab) or block inhibitory signals (part c), resulting in a stronger T cell response against tumors (e.g., ipilimumab and nivolumab). Radioimmunoconjugates (part d) (e.g., 131I tositumomab and ibritumomab tiuxetan) deliver radioisotopes to cancer cells, while antibody–drug conjugates (Fig. 13-B-e) (e.g., brentuximab vedotin and trastuzumab emtansine) deliver potent toxic drugs to cancer cells. The variable regions of mAbs are also utilized to redirect immune effector cells towards cancer cells using bispecific mAbs that recognize cancer cells with one arm and activating antigens on immune effector cells with the other arm (Fig. 13-B-f) (e.g., linatumomab). Another approach involves a gene therapy technique where DNA for a mAb variable region fused to signaling peptides is transferred to T cells, thereby creating chimeric antigen receptor (CAR) T cells (Fig. 13-B-g) that specifically target tumors. In the figure, several key molecules are labeled, including CD3, CTLA4, PD1, PDL1, VEGF, and VEGFR, which play important roles in these therapeutic strategies [192]. Figure 13-C illustrates the mechanisms by which monoclonal antibodies (mAbs) that target cancer cells exert their anti-tumor effects. One mechanism involves the ability of mAbs to facilitate antibody-dependent cellular cytotoxicity (ADCC) by engaging immune effector cells expressing immunoreceptor tyrosine-based activation motifs (ITAMs). Examples of such cells include natural killer (NK) cells, monocytes, macrophages, and granulocytes. Upon binding to cancer cells, the mAbs can trigger ADCC, leading to the destruction of the target cells. Additionally, the fixation of complement, a component of the immune system, can enhance the process by promoting opsonization (coating of the target cell) and facilitating phagocytosis and lysis by monocytes and granulocytes. Complement-mediated cytotoxicity (CMC) can directly induce target cell death through the formation of a membrane attack complex (MAC) (Fig. 13-C-a). Another mechanism employed by mAbs involves their direct effects on target cells. They can block the binding of activating ligands responsible for the survival of cancer cells. By doing so, mAbs prevent the activation signal from reaching the cancer cells, inhibiting their growth and survival. Additionally, mAbs can inhibit receptor dimerization, which is necessary for activation, thereby blocking the activation signal. Furthermore, mAbs can induce an apoptotic signal in cancer cells by crosslinking specific receptors. This receptor crosslinking can be enhanced when mAbs are bound to Fc receptor-expressing cells. The Fc receptor-binding promotes the clustering of mAbs and enhances the apoptotic signal. Immunoglobulin G (IgG) is the subclass of antibodies commonly used in this context (Fig. 13-C-B) [192].

### Membrane-bound packets are found outside of cells

Bilayer phospholipids make up EVs,. The vast majority of extracellular vacuoles can be classified into three broad groups: exosomes, microvesicles, and apoptotic bodies (EVs). Exosomes are 40–200 nm nano-scale particles. EVs are involved in long-distance communication and have the capacity to transport protein, RNA, and DNA in their bodies [96]. Exosome NPs are natural carriers that may be used with known anti-tumor compositions and procedures. This is owing to the fact that the membrane of exosomes includes lipids and chemicals that are comparable to those present in the cells from whence they originated [15]. This enables exosome NPs to avoid immune monitoring and integrate seamlessly with target cells. In order to be successful in treating cancer, gene therapy requires the use of DNA and RNA. In gene therapy, various alternative ways are being researched [30]. These include reactivating mutated proto-oncogenes like p53, inhibitor of growth 4 (ING4), and phosphatase and tensin homolog (PTEN), as well as gene editing with the clustered regularly interspaced short palindromic repeats (CRISPR)-associated proteins (Cas) system, which inhibits the activity of key oncogenes. Some of these small RNAs, such as siRNAs and microRNAs, may trigger RNAi (RNA interference) (miRNAs) [193]. Multiple physiological and pathological processes include RNA interference (RNAi). Research using siRNA to target oncogenic mRNAs is currently being assessed. Gene therapy is another way that may be used to deliver a transgene or a cell death-inducing gene to cancer cells [194, 195]. Exosomes have been successfully used as nanoparticle platforms for the delivery of nucleic acids, tiny chemicals, and proteins [30]. Human breast cancer cells were treated with doxorubicin-loaded exosomes by the group of Hadla et al. (exoDOX). The findings demonstrated that exoDOX enhances doxorubicin's cytotoxicity and prevents drug accumulation in the heart compared to free doxorubicin [174]. Targeted delivery in the treatment of cancer may be possible via the engineering of exosomes. Macrophage-derived exosomes were modified using an aminoethylanisamide-polyethylene glycol (AA-PEG) moiety, and subsequently PTX was transferred to the modified exosomes [30]. The modified exosome greatly improved therapy effectiveness in a mouse model of lung metastases. Jeong et al. used exosomes to deliver miR-497 (microRNA-497) to A549 cells. These data suggest that an exosome-mediated miRNA therapy might be employed for the targeted treatment of cancer, since both tumor growth and the expression of associated genes were suppressed [169]. In contrast to synthetic nanoparticles, exosome nanoparticles benefit from inherent biocompatibility, higher chemical stability, and the ability to regulate intercellular connections (NPs). However, there are obstacles to the widespread use of exosome NP, including the lack of standardized criteria for isolating and purifying exosomal components; the lack of a well-defined mechanism for exosomes' role in cancer therapy; the phenomenon of heterogeneity; and the difficulty of preserving exosomes [30]. Tumor cell exosomes are minute vesicles secreted by cancer cells that play a pivotal role in intercellular communication and the progression of cancer [196]. These exosomes are found outside of cells, typically circulating in bodily fluids such as blood and urine [197]. Nanocarriers, a cutting-edge technology in the field of nanomedicine, have been harnessed to target and deliver therapeutic payloads to these tumor cell exosomes. By encapsulating drugs or genetic material within nanocarriers, researchers can achieve precise and efficient drug delivery to cancer cells [198]. Additionally, membrane-bound packets, akin to exosomes but originating from different cellular sources, can also be found outside of cells and are under investigation for their potential in therapeutic applications. Understanding the intricate interactions between tumor cell exosomes, nanocarriers, and membrane-bound packets offers promising avenues for developing innovative cancer treatments [199].

### Lipid-based nanomaterials

**Fig. 14:**
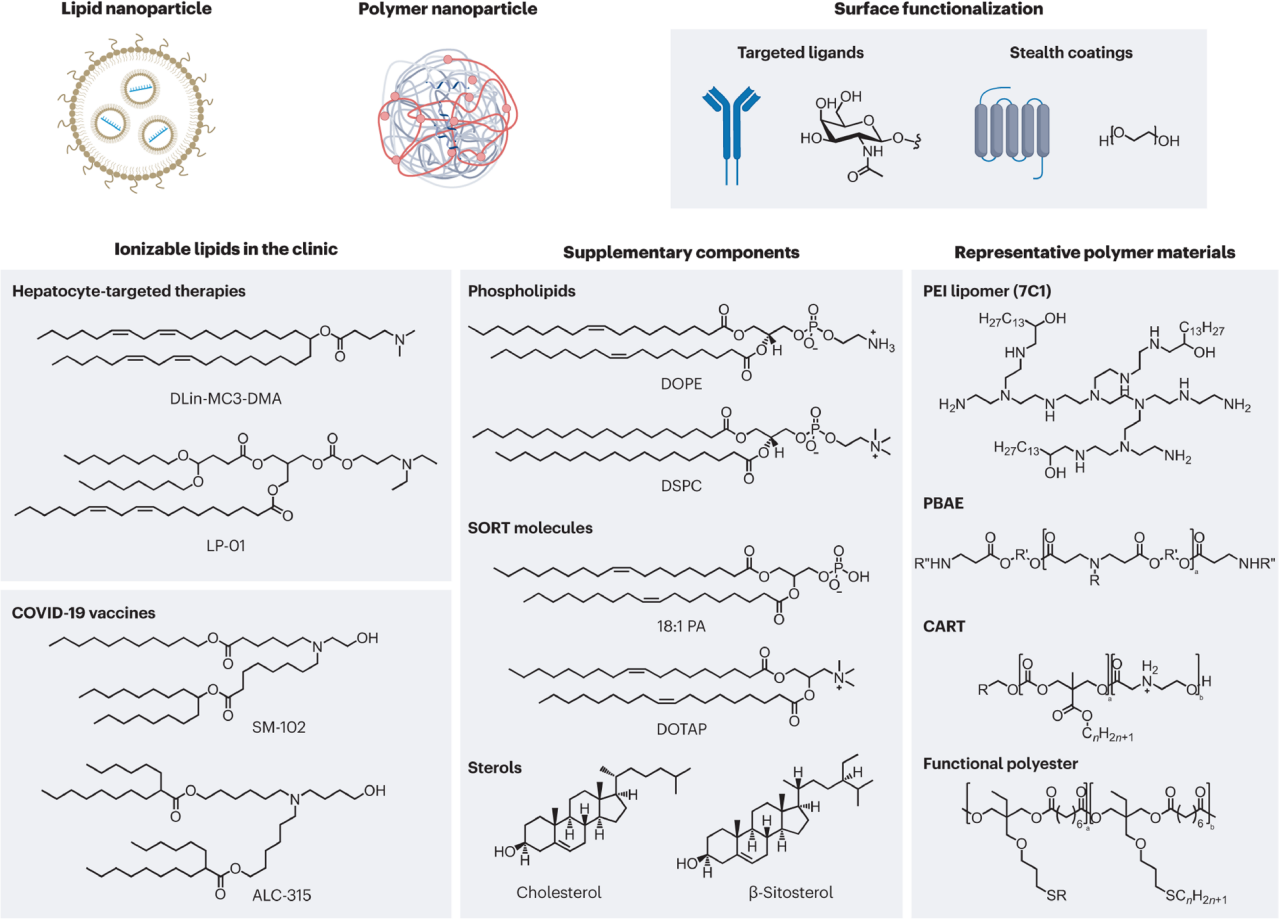
The cutting-edge development in the field of genetic drug delivery using self-assembled nanoparticles made from lipid and polymer materials. Currently, the most advanced system for delivering genetic drugs in clinical settings is lipid nanoparticles incorporating an ionizable lipid. These materials contain a tertiary amine that can acquire a charge at acidic pH, enabling the loading of nucleic acids during formulation and facilitating their release from endosomes after cellular uptake. Examples of ionizable lipids include Dilinoleylmethyl-4-dimethylaminobutyrate (DLin-MC3-DMA) found in the FDA-approved drug Onpattro, LP-01 in Intellia Therapeutics' clinical candidates NTLA-2001 and NTLA-2002 for liver gene editing, and SM-102 and ALC-315, which are ionizable lipid components of the Moderna and Pfizer-BioNTech vaccines, respectively. Alternatively, certain polymers containing ionizable amine groups can also be utilized for nanoparticle formulation, with the choice of monomers affecting delivery efficiency and tissue selectivity. In both ionizable lipids and polymers, additional components can be added to enhance nanoparticle stability, fusogenicity (ability to merge with cellular membranes), and selectivity. Furthermore, the surfaces of these nanoparticles can be modified using synthetic or biological targeting ligands and stealth coatings to alter their circulation time, biodistribution, and cellular uptake. By loading nucleic acid biomolecules into nanoparticles, it becomes possible to reprogram the fundamental principles of biology through gene silencing, expression, and editing to correct disease processes. 18:1 PA (1,2-dioleoyl-sn-glycero-3-phosphatidic acid), CART (charge-altering releasable transporter), DOPE (1,2-dioleoyl-sn-glycero-3-phosphoethanolamine), DOTAP (1,2-dioleoyl-3-trimethylammonium-propane), DSPC (1,2-distearoyl-sn-glycero-3-phosphocholine), PBAE (poly(beta-amino ester)), PEI (polyethyleneimine), SORT (selective organ targeting). Reprint from [200] with a permission from Springer Nature

The three primary types of lipid carriers that have been the focus of recent studies and clinical trials are liposomes, solid lipid nanoparticles (SLNs), and nanostructured lipid carriers. Figure 14 illustrates the current advancements in the field of delivering genetic drugs using self-assembled nanoparticles made from lipid and polymer materials. The study of lipid-based nanomaterials is growing, with a particular focus on these three areas (NLCs) [57]. It wasn't until 1965 that liposomes received formal recognition as the first encapsulated tiny phospholipid bilayer nanosystem. Liposomes are vesicles that may be either spherical or ovoid and are composed mostly of phospholipids. On average, a liposome may range in size from 20 nm to over 1 µm [13]. The hydrophobic phospholipid bilayer surrounding the hydrophilic center is what makes up a liposome. This kind of structure may entrap both hydrophilic and hydrophobic medications, depending on the pharmacokinetic properties of the treatment [13]. In a recent groundbreaking study conducted by Rosenblum et al., the limitations of CRISPR-Cas9 technology in cancer therapeutics have been addressed through the development of a novel delivery system. The study highlights the challenges of low editing efficiency in tumors and potential toxicity associated with existing delivery methods. The research introduces a promising solution in the form of LNPs specifically engineered for targeted delivery of Cas9 mRNA and sgRNAs. These LNPs utilize an innovative amino-ionizable lipid, which significantly enhances their safety and efficiency. In the context of cancer treatment, the researchers demonstrated the remarkable potential of these LNPs. Intracerebral injection of CRISPR-LNPs against PLK1 into glioblastoma resulted in up to ~ 70% gene editing in vivo, leading to tumor cell apoptosis, a 50% reduction in tumor growth, and a 30% improvement in survival. Furthermore, LNPs engineered for antibody-targeted delivery exhibited exceptional efficacy against disseminated ovarian tumors, achieving up to ~ 80% gene editing in vivo, suppressing tumor growth, and increasing survival by 80%. This innovative approach to CRISPR-Cas9 genome editing, utilizing nanocarriers, opens new avenues for cancer treatment and research, showcasing its potential for precise gene editing not only in cancerous tissues but also in noncancerous ones [98]. Table 8 presents the pharmacokinetic profiles of various nanocarrier-loaded drugs.

**Table 8:** Pharmacokinetic profiles of nanocarrier-loaded drugs

**Drug Type**	**Administration Route**	**Pharmacokinetic Parameter**	**Clearance Pathway**	**Drug Interaction**	**Therapeutic Window**	**References**
**Doxorubicin**	Intravenous	Area Under Curve (AUC)	Hepatic Metabolism	P-glycoprotein	Narrow	[201]
**Paclitaxel**	Intravenous	Half-life	Renal Excretion	Cytochrome P450	Wide	[57]
**Cisplatin**	Intraperitoneal	Volume of Distribution	Renal Excretion	None	Narrow	[90, 102]
**Irinotecan**	Oral	Bioavailability	Hepatic Metabolism	UDP-glucuronosyltransferase	Wide	[202]
**Methotrexate**	Intrathecal	Cerebrospinal Fluid Concentration	Renal Excretion	None	Narrow	[99]
**Gemcitabine**	Intravenous	Clearance Rate	Renal Excretion	Deoxycytidine Kinase	Wide	[203]
**Etoposide**	Intravenous	Distribution Half-life	Hepatic Metabolism	Cytochrome P450	Wide	[204]
**Oxaliplatin**	Intravenous	Total Clearance	Renal Excretion	None	Narrow	[205]
**Topotecan**	Oral	Bioavailability	Hepatic Metabolism	Cytochrome P450	Narrow	[206]
**Docetaxel**	Intravenous	Protein Binding	Hepatic Metabolism	P-glycoprotein	Wide	[207]
**Methotrexate**	Intravenous	Clearance	Renal Excretion	None	Narrow	[208]
**Trastuzumab**	Intravenous	Volume of Distribution	Proteolysis	None	Wide	[189]
**Docetaxel**	Intravenous	Protein Binding	Hepatic Metabolism	P-glycoprotein	Wide	[117]
**Bleomycin**	Intravenous	Half-life	Renal Excretion	None	Narrow	[209]
**Vinorelbine**	Oral	Bioavailability	Hepatic Metabolism	P-glycoprotein	Wide	[210]
**Daunorubicin**	Intravenous	AUC	Hepatic Metabolism	P-glycoprotein	Narrow	[211]
**Cisplatin**	Intravenous	Half-life	Renal Excretion	None	Narrow	[212]
**Pemetrexed**	Intravenous	Protein Binding	Renal Excretion	None	Narrow	[213]
**Everolimus**	Oral	Bioavailability	Hepatic Metabolism	Cytochrome P450	Wide	[214]
**Tamoxifen**	Oral	Clearance	Hepatic Metabolism	None	Wide	[215]

**Fig. 15:**
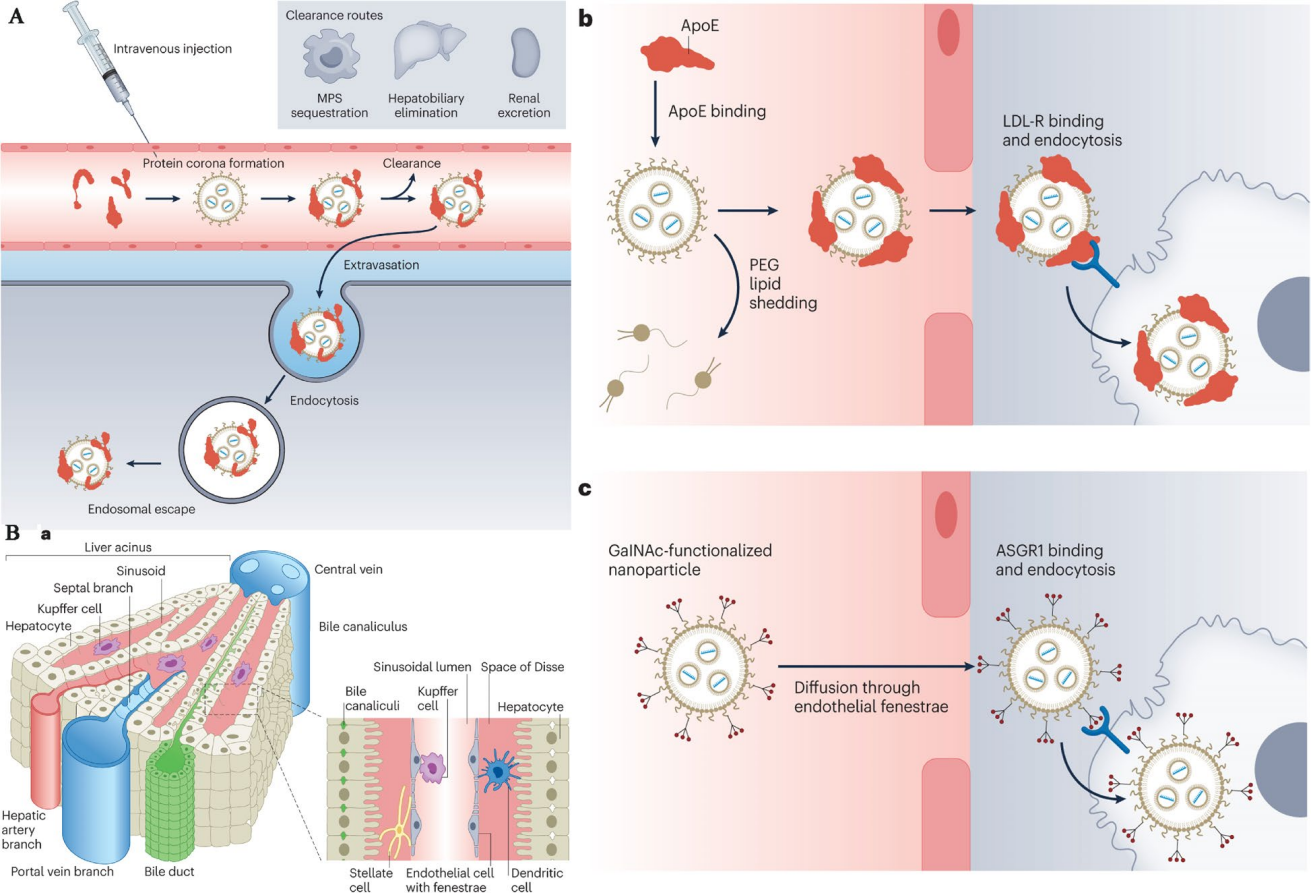
**A** The path of a tiny particle within the human body after it is injected intravenously. When the particle enters the bloodstream, it often attracts plasma proteins, forming a layer called the protein corona on its surface. The composition of this corona is affected by the properties and makeup of the particle's surface. In order to reach the intended organ, the particle needs to leave the blood vessels (a process known as extravasation) by either passing through gaps in the endothelium (a size-dependent mechanism) or actively interacting with specific receptors on the endothelium through transcytosis. After extravasation, the particle must interact with target cells and be internalized by them. It must then escape from the endosome into the cytosol and release its genetic payload. Throughout this journey, the particle can be eliminated from the bloodstream through various mechanisms such as the mononuclear phagocytic system (MPS), hepatobiliary elimination via feces, or renal excretion through urine. These processes restrict the amount of the injected particle dose that actually reaches the intended target site. Therefore, measures must be taken to minimize their impact. Reprint from [200] with a permission from Springer Nature. **B** Lipid nanoparticles have reached an advanced stage of development for delivering genetic drugs to the liver. a) The liver consists of four distinct types of cells. When nanoparticles are present in the bloodstream, they can be captured by Kupffer cells, absorbed by liver sinusoidal endothelial cells, or pass through the wide openings in the liver endothelium into the Space of Disse. In the Space of Disse, the nanoparticles can target hepatic stellate cells or hepatocytes. The hepatobiliary system can eliminate nanoparticles from the body through the bile duct. b) A clinically validated approach for delivering small interfering RNA to hepatocytes involves the natural targeting of liver cells. For instance, in the case of Onpattro lipid nanoparticles, the polyethylene glycol (PEG) lipid on the surface of the nanoparticles is exchanged with apolipoprotein E (ApoE) in the blood. The binding of ApoE to the nanoparticle surface enables its interaction with the low-density lipoprotein receptor (LDL-R), which is highly expressed by hepatocytes, leading to endocytosis. c) Another way to actively target hepatocytes is by modifying the nanoparticle surface with a ligand called N-acetylgalactosamine (GalNAc) and reducing non-specific protein binding through extensive PEGylation. GalNAc binds to the asialoglycoprotein receptor 1 (ASGR1), facilitating the uptake of nanoparticles by hepatocytes. Therefore, certain measures need to be taken to minimize their effects. Reprint from [200] with a permission from Springer Nature

Liposomes are normally structured such that the water core can encapsulate hydrophilic drugs while the lipid bilayer can protect hydrophobic drugs. The core chamber of the liposome protects the medications from the external environment as they travel through the circulatory system of a person [216]. Based on their size and the number of bilayers, liposomes may be divided into two categories: unilamellar vesicles and multilamellar vesicles. Both the loading quantity and the half-life of medicines are affected by the size and number of bilayers (MLV). Little SUVs and large SUVs are both types of unilamellar vesicles (LUV) [13, 216]. The structure of multilamellar liposomes resembles that of an onion. On the other hand, multilamellar concentric phospholipid spheres separated by water molecules may be created by the formation of multiple unilamellar vesicles inside other vesicles [92]. According to the results of extensive research on nanocarriers, modern liposomes exhibit a variety of distinguishable qualities and properties, and as a direct consequence, new applications based on liposome materials have emerged [92]. Three major issues have been uncovered and addressed through the process of developing liposomes. The research community has been struggling to overcome biological hurdles and slow the rapid clearance of their results. As was said previously, one of the biggest technical hurdles confronting nanocarriers has always been getting past biological barriers [57]. Nanoliposomes are protected by cells of the human body's mononuclear phagocyte system (MPS), which are mostly located in the liver and spleen. Liposome membrane modification is a crucial method for increasing their stability. Coating the membrane with molecules like proteins, peptides, polymers, and other sorts of molecules may increase the half-lives of liposomal substances. This makes escaping the MPS system much easier [217]. For obvious reasons, these liposomes were given the moniker "stealth." A polyethylene glycol conjugated liposome was shown to have a longer half-life when compared to other modified liposomes. Based on these results, PEG-liposomes containing doxorubicin (commonly known as DOX) were utilized to treat Kaposi's sarcoma in HIV patients. Drug loading and controlled release of liposomes are only two of the many important considerations that must be made during the design of liposome nanocarriers [90, 102]. Drug bioavailability has a role in how well cancer treatment works. Because DOX liposome bioavailability is lower than that of free DOX, designers of liposomes should work to improve bioavailability [169]. The bioavailability of free DOX is greater. Liposomes have several applications, but controlled release and simultaneous administration are two of the most significant [90, 102]. Chemical treatments, metals, gene agents, and others have been combined to create chemotherapeutic cocktail drugs. Figure 15-A illustrates the pathway of a nanoparticle within the human body after being injected intravenously.Overactivation of specific signaling pathways is thought to contribute to the development of cancer, and drugs that interfere with these pathways are used to treat the disease [167]. The study showed that synergistic effects contributed to an increase in the cytotoxic impact by loading a novel PEGylated liposomal with ncl-240 and cobimetinib, both of which are small-molecule inhibitors of the phosphoinositide 3-kinase/mammalian target of rapamycin (PI3K/mTOR) pathway and the mitogen-activated protein kinase/extracellular signal-regulated protein Innovative liposomal nanocarriers containing irinotecan and floxuridine have been shown to be very successful in the treatment of advanced solid tumors [218]. Due to its complex multilayer structure, a single bilayer of a special liposome was able to effectively carry up to 3500 siRNA molecules, and the liposome also carried the delivery of DOX. This enhanced the efficacy of DOX and led to a decrease in the size of the tumor mass in the breast cancer patients being treated. Both triggered release and target approaches are the subjects of much study at present [219]. To avoid pharmaceutical waste, liposomes might be designed to release their contents exclusively in cancerous regions, where the extracellular pH is somewhat lower than in healthy tissue. This is because malignant tissues typically have an extracellular pH value of 6.8 to 7.0. Carboxymethyl chitosan (CMCS) was coated on the surface of the cationic liposome (CL) that was preloaded with sorafenib (Sf) and siRNA (Si), giving it the pH-sensitive characteristic [115]. The results of the experiments showed that sorafenib release was aided and cellular absorption was increased at a pH of 6.5. In addition to the pH-sensitive property, liposomes may be made with a range of responsive qualities depending on the tumor microenvironment (TME) and the characteristics of the drug. Among them are the reactions to oxygen radicals, enzymes, and light [220]. As a word, "tumor microenvironment" describes the surrounding conditions that foster tumor development. The tumor microenvironment (TME) promotes tumor growth, invasion, migration, angiogenesis, and inflammatory processes and is associated with drug resistance. Tumor microenvironment characteristics include EPR presence, hypoxia, acidosis, substantial angiogenesis, and tumor-associated immune cells that aid the immune system in avoiding cancer cells (TME) [221]. In general, liposomes' useful properties include their ability to protect their cargo from enzyme degradation, as well as their low toxicity, biocompatibility, flexibility, high biodegradability, and lack of immunogenicity. Short shelf life, low encapsulation efficiency, unsatisfactory stability, rapid removal by MPS, cell adsorption, and intermembrane transfer are only some of the issues that prohibit liposomes from being widely used. SLNs, for instance, may be anywhere from 1 to 100 nm, placing them in the category of colloidal nanocarriers [222]. Due to the extreme size constraints, SLNs are considered "zero-dimensional" nanomaterials. That's because, on the nanoscale, they're at least one dimension different from similarly sized nanomaterials [178, 179]. Solid lipid nanoparticles (SLNs) are a kind of liposome that lack the liquid components of liposomes and are instead composed of solid lipid, an emulsifier, and water. The constituent parts of SLNs are listed below. Lipides of many different types are used in SLNs, from partial glycerides and triglycerides through fatty acids, waxes, steroids, and PEGylated lipids SLNs [99]. When comparing the structure and function of SLNs with regular liposomes, there are certain parallels and differences. It is interesting to note the parallels between the lipidic membrane and the transport role of chemical treatments. Certain SLNs lack a continuous bilayer, instead generating a micelle-like structure in which drugs are contained in a non-aqueous core [99]. When compared to traditional liposomes, which are composed of lipid bilayers surrounding an aqueous pocket, they are monolayers. Compared to liposomes, SLNs are more stable and have a longer release time. Also, their lipid components are stable at body temperature [99, 222]. Despite this, SLNs have a few downsides, such as a high gelation propensity that can't be predicted and a low integration rate that comes from the molecules' crystalline form. There has been a significant increase in the number of liposomes and SLN that have been modified to serve as NLC carriers during the last two decades [29, 99, 222]. The building blocks of NLCs are a core matrix filled with a combination of solid and liquid lipids. This is done so that the NLCs may maintain their natural protective function, biocompatibility, and non-immunogenicity while also increasing their stability and loading capacity. Many different routes of administration exist for NLCs, such as oral, intravenous, inhalational, and topical (through the eye). Many of the chemical compounds used in cancer therapy are lipophilic, which has sparked a lot of interest in NLCs in recent years [223]. Figure 15-B illustrates the significance of lipid nanoparticles as an established technology for delivering genetic drugs to the liver.

### Nanoemulsions

Nanoemulsions are a kind of colloidal nanoparticle comprised of an aqueous phase, emulsifying agents, and oil. The typical range of nanoemulsion size is between 10 and 1000 nm. Nanoemulsions are often used as medication nanocarriers [117]. Nanoemulsions are spherical, solid particles that are often negatively charged and have an amorphous, lipophilic surface [223]. Due to their nature as heterogeneous mixtures, nanoemulsions can be formulated in three common configurations: (a) water in oil nanoemulsion systems, in which water is dispersed in an aqueous medium; (b) oil in water nanoemulsion systems, in which oil is dispersed in an aqueous medium; Optical clarity, thermodynamic stability, a large surface area, easy production, biodegradability, and an ideal drug release profile are only a few of these advantages. Recent years have seen much study of membrane-modified nanoemulsions [117]. Co-delivery via nanoemulsions is one strategy for improving both bioavailability and therapeutic efficacy. After a battery of studies, it was shown that a NE drug carrier system including spirulina polysaccharides and PTX has the ability to boost PTX's anti-tumor impact by modulating immunity through Toll-like receptor 4/nuclear factor kappa B (TLR4/NF-B) signaling pathways. Using the medications temozolomide, rapamycin, and bevacizumab, a nanoemulsion system was designed to successfully treat metastatic melanoma. Melanoma cells were more sensitive to parenteral therapy, and tumor recurrence, migration, and angiogenesis suppression were all improved [49, 50]. In vitro human and animal cell models were used to show these results. Nanoemulsions may find use in immunotherapy thanks to their ability to be loaded with targeted immune-stimulating moieties. In order to keep the cytokine interferon gamma (IFN-) stable for three months, it was encapsulated in a customized nanoemulsion that could withstand extreme temperatures. Testing showed that this NE decreased the survival of MCF-7 human breast cancer cells and boosted the activity of phagocytes, suggesting it may have a positive function in the treatment of cancer [106]. One use of NE that has seen a surge in attention is as a strategy for avoiding MDR. The ABC transporters, or ATP-binding cassette transporters, play a role in multidrug resistance (MDR) in cancer cells. Medications fail to work in cancer patients due to the expression of MDR transporters, which are encoded by ABCs [115]. The first ABC transporter was identified as P-glycoprotein (P-gp). The drug efflux pump expressed by the ABC1 gene may expel the anticancer drugs colchicine, vinblastine, etoposide, and paclitaxel (PCX) [224]. To overcome this obstacle, Meng and his colleagues developed a novel nanoemulsion that administers both baicalein and paclitaxel at once. Co-encapsulation of these two drugs boosted oxidative stress, leading to an effective strategy for enhancing cell sensitivity to paclitaxel [117]. For example, one study found that baicalein-paclitaxel NE was more effective against tumors than standard paclitaxel preparations in an in-vivo setting. The study found that the activity of caspase-3 was raised in MCF-7/Tax cells, whereas the production of reactive oxygen species (ROS) and glutathione (GSH) in the cells was reduced. These studies highlight the potential advantages of using NEs designed specifically for the treatment of MDR. Despite the advantages that NEs may provide in theory, putting them into practice is challenging [99]. Production of NEs often requires harsh conditions, including high temperatures and pressures. As a result, not all raw materials may be used for NE projects. This is one of the obstacles that must be conquered before NEs can be used in commercial production on a significant scale [121]. Due to the need for expensive high-energy equipment like homogenizers and microfluidizers, the cost of creating NE is much greater than that of more conventional formulations. In order to determine whether or not NE is safe for use in humans, we need to do extensive research on the interactions between the drug's numerous components and the metabolism of NE in the body, which we cannot do without first learning more about the chemistry involved in its production [117, 121].

Different emulsion compositions have been tailored for specific cytokines and applications [225]. For example, the nanoemulsion designed for Interleukin-6 (IL-6) boasts a high encapsulation efficiency of 90%. It has a relatively moderate particle size of 120 nm and a negative zeta potential of -25 mV, indicating good stability [226, 227]. The storage condition at 4 °C for 6 months is suitable for maintaining stability, and the release kinetics indicate that 20% of the cytokine is released within 24 h. This nanoemulsion appears promising for anti-inflammatory therapy [228, 229]. Another noteworthy formulation is for Tumor Necrosis Factor-alpha (TNF-α). While it has a slightly lower encapsulation efficiency of 85%, the particle size is larger at 150 nm, and the zeta potential is -20 mV. It is stored at room temperature for 3 months, indicating stability under ambient conditions [230]. The release kinetics show a controlled release of 15% over 48 h, making it a candidate for targeted cancer therapies [227]. Additionally, the nanoemulsion designed for Interferon-gamma (IFN-γ) demonstrates impressive encapsulation efficiency at 95% [228, 229]. With a small particle size of 80 nm and a zeta potential of -30 mV, it is well-suited for potential autoimmune disease therapy. The extended storage at -20 °C for one year ensures long-term stability, and a slow release rate of 5% after 72 h suggests controlled cytokine delivery. These formulations cater to various applications, such as immune modulation, anti-inflammatory therapy, and cancer targeting, highlighting the versatility of nanoemulsions for delivering encapsulated cytokines [226, 227]. The selection of emulsifiers, lipid phases, and aqueous phases, along with specific storage conditions, plays a crucial role in optimizing the stability and release kinetics of these nanoemulsions, ensuring their efficacy in diverse biomedical applications. Researchers and practitioners can reference this table to choose the most suitable nanoemulsion formulation for their specific needs in cytokine delivery and therapy [225].

### Dendrimers

Dendrimers are a class of macromolecules distinguished by their hyperbranched and tailored structures. The most noticeable characteristics of dendrimers are their highly branching and easily modifiable surfaces [43]. These dendrimer polymers normally have a diameter of between 1 and 10 nm, while some very large dendrimers may reach 14–15 nm in size. Dendrimer molecules have a core that encapsulates theragnostic medicines in a noncovalent fashion, a dendritic internal structure, and a functional surface group-conjugated outside surface [15]. Many dendrimers have been developed for the purpose of cancer therapy. These include polyamidoamine (PAMAM), polypropylenimine (PPI), polyethylene glycol (PEG), bis-MPA (2,2-bis(hydroxymethyl) propionic acid), 5-ALA (5-aminolevulinic acid), and tetraethyleneamine (TEA) (triethanolamine) [15]. Dendrimers' unique structure provides a number of benefits over more typical nanomaterials. The benefits include better solubility and bioavailability of hydrophobic medications; molecular weight control; flexible branching; a low polydispersity index; and a narrow molecular size distribution. Because of their capacity to form compounds with nucleic acids, dendrimers are promising candidates for use as efficient nanocarriers of nucleic acids, especially cationic dendrimers with positively charged surfaces [99]. Two dendrimers that have seen much study and have several potential applications are PAMAM and PPI. With fluorescence imaging as the driving force, a PAMAM dendrimer/carbon dot nanohybrid was designed to simultaneously accomplish MDR control and cancer cell monitoring. During production, two separate complexes emerged. The first part was CDs/DOX, a molecule made up of blue-emitting carbon dots (CDs) and the anticancer drug DOX via non-covalent interactions [15, 99]. A second portion, designated G5-RGD-TPGS, included generation 5 (G5) PAMAM dendrimers specific for the cyclic arginine-glycine-aspartic (RGD) peptide and the drug efflux inhibitor d-alpha-tocopheryl polyethylene glycol 1000 succinate (TPGS). We employed electrostatic attraction to join the two components that would ultimately form a nanohybrid system loaded with two drugs [48]. In vitro fluorescence was generated by the luminescence of CDs, and targeting specificity was generated by the presence of RGD ligands, which target v3 integrin receptors that are overexpressed in cancer cells. The results showed that TPGS significantly impeded the expansion of cancer cells. Dendrimers, with their potential for co-delivery, may also be used to distribute chemicals that have no obvious chemical relationship [202]. DOX is often used to treat cancers of the colon. The apoptotic pathway requires TRAIL, or tumor necrosis factor-related apoptosis-inducing ligand. Both death receptors 4, and 5, or DR4 and DR5, are overexpressed in many types of cancer cells, and TRAIL can bind to both of them [219]. The Pishavar team encapsulated plasmids for both DOX and TRAIL in a dendrimer nanocarrier, producing a nanocarrier with more anticancer effects than modified carriers carrying DOX or TRAIL alone. A PAMAN nanocarrier based on dendrimer was developed to treat liver cancer cells more effectively. Even though unmodified PAMAN dendrimers have problems like low transfection efficiency, poor cell internalization, and unstable encapsulation, the nanomaterial's competitive contrast properties show that it has a lot of potential in combination therapy [231].

### Nano-scale carbon materials

For example, there are many types of carbon nanomaterials (CNMs) that may be further subdivided into subgroups based on the presence or absence of other elements besides carbon. CNMs are used in many industries and medical fields because of their superior electrical, thermal, optical, and mechanical properties [208]. When compared to nanoparticles made from metal, CNMs are thought to be safer and more biocompatible for use in cancer diagnostics. CNMs may load chemical treatments through stacking or hydrophobic interactions because of their inherent hydrophobic property [232]. This makes it possible for CNMs to serve as reliable medication delivery systems. Numerous studies have focused on the potential of carbon nanomaterials for use in cancer treatment, including graphene, fullerene, carbon nanotubes (CNTs), carbon nanohorns (CNHs), carbon quantum dots (CQDs), and graphyne (GDY) [208, 232]. Despite their shared carbon-based constituents, these nanomaterials display a wide range of morphological forms, physical features, and functional applications. Graphene, or sp2-hybridized carbon, is a two-dimensional substance that consists of a single layer of carbon atoms [233]. Because of how it's built, it's capable of some truly impressive mechanical and electrical feats. This is in addition to the fact that it has been the focus of a great deal of research in the realm of biological applications, such as the prevention and treatment of cancer. Graphene-based nanomaterials may be classified into four main types according to their chemical composition, structural arrangement, and physical properties: single-layer graphene; multi-layer graphene; graphene oxide (GO); and reduced graphene oxide (rGO) [234]. Graphene's optical transparency, chemical inertness, high density, molecular barrier-forming properties, and high hydrophobicity are only a few of its remarkable electrochemical and mechanical properties. As its name suggests, graphene only has two dimensions. Graphene's high planar surface permits a greater drug-loading capacity, and its thermal conductivity (5000 W/mK) is also rather remarkable [170]. Graphene's anti-cancer capabilities come from both of these factors. However, poor solubility and the aggregation of nanosheets generated by graphene in solution are induced by van der Waals pressures and a-b stacking interactions. This significantly increases the difficulty of producing graphene and also increases the toxicity of graphene [204]. In light of these drawbacks, scientists have been on the lookout for nanomaterials based on graphene that are both more bioavailable and easier to manufacture. It is expected that these nanomaterials will be both easy to create and retain graphene's advantageous properties [173]. Graphene oxide (GO) is a modified form of graphene that has undergone a chemical transformation. Carbonyl (C = O) and epoxy (C–O–C) groups locate on the basal plane of graphene, whereas functional oxygen groups like carboxyl (-COOH) and hydroxyl (C–OH) locate towards the edge of graphene, forming a typical GO molecule. The notation for the GO derivative in its reduced form is rGO [166]. When compared to graphene, GO and rGO provide greater properties for usage in biological applications. Defective oxygen-bound sp3 carbon atoms exhibit strong hydrophilicity, which aids in the development of colloidal dispersions in aqueous solvents that are very durable against van der Waals hydrophobic interaction-induced aggregation [174]. Meanwhile, the nanosheets' hydrophilic functional groups on the GO's surface make them a versatile substrate for conjugating various substances. This has a great deal of potential for the diagnosis and treatment of cancer, as well as for other diseases that need focused treatment [216]. Table 9 highlights the various nanocarrier-based imaging agents used for cancer diagnosis, each with their respective strengths and weaknesses.

**Table 9:** Nanocarrier-based imaging agents for cancer diagnosis

**Imaging Modality**	**Target Biomarker**	**Contrast Agent Type**	**Sensitivity**	**Specificity**	**Clinical Application**	**Advantages**	**Disadvantages**	**References**
**Magnetic Resonance Imaging (MRI)**	Vascular Endothelial Growth Factor Receptor (VEGFR)	Superparamagnetic Iron Oxide Nanoparticles (SPIONs)	High	High	Tumor detection, Angiogenesis imaging	Non-invasive, High spatial resolution, Multiparametric imaging	Limited target specificity, High cost	[232]
**Positron Emission Tomography (PET)**	Epidermal Growth Factor Receptor (EGFR)	Radiolabeled Gold Nanoparticles	High	High	Early diagnosis, Lymph node metastasis detection	High sensitivity, Quantitative imaging, Non-invasive	Radiation exposure, High cost	[232]
**Computed Tomography (CT)**	Prostate-Specific Membrane Antigen (PSMA)	Iodine-based Contrast Agents	High	High	Prostate cancer diagnosis, Lymph node and bone metastasis imaging	Rapid imaging, High spatial resolution, Multiplanar imaging	Ionizing radiation, Limited soft tissue contrast	[232]
**Optical Imaging**	Cancer Cell-Specific Aptamers	Fluorescent Nanoparticles	High	High	Intraoperative imaging, Tumor margin detection	High target specificity, Real-time imaging, Low cost	Limited tissue penetration, Autofluorescence	[174]
**Ultrasonography**	Tumor-Associated Glycoprotein 72 (TAG-72)	Microbubbles	Moderate	Moderate	Ovarian cancer detection, Lymph node metastasis imaging	Real-time imaging, No radiation exposure, Cost-effective	Operator dependence, Limited tissue penetration	[224]

**Fig. 16:**
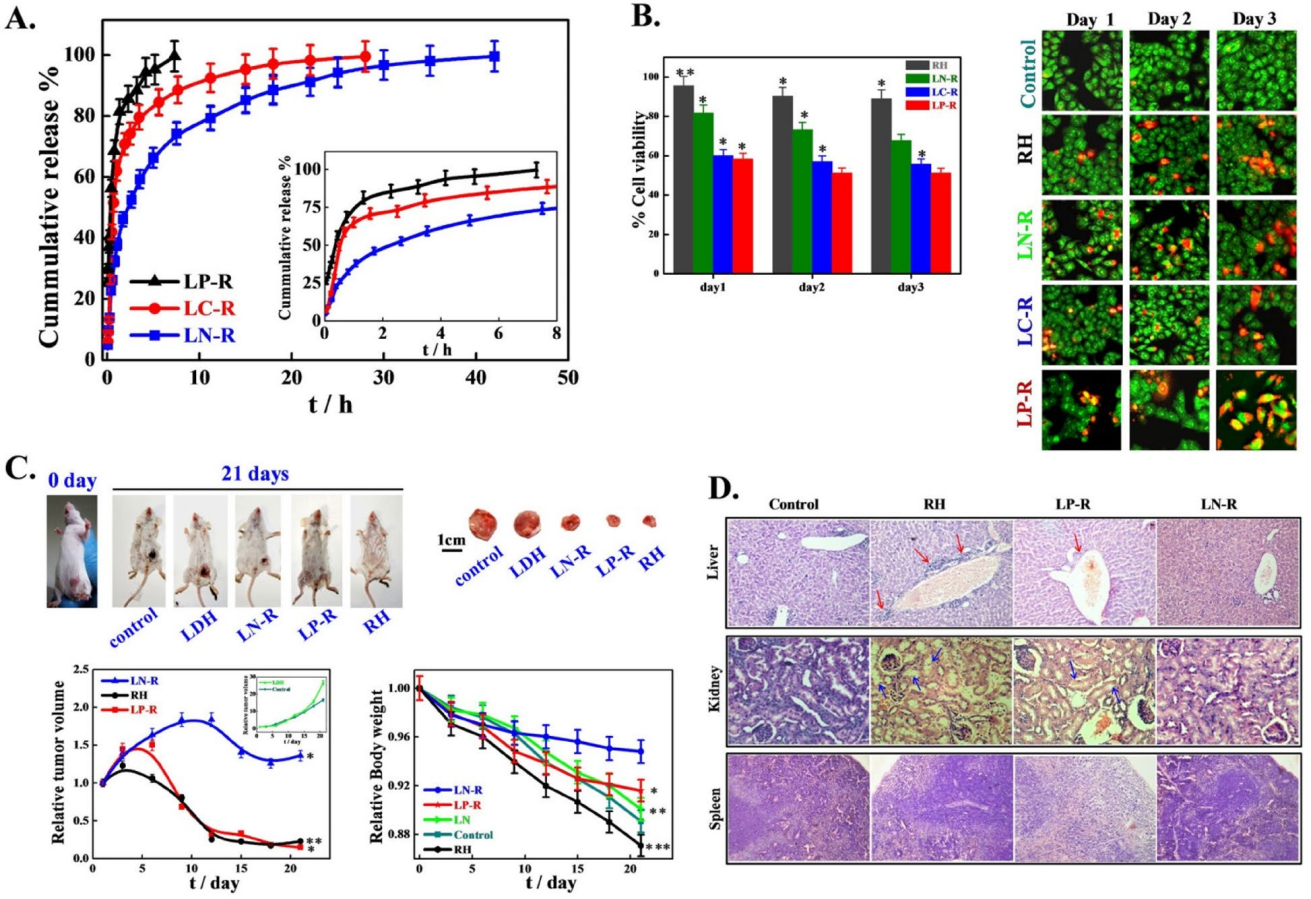
The utilization of layered double hydroxides to control the release of drugs in both in vitro and in vivo settings and their consequent effects. **A** In vitro drug release profiles for three different drugs intercalated LDHs- nitrate, carbonate, and phosphate (LN-R, LC-R, and LP-R respectively) are displayed, along with an inset figure showcasing their release pattern within the first 8 h. **B** The cytotoxicity of the free drug and drug intercalated LDHs against HeLa cells at various time intervals is demonstrated. **C** The antitumor effect and systematic toxicity of pure RH and drug intercalated LDHs are shown in comparison to the control group in an in vivo setting. **D** Finally, the histological analysis of liver, kidney, and spleen of tumor-bearing mice treated with control (saline), pure RH, LN-R, and LP-R are illustrated. Reprint from [180] with a permission from Springer Nature

Graphene's direct immunogenicity toward the immune system sets it apart from other nanomaterials, and its lateral size can be controlled to alter the level to which it stimulates the immune system in vitro and in vivo. In 2011, scientists found evidence that the immune system responds directly to graphene due to its immunogenic properties [57]. The potential of graphene to excite macrophages and dendritic cells, two of the most vital components of the human immune system, has led researchers to believe that it may be effective in the treatment of cancer [110]. The effects of GO nanosheets, designed for use in hyperthermia cancer therapy, on the activities of macrophages and lymphocytes were studied by researchers led by Feito and colleagues. Based on these results, we may conclude that the 6-armed GO (6-GOs) significantly increased tumor necrosis factor alpha (TNF-) production by RAW-264.7 macrophages without altering IL-6 or IL-1 levels [210]. First generation splenocytes were exposed to 1-GOs and 6-GOs in the presence of concanavalin A, lipopolysaccharide, and anti-CD3 antibody. This led to considerable dose-dependent cell growth and a lowered IL-6 level, suggesting the inherent mild inflammatory qualities of GOs, which are beneficial for hyperthermia cancer treatment. Graphene's potential to inhibit tumor cell proliferation has also been revealed. Burnett found that when both hFOB1.19 normal osteoblast and human osteosarcoma (OS) cells were treated with GO, the apoptotic rate of the OS cells was much higher [53]. Human cells were used in the OS. Significant modifications in cytotoxicity against OS, reductions in Nrf-2 and ROS levels, and alterations in cytomorphological features were all brought about by GO [235, 236]. To the average person, (CSCs) are a kind of cancer cell with the ability to self-renew and a high tumorigenic potential. As a result of their interactions with the TME, CSCs have been linked to the progression of MDR. The elimination of CSCs is a potential therapeutic strategy for preventing cancer. It is speculated that GO may specifically target CSCs while sparing healthy cells [237]. Additionally, it has been shown that GO can induce CSC differentiation and prevent the formation of tumor spheres in a variety of cell lines, including breast, ovarian, prostate, lung, pancreatic, and glioblastoma cells, by inhibiting several key signaling pathways, including WNT, Notch, and STAT-signaling. The scientists used the phrase "differentiation-based nano-therapy" to explain this phenomenon [236]. However, there have only been a few studies conducted over the course of the last several years, so it's feasible that we need more information. Additional research on graphene's effect on the immune system and its direct anti-CSC activities is required. Graphene's high surface-to-volume ratio and abundance of oxygen-containing branches make it an ideal platform for drug delivery, photodynamic treatment (PDT), and photothermal therapy (PTT). The Ac-(GHHPH)4-NH2 peptide sequence was grafted onto GO to form a GO-peptide hybrid via irreversible physical adsorption.The anti-angiogenic domain of histidine-proline-rich (HPRG) Human neuroblastoma (SH-SY5Y) cells, human retinal endothelium cells (PC-3) cells, and prostate cancer (PC-3) cells were used to test the hybrid nanomaterial (primary HREC) [236, 237]. The results showed that this GO-peptide nanoassembly was able to inhibit cell migration, reduce prostaglandin-mediated inflammation in PC-3 cells, and reduce toxicity in prostate cancer cells. Due to the limitations of liposomal doxorubicin (L-DOX) in the treatment of breast cancer, a novel DOX-loaded GO nanocarrier was created to improve its nucleation and internalization [219]. Increased anticancer activities were seen when GO-DOX was added to breast cancer cell lines. When linked to the cell plasma membrane, GO-DOX was shown to cause a massive release of DOX within the cell, which contributed to its remarkable efficacy. Live-cell confocal imaging and fluorescent lifetime imaging microscopy allowed for this finding. There is mounting evidence that GOs and rGOs may target hypoxia and abnormal angiogenesis in the tumour tissue microenvironment (TME) [219]. GOs and rGOs find widespread use in PDT and PTT. An allotrope of graphene, GDY features two acetylenic linkages per unit cell. The carbon chains joining the hexagonal rings are made twice as long as a result of these junctions. GYD is far more bendable than graphene or graphyne as a result of this. Research using GYD as a drug delivery platform for photothermal/chemotherapy combinatorial techniques in cancer diagnostics has increased during the last three years [236, 237]. Molecules called fullerenes are constructed from several all-carbon building blocks. Depending on their structure, fullerenes may take on the form of hollow spheres, ellipsoids, or tubes. C60, C70, and C82 are all examples of common fullerenes. By adding metal atoms to a fullerene, a metallofullerene may be made. Typically, Group III transition metals or lanthanides make up the metal atoms contained inside the fullerene [208, 232]. Due to the possibility of intra-fullerene electron migration from an encased metal atom to the fullerene cage, metallofullerenes may be used as a material for magnetic resonance imaging. The qualities that give fullerenes their ability to scavenge free radicals also give them the ability to act as antioxidants [93]. Among nanomaterials, fullerene stands out for its extraordinary PDT and PTT properties. Calculations of photothermal efficiency were shown to be inaccurate due to a number of factors, including the concentration of nanoparticles and the length of time that the laser was shining on the sample, as determined by research by Chen et al. They also found that polyhydroxy fullerenes had a photothermal conversion efficiency of 69% [238]. The fact that fullerenes' photothermal reaction was unaffected by repeated laser irradiation and that their structure was retained throughout the process made them ideal candidates for use in photothermal therapy. Near-infrared (NIR) light-harvesting fullerene-based nanoparticles (DAF NPs) were tested for use in PA imaging-guided synergetic tumor photothermal and photodynamic treatment (PDT) [238]. When compared to fullerene and antenna nanoparticles, DAF NPs were much more effective in producing reactive oxygen species and heat (DA NPs). In vitro and in vivo studies suggest that the synergistic combination of PDT and PTT in DAF NPs might effectively reduce the formation of malignancies. Chemical drug delivery using fullerene has been attempted using PDT and PTT [208, 232]. This was accomplished at a nanocarrier's worth of capacity. Graphene is folded up into cylindrical tubes called CNTs. Sp2-hybridized carbon atoms form the tubes. CNT sizes can vary widely, from 1 nm up to several micrometers. Based on the number of layers formed inside the CNT, it is possible to classify the CNT as either single-walled or multi-walled (MWCNTs). Unfortunately, CNTs are poisonous and have little water solubility, among other drawbacks. Many studies on surface functionalization and material changes have been performed to solve the aforementioned difficulties and boost the bioavailability of CNTs. As carbon-based nanomaterials (CNTs) may interact with immune cells and activate immunological responses, they may improve immunity and restrain tumor growth [239, 240]. Carbon nanotubes (CNTs) are well-studied nanocarriers that are largely believed to be efficient PDT and PTT vehicles. Sundaram and his team used photodynamic treatment on colon cancer cells after combining single-walled carbon nanotubes (SWCNTs) with hyaluronic acid (HA) and chlorin e6 (Ce6) (PDT). Changes in cell appearance, as measured by microscopy, LDH cytotoxicity, and induction of cell death, were seen after 24 h. According to the results of the study, the newly produced chemical enhanced the PDT's efficiency [106, 107]. The PTT efficiency of another NIR active photothermal agent, CNTs-PAMAM-Ag2S, was shown to be quite high. When exposed to laser irritation at a wavelength of 980 nm, the research showed that the photothermal efficacy of this complex was higher than that of copper-based and well-known gold photothermal agents [88]. Moreover, the compound has shown excellent stability against photo-bleaching and photo-corrosiveness, indicating that the novel nanoagent may have use in PTT. A lot of effort has gone into studying the efficacy of carbon nanotube (CNT)-based drug delivery systems (DDSs), including DOX, PTX, and cis platinum (CDDP). Carbon nitrides (CNHs) are a kind of carbon allotrope [106, 107]. While CNTs are generally 100 nm in length, larger spherical superstructures may be formed with sp2 hybridized carbon atoms with a diameter of between 2 and 5 nm. Similar to CNTs, CNHs are insoluble and need surface modifications to serve as nanocarriers in human tissue. Adding organic species to the outside skeleton, or forming conjugate planar aromatic molecules by electrostatic association or stacking interactions, are two potential approaches. CNHs were used in the creation of DDS that include combination features due to their capacity for both drug loading and photothermal responses [208, 232]. Yang and coworkers developed a single-walled CNH system loaded with two different chemotherapeutic agents. mPEG-PLA altered SWNHs through hydrophobic-hydrophobic stacking interactions as well as -stacking interactions. Both cisplatin and doxorubicin (DOX) were loaded onto nanohorns but in separate compartments [241]. The nanocarrier showed a pH-dependent releasing capacity in addition to a loading ability and an efficient photothermal ability. Findings indicated that both primary breast tumors and lung metastases had been successfully eradicated [49, 50]. CNHs may be tailored with specific targeting molecules for use in target chemical therapy to address a wide range of medical issues. A cisplatin-loaded CNH fused to a monoclonal antibody (mAb) D2B that targets prostate specific membrane antigen (PSMA) + prostate cancer cells has been demonstrated to be more effective and selective than other hybrids in killing PSMA + prostate cancer cells. The toxicity and side effects of CNMs have been extensively studied because of their prevalence in cancer therapy. Serum protein adsorption, hemolysis, cytotoxicity, and immunotoxicity have all been linked to GO and rGO (93) [208, 232]. The large surface area of GO and rGO makes them candidates as substrates for the adsorption of proteins in a biological setting. As proteins adsorb onto the nanomaterial, the intended function of the nanomaterial may be compromised, and blood vessel blockage may result. According to in vitro and animal studies, the toxicity of nanomaterials may depend on factors such as the quantity of GO and rGO present and the size of the particles [90, 102]. One study found that cells with large amounts of hydrophobic rGO on their membranes were more likely to undergo significant ROS stress, which may lead to cell death. In vivo studies have shown that CNTs are able to induce pathophysiology similar to that of mesothelioma, including chronic inflammation, the formation of granulomas, and fibrosis. Yan et al. summarized the elements affecting CNT-induced toxicity in their investigation. Surface modification, aggregation, concentration, CNT size, and CNT shape are all relevant variables. They also outlined potential CNT accumulation areas after anticancer medication withdrawal. However, it is still unclear which aspect of CNMs plays the most essential function and what the actual processes of cellular toxicity induced by CNMs are, despite the wealth of data acquired from a broad range of cells and animals [239–241]. Figure 16 depicts the use of layered double hydroxides (LDHs) to regulate the release of drugs in both in vitro and in vivo scenarios and their subsequent effects. The figure displays in vitro drug release profiles for three different LDHs intercalated with nitrate, carbonate, and phosphate (LN-R, LC-R, and LP-R, respectively). The inset figure showcases their release pattern within the first 8 h. The cytotoxicity of free drugs and drug intercalated LDHs against HeLa cells is also demonstrated at various time intervals. Additionally, the figure shows the antitumor effect and systematic toxicity of pure RH and drug intercalated LDHs compared to the control group in an in vivo setting. Finally, the histological analysis of liver, kidney, and spleen of tumor-bearing mice treated with control (saline), pure RH, LN-R, and LP-R is illustrated.

### Dots on a quantum scale

Due to their distinctive optical and electrical capabilities, quantum dots are being extensively studied as potential biological imaging probes. The most frequent usage for these nanometer-scale semiconductor crystallites is to improve the efficacy of fluorescent markers used in biological imaging, although they have many other potential uses [103, 104]. Size and composition are only two examples of the quantum dot's unique optical and electrical properties that allow for wavelength-tunable fluorescence emission from the visible to the infrared, large absorption coefficients, and high brightness levels with excellent photostability. Carbon-based quantum dots include graphene quantum dots (GQDs), nanodiamonds, and carbon dots (CDs) [103, 104]. Bioimaging is where carbon QDs are most often used, and this discipline is where they are most useful for detecting and studying cancer. GQDs are seen as promising nanomaterials in biosensing and cancer therapy due to their better biocompatibility, rapid excretion, and huge surface area that is ideal for molecular conjugation. Building a photoluminescent glycodendrimer system with terminal-cyclodextrin molecules allowed for DOX administration that was both biocompatible and pH-sensitive [176]. In order to create a surface on which PAMAM could grow, GQDs were employed. After being first stimulated by UV light at 365 nm, GQDs and GQDs-PAMAM—CD had their emission spectra recorded. Having the GQDs in there meant it could be used as a photoluminescent imaging agent [15]. The data also showed that it killed cancer cells more effectively than DOX alone. This innovative nanocarrier for targeted therapy takes advantage of the fluorescence-inducing properties of GQDs. They were able to connect folic acid to sulfur-doped graphene quantum dots (FA-SGQDs) by a simple pyrolysis procedure including citric acid (CA), folic acid, and 3-mercaptopropionic acid (MPA) [15, 55, 176]. A blue fluorescence with an emission band at 455 nm was seen after exciting the compound at 370 nm. In addition, a strategy for TA-SGQDs to enter FR-positive cancer cells through a mechanism other than immunogenic FR-mediated endocytosis was identified. In addition to bioimaging and biosensing, researchers were looking at the potential of GQDs for photothermal therapy (PTT) and photodynamic therapy (PDT) [103, 104]. A modified GQD was created that showed strong absorption at 1070 nm in the NIR-II range. The so-called 9 T-GQDs were able to effectively ablate tumor cells and, as a consequence, NIR-II irradiation reduced the development of the tumor because of its uniform size distribution, adjustable fluorescence, and high photothermal conversion effectiveness (33.45%) [57]. This exemplified GQDs' potential in PTT. A carbon quantum dot-based photodynamic-chemotherapy drug delivery device was created. Researchers combined 5-aminolevulinic acid (5-ALA) with a mono-(5-BOC-protected-glutamine-6-deoxy) -cyclodextrin (CQD-glu—CD) moiety, and then conjugated these materials to CQDs loaded with DOX. Radiation at 635 nm (25 mW cm-2) for 15 min also generated reactive oxygen species (ROS) and improved treatment outcomes [176]. The morphology of the MCF-7 cancer cells changed dramatically, and there was significant cytotoxicity as a consequence. CDs and nanodiamonds have both been studied for their potential use in cancer treatment due to their targeted therapy, photodynamic therapy (PDT), cancer imaging, and mediation of antitumor immune properties. In comparison to other carbon-based materials, the study of carbon QDs is still in its infancy. A lack of a standard way to make high-quality QDs and a lack of knowledge about how they work and how they are made are two of the biggest problems with using them in clinical settings [110].

### Nanoscale materials that are magnetic and metallic

Researchers in the fields of bioimaging and drug delivery have focused extensively on metallic nanoparticles due to their unusual optical, magnetic, and photothermal capabilities. Metallic materials may be used in many different applications since they can be conjugated with many different carriers. When it comes to applications, magnetic nanoparticles in MRI are the most prevalent (MRI). An external magnetic field may guide magnetic nanoparticles (NPs) loaded with chemical treatments to cancer cells [20]. This reduces the risk of discomfort associated with conventional chemotherapy. The linked metal particle allows the nanosystem to do both bio-imaging and PTT. Iron oxide nanoparticles (IONPs) were created by enclosing Fe3O4/Ag in gold. The MRI contrast capabilities of IONPs and PTT were revealed to be the result of the presence of a gold shell in the NIR region [166]. Metals are often used in the cancer treatment methods of photothermal therapy (PTT), photodynamic therapy (CDT), and immunotherapy. The CDT is a method of therapy predicated on the Fenton reaction or an analogous reaction. It employs a nanocatalyst. High levels of oxidizing hydroxyl (OH) radicals are generated in a manner similar to photodynamic therapy (PDT), and these toxic OH radicals kill cancer cells by triggering chain reactions with the organic molecules in the surrounding tissue [121]. As a consequence of these processes, DNA, lipids, and proteins are all susceptible to irreversible damage as a consequence. Catalyzing the disproportionation of H2O2 to create OH radicals is accomplished using iron-based nanostructures such as FeS2, Fe2P, Fe3O4, SnFe2O4, and amorphous iron. Near infrared (NIR) triggered materials are crucial to the success of photodynamic treatment (PDT) and photothermotherapy because of the greater depth at which NIR light may permeate tissue compared to visible light and ultraviolet (UV) light (PTT) [59]. When cancer cells are destroyed using photothermal treatment (PTT), heat is produced. It is the reactive oxygen species (ROS), hydroxyl radical (OH), singlet oxygen (1O2), and superoxide (O2) that trigger the cytotoxic reactions in the process of photodynamic treatment (PDT). All gold (Au), copper (Cu), and iron (Fe) are metals that have found medical use. One of the key downsides of these materials is that metallic nanoparticles are toxic. The formation of reactive oxygen species (ROS) and the effect on cell architecture were only two of the processes of metallic NPs summarized by Attarilar et al. Size, shape, dimensionality, and surface charge are all factors in the toxicity of NPs; these factors are also relevant to metallic NPs. Therefore, further research is required before metallic nanoparticles may be employed on human patients [242, 243].

## Methods for cancer therapy

**Fig. 17:**
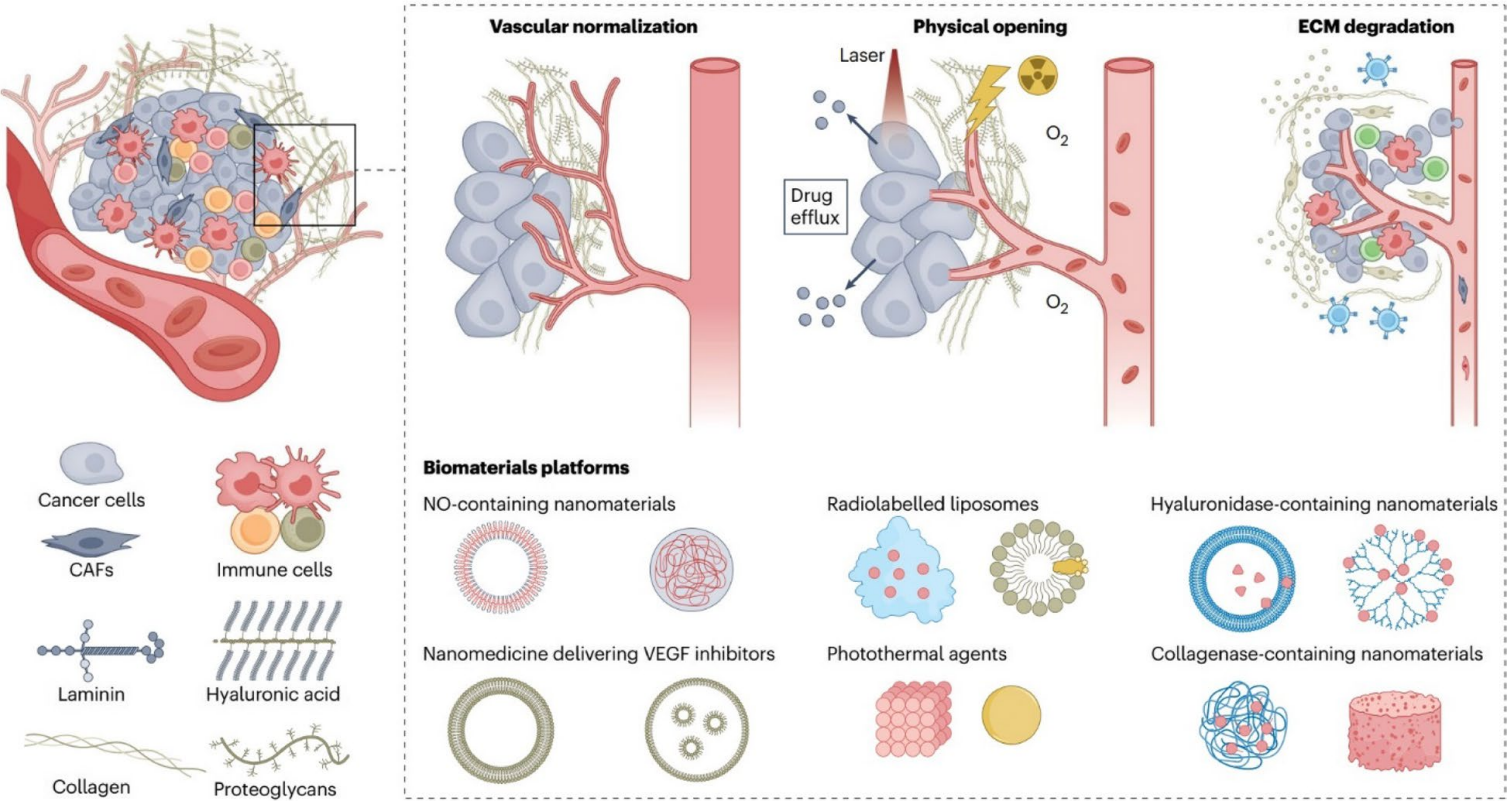
Breaching the tumor barrier physically. Immune cell infiltration can be facilitated by physically disrupting the tumor microenvironment using biomaterials-based instruments that help stabilize the blood vessels. This can be achieved through the use of radiolabeled or photothermal agents, followed by the application of laser or radiation, as well as employing nanomaterials that release enzymes to break down the extracellular matrix (ECM). Reprint from [244] with a permission from Springer Nature

A variety of time-tested approaches to cancer treatment continue to be routinely used today. Most research is focused on tumor cells and the tumor microenvironment (TME), which includes the immune system that is linked to the tumor [171]. Figure 17 illustrates the innovative approach of breaching the tumor barrier physically in order to facilitate immune cell infiltration. By employing biomaterials-based instruments, this method seeks to stabilize blood vessels within the tumor microenvironment, making it more accessible to immune cells. Techniques such as radiolabeled or photothermal agents can be employed, accompanied by the use of laser or radiation, to achieve the desired disruption. Additionally, the strategic application of nanomaterials capable of releasing enzymes aids in breaking down the extracellular matrix (ECM), further enhancing the ability of immune cells to penetrate the tumor. Crucial components within this process include cancer-associated fibroblasts (CAFs), nitric oxide (NO), and vascular endothelial growth factor (VEGF), all of which play significant roles in modulating the tumor microenvironment and enabling a more effective immune response against cancer cells. Table 10 provides a comprehensive overview of various methods used for evaluating the efficacy of nanocarriers in drug delivery. These methods range from in vivo tumor growth inhibition assays, which directly measure the reduction in tumor size over time, to more specialized techniques like surface plasmon resonance and electrochemiluminescence, which focus on molecular interactions and drug detection, respectively. Each method has its own set of advantages and disadvantages, as well as varying degrees of clinical relevance, sensitivity, specificity, and reproducibility. By utilizing a combination of these methods, researchers can gain a better understanding of nanocarrier performance, pharmacokinetics, biodistribution, and impact on cellular and molecular processes, ultimately leading to more effective and targeted cancer therapies.

**Table 10:** Methods for evaluating nanocarrier efficacy

**Evaluation Method**	**Efficacy Metric**	**Sensitivity**	**Specificity**	**Reproducibility**	**Clinical Relevance**	**Description**	**Novelty**	**Advantages**	**Disadvantages**	**References**
**Tumor growth inhibition assay**	Tumor volume reduction	High	High	High	High	Measures the effect of the nanocarrier on tumor growth in vivo	Measures the reduction in tumor size over time, which is a direct indicator of treatment efficacy	Allows for testing of drug efficacy in vivo; can be used to evaluate drug combination therapies	Expensive; may require a large number of animals; can be time-consuming	[45]
**Cellular uptake assay**	Intracellular drug concentration	High	High	Moderate	Moderate	Measures the amount of nanocarrier taken up by cancer cells in vitro	Quantifies the amount of drug delivered to cancer cells; can determine if the nanocarrier is internalized and if drug release occurs	Can be performed quickly and easily; cost-effective	Does not provide information on drug efficacy in vivo; may not reflect tumor heterogeneity	[245]
**In vitro drug release assay**	Drug release kinetics	High	High	High	Moderate	Measures the rate of drug release from the nanocarrier in vitro	Quantifies the amount of drug released over time; can determine if the release rate is sustained and controlled	Provides a rapid and reliable assessment of nanocarrier drug release kinetics	Does not reflect the complexity of in vivo drug release; may not reflect the effect of the tumor microenvironment on drug release	[246]
**Pharmacokinetic analysis**	Drug concentration in blood/tissues	High	High	High	High	Measures the distribution, metabolism, and excretion of the nanocarrier in vivo	Quantifies the amount of drug delivered to the tumor and other organs; can determine the clearance pathway and half-life	Provides valuable information on nanocarrier pharmacokinetics and biodistribution	Expensive; may require large sample sizes; may not reflect tumor heterogeneity	[115]
**Imaging-based analysis**	Tumor contrast enhancement	Moderate	High	High	Moderate	Measures the accumulation of the nanocarrier in the tumor using imaging modalities such as MRI or PET	Quantifies the amount of nanocarrier delivered to the tumor; can determine if the nanocarrier targets the tumor specifically	Provides real-time visualization of the nanocarrier accumulation in the tumor	Requires specialized equipment and expertise; may not reflect the effect of the tumor microenvironment on nanocarrier accumulation	[45]
**Flow cytometry**	Percentage of cells with drug uptake	Moderate	High	High	Low	Measures the amount of nanocarrier taken up by cells in vitro	Allows for high-throughput screening of large numbers of cells; can quantify the proportion of cells that take up the drug	Rapid and quantitative; can distinguish between live and dead cells	May not reflect the in vivo behavior of the nanocarrier; may require specialized equipment and expertise	[115]
**Histological analysis**	Tumor cell apoptosis and necrosis	High	High	High	High	Measures the effect of the nanocarrier on tumor cell death and destruction in vivo	Quantifies the amount of tumor cell apoptosis and necrosis; can determine if the nanocarrier induces tumor cell death	Provides information on the histological effects of the nanocarrier on the tumor	Requires invasive tissue sampling; may not reflect the in vivo behavior of the nanocarrier; may require specialized expertise	[45]
**Proteomic analysis**	Expression of tumor-associated proteins	High	High	High	High	Measures the effect of the nanocarrier on tumor-associated protein expression in vivo	Quantifies changes in protein expression in response to nanocarrier treatment; can determine if the nanocarrier affects specific signaling pathways	Provides information on the mechanism of action of the nanocarrier	Requires specialized expertise and equipment; may not reflect the in vivo behavior of the nanocarrier; may be time-consuming	[233]
**Magnetic resonance spectroscopy**	Metabolic changes in tumor cells	High	High	High	High	Measures changes in tumor metabolism in response to nanocarrier treatment in vivo	Quantifies changes in metabolite levels in response to nanocarrier treatment; can determine if the nanocarrier affects specific metabolic pathways	Provides information on the metabolic effects of the nanocarrier on the tumor	Requires specialized equipment and expertise; may not reflect the in vivo behavior of the nanocarrier	[247]
**Transcriptomic analysis**	Gene expression changes in tumor cells	High	High	High	High	Measures changes in gene expression in response to nanocarrier treatment in vivo	Quantifies changes in gene expression in response to nanocarrier treatment; can determine if the nanocarrier affects specific signaling pathways	Provides information on the molecular effects of the nanocarrier on the tumor	Requires specialized expertise and equipment; may not reflect the in vivo behavior of the nanocarrier	[233]
**Drug resistance assays**	IC50 value or other resistance metrics	Moderate	Moderate	High	Moderate	Measures the sensitivity of cancer cells to nanocarrier-delivered drugs	Quantifies the extent of resistance or sensitivity to the nanocarrier-delivered drug	Useful for identifying the most appropriate nanocarrier-drug combination for specific cancer types	Requires careful optimization and validation; may not reflect the in vivo behavior of the nanocarrier	[115]
**3D tumor models**	Tumor size or volume, viability, and drug distribution	High	High	Moderate	High	Measures the effect of the nanocarrier on tumor growth and viability in a 3D culture system	Mimics the complexity of the in vivo tumor microenvironment; can quantify the drug distribution within the tumor model	Provides a more physiologically relevant system for evaluating nanocarrier efficacy	Can be expensive and time-consuming; may require specialized equipment and expertise	[115]
**Serum cytokine analysis**	Changes in cytokine levels	High	High	High	Moderate	Measures the effect of the nanocarrier on the immune response in vivo	Quantifies changes in cytokine levels in response to nanocarrier treatment; can determine if the nanocarrier modulates the immune response	Provides information on the immune modulatory effects of the nanocarrier	Requires specialized expertise and equipment; may not reflect the in vivo behavior of the nanocarrier	[163]
**Microfluidic platforms**	Drug delivery and efficacy in a microfluidic device	High	High	High	Low	Measures the effect of the nanocarrier on cancer cells and drug delivery in a microfluidic device	Mimics the behavior of the nanocarrier in the human body; can quantify the drug distribution within the microfluidic device	Provides a more accurate and controlled system for evaluating nanocarrier efficacy	Requires specialized equipment and expertise; may not reflect the in vivo behavior of the nanocarrier	[248, 249]
**Optical imaging**	Imaging of tumor size and distribution in vivo	High	High	High	High	Measures the effect of the nanocarrier on tumor size and distribution in vivo	Can quantify the tumor size and distribution in real-time; can be used for non-invasive imaging	Provides a non-invasive and rapid system for evaluating nanocarrier efficacy	Limited by the depth of light penetration into tissues; may not reflect the effect of the tumor microenvironment on nanocarrier distribution	[125]
**Scanning electron microscopy**	Imaging of nanocarrier-cell interaction	High	High	High	Low	Measures the physical interaction between nanocarriers and cancer cells in vitro	Provides a detailed view of the nanocarrier-cell interaction; can determine the cellular uptake mechanism of the nanocarrier	Provides a direct and visual representation of the nanocarrier-cell interaction	Requires specialized equipment and expertise; may not reflect the in vivo behavior of the nanocarrier	[115]
**Surface plasmon resonance**	Real-time analysis of molecular interactions	High	High	High	Moderate	Measures the interaction between nanocarrier and cancer cell molecules in vitro	Can provide real-time analysis of the molecular interactions between the nanocarrier and cancer cells	Provides a highly sensitive and specific method for detecting molecular interactions	May not reflect the in vivo behavior of the nanocarrier; requires specialized equipment and expertise	[250]
**Bioluminescent imaging**	Imaging of tumor size and distribution in vivo	High	High	High	High	Measures the effect of the nanocarrier on tumor size and distribution in vivo	Provides non-invasive imaging of the tumor size and distribution in real-time	Provides a non-invasive and rapid system for evaluating nanocarrier efficacy	Limited by the depth of light penetration into tissues; may not reflect the effect of the tumor microenvironment on nanocarrier distribution	[250]
**Microscale thermophoresis**	Analysis of nanocarrier-protein interaction	High	High	High	Moderate	Measures the interaction between nanocarrier and cancer cell proteins in vitro	Provides a highly sensitive method for detecting the binding affinity between the nanocarrier and proteins	Requires only small amounts of sample; allows for the analysis of protein interactions in a high-throughput manner	May not reflect the in vivo behavior of the nanocarrier; requires specialized equipment and expertise	[248, 249]
**Electrochemiluminescence**	Detection of nanocarrier-delivered drug in plasma or tissue samples	High	High	High	High	Measures the drug concentration in plasma or tissue samples	Provides highly sensitive and specific detection of the nanocarrier-delivered drug	Requires only small amounts of sample; provides rapid results	May require specialized equipment and expertise; may not reflect the in vivo behavior of the nanocarrier	[107]

### Methods that target cancer cells directly

**Fig. 18:**
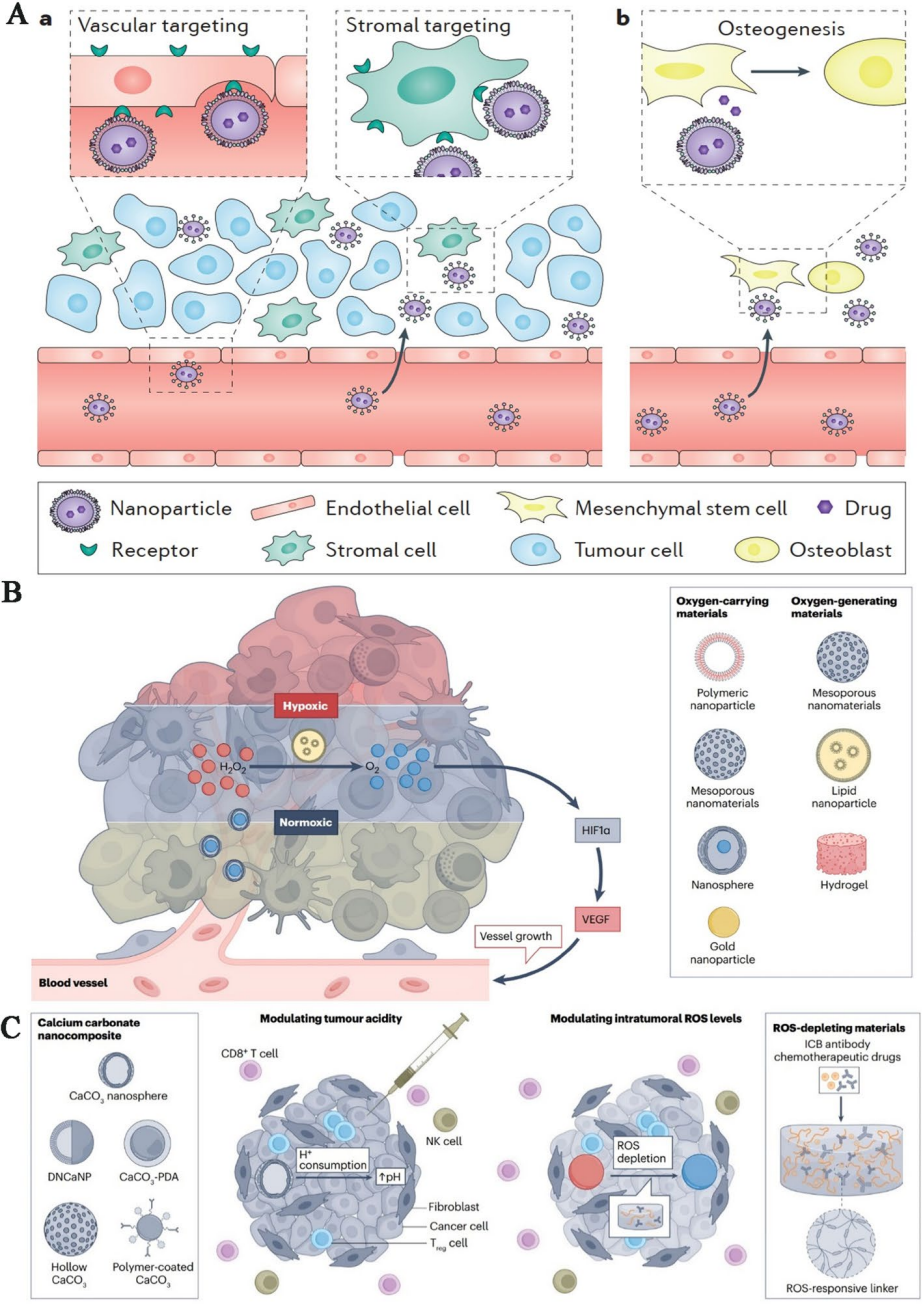
**A** The use of nanoparticles to target the microenvironments of tumors and premetastatic areas. Part A shows how the tumor vasculature or stromal cells in the tumor microenvironment can be targeted. Part B shows targeting of premetastatic microenvironments such as the bone marrow niche, where nanoparticles can be used to enhance bone strength and volume through osteogenic differentiation of mesenchymal stem cells. To achieve cell-specific targeting, nanoparticles can be modified with ligands that bind to specific receptors on the surface of target cells. However, even without targeting ligands, nanoparticles can still be engineered for preferential uptake by these cells. The cells can also take up the payloads released from the nanoparticles that are localized in tumors or premetastatic tissues, even in a non-specific manner**.** Reprint from [177] with a permission from Springer Nature. **B** Manipulating tumor hypoxia using biomaterials. A range of biomaterial-based techniques, such as oxygen production and transportation systems, can be utilized to control hypoxia within tumors and their surrounding microenvironments. Reprint from [244] with a permission from Springer Nature. **C** Utilizing Biomaterials to Decrease Tumor Acidity and Control ROS Levels. A variety of biomaterials-focused approaches, especially those involving calcium-carbonate-based materials, can be introduced into the tumor surroundings to counteract tumor acidity. Reactive oxygen species (ROS) levels can be regulated using oxygen-free radical-absorbing hydrogels. These hydrogels can also serve as vehicles for delivering antibodies and chemotherapy medications. DNCaNP refers to liposome-encapsulated calcium nanoparticles, ICB stands for immune checkpoint blockade, PDA denotes polydopamine, and Treg cell represents regulatory T cells. Reprint from [244] with a permission from Springer Nature

Naturally, eliminating cancer naturally requires therapies that specifically target cancer cells. Nanoparticles (NPs), dendrimers (dNMs), and conjugated nanomaterials (CNMs) may be customized to use EPR in combination with active targeting to enter cancer cells and deliver chemical therapies or biomaterials. These systems rely heavily on antibodies that recognize and bind to antigens that are overexpressed on the surfaces of cancer cells. After being taken up by cancer cells, encapsulated chemical treatments may induce cytotoxicity, whereas encapsulated nucleic acid components may cause cell death. Nucleic acid delivery science has come a long way, which has led to a lot of research into nano-DDS therapies that use exosomes, PNPs, liposomes, and dendrimers to treat cancer [171]. Figure 18 highlights the potential of nanoparticle targeting in the tumour microenvironment and the premetastatic niche. Also, Fig. 18-A illustrates that targeting the tumour vasculature or stromal cells can be achieved using modified nanoparticles with specific ligands that bind to receptors on the surface of these cells. In addition, the figure shows that nanoparticle targeting can be used in premetastatic tissues such as the bone marrow niche, where the osteogenic differentiation of mesenchymal stem cells can be enhanced to increase bone strength and volume. Interestingly, nanoparticles can be engineered to achieve preferential cellular uptake even without targeting ligands. It is worth noting that the payloads released from these nanoparticles can also be taken up by these cells, regardless of whether they are localized in tumours or premetastatic tissues. Figure 18-B illustrates the innovative approach of utilizing biomaterials to manipulate tumor hypoxia, effectively addressing a critical challenge in cancer therapy. By employing various biomaterial-based techniques, such as oxygen production and transportation systems, researchers are able to regulate hypoxia within tumors and their surrounding microenvironments. This fine-tuned control plays a vital role in influencing key factors, such as HIF1α (hypoxia-inducible factor-1α) and VEGF (vascular endothelial growth factor), which are responsible for promoting tumor growth and angiogenesis under hypoxic conditions. As a result, these biomaterial strategies offer the potential to disrupt the tumor's ability to adapt to a low-oxygen environment, thereby enhancing the effectiveness of cancer treatments and improving patient outcomes. Figure 18-C illustrates the use of biomaterials to mitigate tumor acidity and modulate reactive oxygen species (ROS) levels in the tumor microenvironment. This innovative approach focuses on the integration of calcium-carbonate-based materials, which are specifically designed to neutralize the acidic conditions typically associated with tumors. Furthermore, the figure highlights the application of oxygen-free radical-absorbing hydrogels to regulate ROS levels, thus preventing oxidative stress and damage. These hydrogels not only control ROS but also function as carriers for the targeted delivery of antibodies and chemotherapy drugs. Key abbreviations featured in Fig. 3 include DNCaNP (liposome-encapsulated calcium nanoparticles), ICB (immune checkpoint blockade), PDA (polydopamine), and Treg cell (regulatory T cells). The combination of these strategies exemplifies the potential of biomaterials in transforming cancer treatment modalities. Notably, viral vectors like adenovirus and adeno-associated virus demonstrate high transfection efficiency and gene expression levels but may induce immune responses and potential toxicity. Non-viral vectors, such as lipid nanoparticles and polymeric nanoparticles, show promise due to their biocompatibility, while CRISPR-Cas9 and CRISPR-Cas13a systems offer specific and efficient gene targeting. However, long-term safety and potential off-target effects of these systems remain concerns. Electroporation and in vivo electroporation provide non-toxic, non-immunogenic delivery methods but require specialized equipment and expertise. Finally, mRNA-based therapies, such as mRNA electroporation, enable rapid and customizable production, but potential immune response and toxicity must be considered. Each approach presents unique advantages and disadvantages, emphasizing the importance of continued research and development in this field. Table 11 outlines various nanocarrier-mediated gene therapy approaches for cancer treatment, highlighting their target genes, delivery methods, transfection efficiency, gene expression levels, and therapeutic outcomes.

**Table 11:** Nanocarrier-mediated gene therapy for cancer

**Gene Therapy Approach**	**Target Gene**	**Delivery Method**	**Transfection Efficiency**	**Gene Expression Level**	**Therapeutic Outcome**	**Description**	**Novelty**	**Advantages**	**Disadvantages**	**References**
**Viral vectors**	p53	Adenovirus	High	High	Induces apoptosis	Adenovirus is a commonly used vector for gene therapy due to its high transfection efficiency and ability to infect a wide range of cells	The use of adenovirus for p53 gene therapy is a novel approach that has shown promising results in preclinical studies	High transfection efficiency results in high gene expression levels and improved therapeutic outcomes	Adenovirus can induce an immune response, limiting its effectiveness and potential toxicity	[49, 50]
**Non-viral vectors**	siRNA	Lipid nanoparticles	Moderate	Moderate	Downregulates target gene expression	Lipid nanoparticles are a promising non-viral vector for gene therapy due to their biocompatibility and ability to encapsulate and protect nucleic acids	The use of lipid nanoparticles for siRNA delivery is a relatively new approach that has shown improved transfection efficiency and gene silencing compared to other non-viral vectors	Lower risk of immune response and toxicity compared to viral vectors	Lower transfection efficiency and gene expression levels compared to viral vectors	[49, 50]
**CRISPR-Cas9**	PD-1	Gold nanoparticles	High	High	Enhances T cell activity	Gold nanoparticles have unique optical and electronic properties that make them promising candidates for gene therapy	The use of gold nanoparticles for CRISPR-Cas9 delivery is a cutting-edge approach that has shown promising results in preclinical studies	High transfection efficiency and gene expression levels	Limited data on long-term safety and potential toxicity	[117]
**Electroporation**	IL-12	Electric pulses	Moderate	High	Induces immune response	Electroporation is a non-viral method for delivering nucleic acids into cells using brief electric pulses	The use of electroporation for IL-12 gene therapy is a novel approach that has shown promising results in preclinical studies	Non-toxic and non-immunogenic	Requires specialized equipment and expertise	[251]
**CRISPR-Cas13a**	KRAS	Liposomes	High	Moderate	Downregulates target gene expression	CRISPR-Cas13a is a recently discovered RNA-guided ribonuclease system that can be used for RNA editing	The use of CRISPR-Cas13a for KRAS gene therapy is a cutting-edge approach that has shown promising results in preclinical studies	Specific and efficient targeting of RNA	Limited data on long-term safety and potential off-target effects	[252]
**AAV vectors**	BDNF	Adeno-associated virus	High	High	Enhances neuronal growth and survival	Adeno-associated virus (AAV) vectors are a type of viral vector that can be used for gene therapy due to their safety and ability to integrate into the host genome	The use of AAV vectors for brain-derived neurotrophic factor (BDNF) gene therapy is a promising approach for treating neurodegenerative diseases	Long-term expression and stability	Limited packaging capacity and potential immune response	[253]
**Hybrid viral vectors**	HER2	Hybrid viral vectors	High	High	Induces apoptosis	Hybrid viral vectors combine the advantages of different viral vectors to achieve improved transduction efficiency and specificity	The use of hybrid viral vectors for HER2 gene therapy is a promising approach for treating HER2-positive breast cancer	Improved transduction efficiency and specificity compared to single viral vectors	Potential immune response and toxicity	[254]
**CRISPR-Cas9**	LDLR	Gold nanorods	Moderate	Moderate	Upregulates target gene expression	Gold nanorods are a type of gold nanoparticle that can be used for gene therapy due to their plasmonic properties	The use of gold nanorods for CRISPR-Cas9-mediated LDLR gene therapy is a novel approach that has shown promising results in preclinical studies	Non-toxic and biocompatible	Limited data on long-term safety and efficacy	[117]
**Non-viral vectors**	BRCA1	Polymeric nanoparticles	High	High	Induces DNA repair	Polymeric nanoparticles are a type of non-viral vector that can be used for gene therapy due to their versatility and biocompatibility	The use of polymeric nanoparticles for BRCA1 gene therapy is a promising approach for treating breast cancer	High transfection efficiency and biocompatibility	Limited packaging capacity and potential toxicity	[49, 50]
**In vivo electroporation**	IL-10	In vivo electroporation	High	High	Suppresses inflammation	In vivo electroporation is a non-viral method for delivering nucleic acids into cells in vivo using electric pulses	The use of in vivo electroporation for IL-10 gene therapy is a promising approach for treating inflammatory diseases	Non-toxic and non-immunogenic	Limited data on long-term safety and potential toxicity	[255]
**CRISPR-Cas12a**	EGFR	Lipid nanoparticles	High	Moderate	Downregulates target gene expression	CRISPR-Cas12a is a recently discovered RNA-guided endonuclease system that can be used for gene editing	The use of CRISPR-Cas12a for EGFR gene therapy is a novel approach that has shown promising results in preclinical studies	Specific and efficient targeting of DNA	Limited data on long-term safety and potential off-target effects	[256]
**mRNA-based vaccines**	SARS-CoV-2 spike protein	Lipid nanoparticles	High	High	Induces immune response	mRNA-based vaccines are a novel approach to gene therapy that use messenger RNA (mRNA) to encode a protein of interest and induce an immune response	The use of mRNA-based vaccines for the SARS-CoV-2 spike protein is a cutting-edge approach for preventing COVID-19	Rapid and scalable production	Potential immune response and toxicity	[257]
**CRISPR-Cas9**	HPRT	Zinc-finger nucleases	Moderate	Moderate	Gene correction	Zinc-finger nucleases (ZFNs) are a type of engineered DNA-cutting enzyme that can be used for gene editing	The use of ZFNs for HPRT gene therapy is a promising approach for treating genetic disorders	Specific and efficient targeting of DNA	Limited data on long-term safety and potential off-target effects	[117, 258]
**Viral vectors**	CFTR	Lentivirus	High	High	Induces gene expression	Lentivirus is a type of retrovirus that can be used as a vector for gene therapy	The use of lentivirus for CFTR gene therapy is a promising approach for treating cystic fibrosis	High transduction efficiency and stable gene expression	Potential immune response and toxicity	[259]
**Non-viral vectors**	VEGF	Dendrimers	Moderate	Moderate	Enhances angiogenesis	Dendrimers are a type of branched polymer that can be used as a non-viral vector for gene therapy	The use of dendrimers for VEGF gene therapy is a novel approach that has shown promising results in preclinical studies	Highly customizable and biocompatible	Limited transfection efficiency and potential toxicity	[260]
**CRISPR-Cas9**	DMD	AAV vectors	High	High	Gene correction	Adeno-associated virus (AAV) vectors are a type of viral vector that can be used for gene therapy	The use of AAV vectors for DMD gene therapy is a promising approach for treating Duchenne muscular dystrophy	Long-term gene expression and safety	Limited packaging capacity and potential immune response	[117]
**mRNA-based therapies**	OCT4	mRNA electroporation	High	High	Induces cell reprogramming	mRNA-based therapies are a novel approach to gene therapy that use messenger RNA (mRNA) to encode a protein of interest	The use of mRNA electroporation for OCT4 gene therapy is a cutting-edge approach for inducing cell reprogramming	Rapid and customizable production	Potential immune response and toxicity	[261]
**Non-viral vectors**	MDR1	Nanoparticles	Moderate	Moderate	Downregulates target gene expression	Nanoparticles are a type of non-viral vector that can be used for gene therapy due to their size and biocompatibility	The use of nanoparticles for MDR1 gene therapy is a promising approach for overcoming multidrug resistance in cancer	Non-toxic and biocompatible	Limited transfection efficiency and gene expression levels	[49, 50]
**CRISPR-Cas9**	HBB	Lipid nanoparticles	High	High	Gene correction	Lipid nanoparticles are a type of non-viral vector that can be used for gene therapy due to their biocompatibility and ease of production	The use of lipid nanoparticles for HBB gene therapy is a promising approach for treating sickle cell disease	Non-immunogenic and scalable production	Limited transfection efficiency and gene expression levels	[117]
**Gene silencing**	BCL-2	Aptamers	High	Moderate	Downregulates target gene expression	Aptamers are a type of synthetic nucleic acid that can be used as a gene silencing agent	The use of aptamers for BCL-2 gene therapy is a novel approach that has shown promising results in preclinical studies	Specific and efficient targeting of RNA	Limited in vivo stability and potential immunogenicity	[262]
**Non-viral vectors**	FGF2	Cationic liposomes	Moderate	Moderate	Enhances angiogenesis	Cationic liposomes are a type of non-viral vector that can be used for gene therapy due to their ability to interact with cell membranes	The use of cationic liposomes for FGF2 gene therapy is a promising approach for promoting tissue regeneration	Low toxicity and customizable	Limited transfection efficiency and stability	[49, 50, 263]
**CRISPR-Cas9**	CFTR	CRISPR-Cas9 ribonucleoprotein	High	High	Gene correction	CRISPR-Cas9 ribonucleoprotein is a recently developed gene editing technology that uses RNA-guided endonucleases	The use of CRISPR-Cas9 ribonucleoprotein for CFTR gene therapy is a promising approach for treating cystic fibrosis	High specificity and efficiency	Potential off-target effects and immune response	[117, 264]
**RNA interference**	KRAS	Gold nanoparticles	Moderate	Moderate	Downregulates target gene expression	Gold nanoparticles are a type of nanoparticle that can be used for gene therapy due to their unique optical and electronic properties	The use of gold nanoparticles for KRAS gene therapy is a novel approach that has shown promising results in preclinical studies	High biocompatibility and stability	Limited transfection efficiency and potential toxicity	[194, 195]
**Viral vectors**	APOE	Adenovirus	High	High	Upregulates target gene expression	Adenovirus is a type of viral vector that can be used for gene therapy due to its high transduction efficiency	The use of adenovirus for APOE gene therapy is a promising approach for treating Alzheimer's disease	High transduction efficiency and long-term gene expression	Potential immune response and toxicity	[265]
**RNA interference**	TP53	Nanoparticles	High	High	Downregulates target gene expression	Nanoparticles are a type of non-viral vector that can be used for gene therapy due to their size and biocompatibility	The use of nanoparticles for TP53 gene therapy is a promising approach for treating various cancers	Non-toxic and biocompatible	Limited transfection efficiency and gene expression levels	[193]
**Gene editing**	F9	CRISPR-Cas9	High	High	Gene correction	CRISPR-Cas9 is a gene editing technology that uses RNA-guided endonucleases to modify DNA	The use of CRISPR-Cas9 for F9 gene therapy is a promising approach for treating hemophilia B	High specificity and efficiency	Potential off-target effects and immune response	[266]
**mRNA-based therapies**	FLT3L	mRNA electroporation	High	High	Induces immune response	mRNA-based therapies are a novel approach to gene therapy that use messenger RNA (mRNA) to encode a protein of interest	The use of mRNA electroporation for FLT3L gene therapy is a cutting-edge approach for enhancing immune response	Rapid and customizable production	Potential immune response and toxicity	[194, 195]

### Methods developed with the express purpose of combating TME

**Fig. 19:**
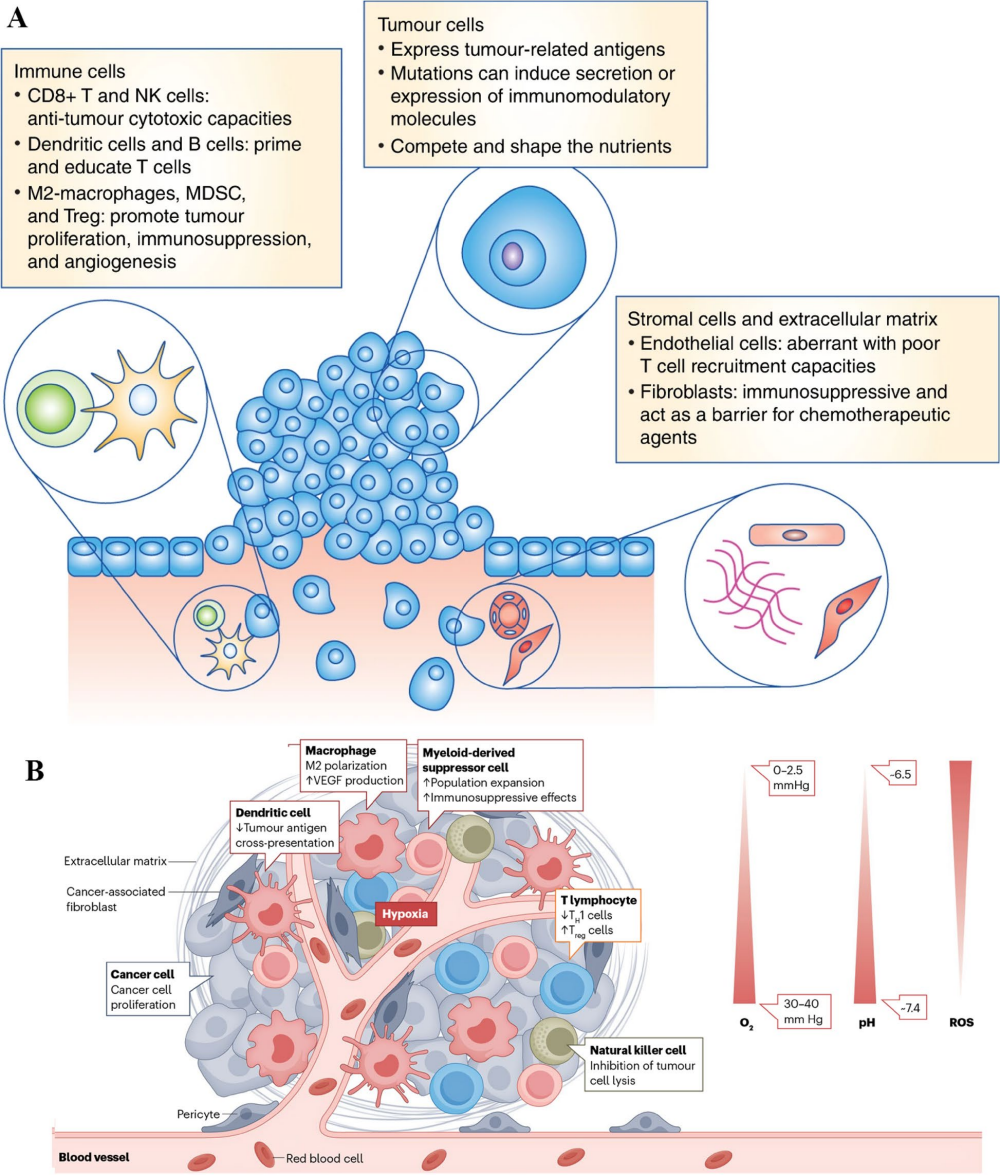
**A** The key elements within the cancerous tumor environment, focusing on how immune cells are influenced. It highlights the role of MDSCs (myeloid-derived suppressor cells) and Tregs (regulatory CD4 + T cells) in shaping the immune cell composition within this environment. Reprint from [268] with a permission from Springer Nature. **B** The Cancer Microenvironment. The physical and chemical characteristics of the cancer microenvironment, such as oxygen levels, pH, and reactive oxygen species (ROS), have an impact on both cancer and immune cells. M2 refers to M2-type macrophages, while VEGF denotes vascular endothelial growth factor. Reprint from [244] with a permission from Springer Nature

Targeting tumor microenvironments (TME) has emerged as a promising strategy in cancer treatment, with several mechanisms being explored to combat the complex network of cells and factors that support tumor growth and progression (Fig. 19-A). One prominent approach is immunotherapy, which aims to activate the patient's immune system to recognize and attack cancer cells within the TME [190]. Checkpoint inhibitors, such as PD-1 and CTLA-4 inhibitors, have shown remarkable success in this regard [241]. Additionally, researchers are investigating strategies to normalize the abnormal blood vessels found in the TME, enhancing drug delivery to the tumor. Angiogenesis inhibitors, like anti-VEGF drugs, are employed to hinder the formation of new blood vessels within the tumor. Furthermore, the development of targeted therapies that disrupt specific signaling pathways crucial for TME maintenance, such as the PI3K/AKT/mTOR pathway, holds promise in altering the TME to make it less hospitable to cancer cells [190]. Collectively, these mechanisms offer a multifaceted approach to target the TME and improve the efficacy of cancer treatment, potentially leading to more effective and personalized therapeutic strategies [267]. Most cancers have very active angiogenesis because their unchecked cell division requires a great deal of energy. This is so because, as was just said, almost all tumors are cancerous. Positive results from studies focusing on this quality were found. Sengupta developed a nanoparticle delivery approach to specifically target aberrant tumor angiogenesis by encapsulating the drug combretastatin into the PLGA core with the chemotherapy drug DOX [90, 102]. Due to combretastatin causing rapid closure of malignant arteries, the DOX was readily absorbed by the tumor. With this improvement, the therapeutic index was raised while the harmful effects were minimized. Extracellular matrix, generally known as ECM, has been the focus of investigation in the area of cancer treatment, in addition to aberrant vasculature [267]. In cancer proliferation, migration, invasion, and angiogenesis, the extracellular matrix (ECM) works as a guiding scaffold [241]. These carcinogenic properties are contributed mostly by collagen, HA, and a variety of enzymes. Hydroxyapatite (HA) contributes to high interstitial fluid pressure (IFP), which impedes medicine diffusion and penetration, while collagen (the principal structural protein of the ECM) is responsible for establishing migratory pathways for tumor cells. Matrix metalloproteinases (MMPs) and other enzymes control TME through modulating the activity of molecules that are not part of the extracellular matrix [105]. Growth factors, receptors, and cytokines are examples of non-ECM molecules. Electron-current-matter (ECM) interaction is one factor to consider while developing nanocarriers. Patients with metastatic pancreatic cancer benefited from a combination of standard chemical medicines and a PEGylated type of recombinant human hyaluronidase (PEGPH20) that targets ECM hyaluronic acid [15]. Cancer patients whose tumor cells produced a lot of hyaluronidases were hit the worst by these changes. There have been attempts made, such as coating nanocarriers with hyaluronidase, to increase the ability of chemical treatments conveyed by nanocarriers to enter solid tumors (HAase). This easy-to-implement strategy still achieves success and is very powerful against tumors [190]. Figure 19-B illustrates the complex interactions within the TME, where various physicochemical factors play critical roles in shaping cancer progression and immune responses. Oxygen levels, pH, and reactive oxygen species (ROS) in the TME directly influence the behavior and function of cancer cells and immune cells, including M2-type macrophages. In response to these conditions, cancer cells can release vascular endothelial growth factor (VEGF), which promotes angiogenesis, further contributing to the heterogeneity of the TME. The presence of M2-type macrophages, known for their tumor-promoting characteristics, also impacts the overall TME dynamics. Collectively, these components and their interactions within the TME determine the fate of tumor growth and metastasis, as well as the efficacy of immune responses and potential therapies.

### The use of nanomaterials in combination with immunotherapy treats cancer patients

**Fig. 20:**
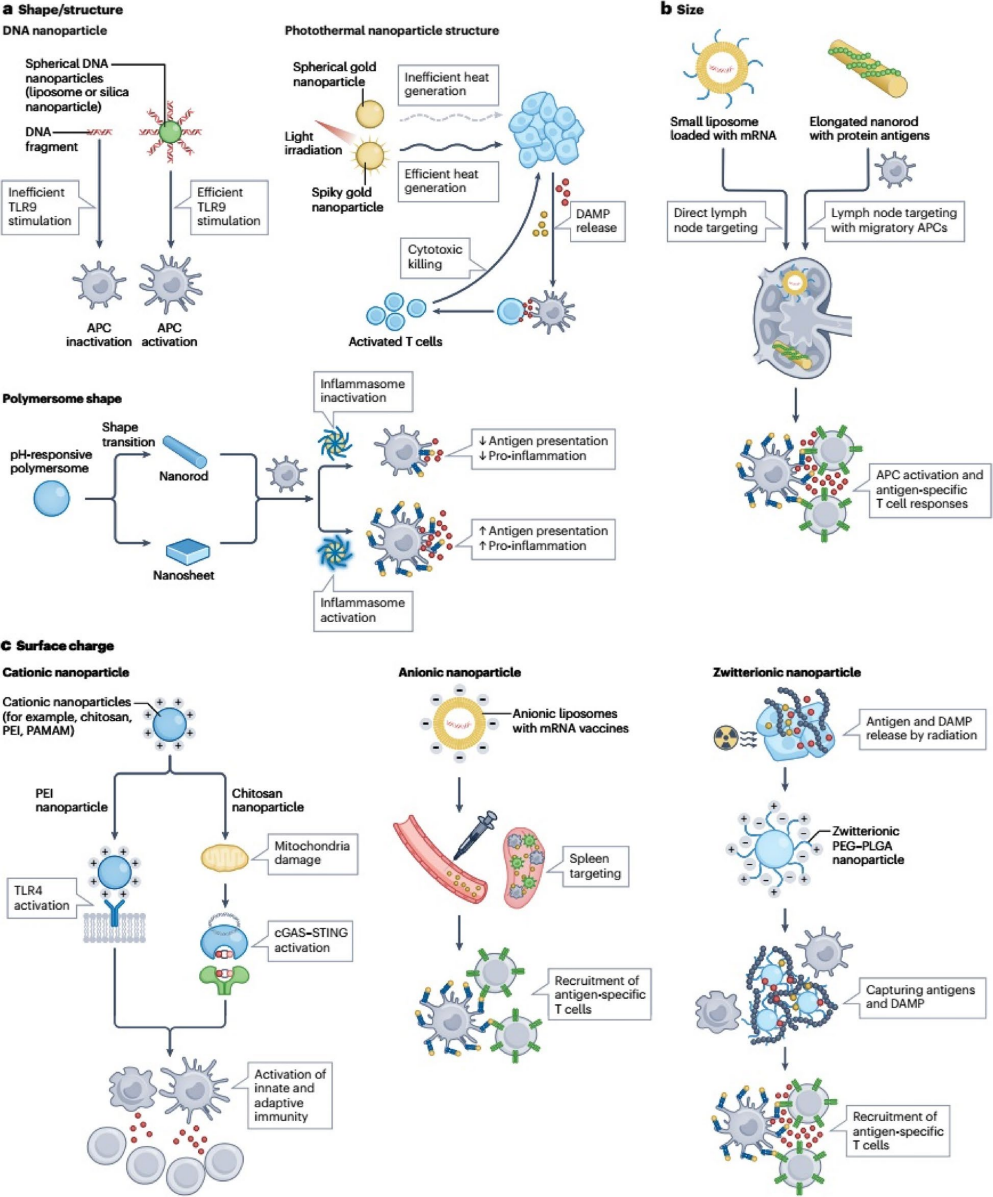
The regulation of immune responses in cancer immunotherapy by nanomaterials' physical properties. **A**, The shape of nanomaterials can directly or indirectly influence immune responses in innate immune cells. Spherical DNA nanoparticles more effectively stimulate the TLR9 pathway to enhance innate immunity compared to linear DNA fragments. Pointed gold nanoparticles have a higher photothermal efficiency than spherical ones, resulting in greater DAMP release and stronger antitumor immunity. pH-responsive shape transitions from spheres to nanosheets promote inflammasome activation by destabilizing lysosomes, yielding better antitumor immunity than nanorods. The size of organic or inorganic nanomaterials impacts lymph node targeting and nanoparticle retention kinetics, affecting both innate and adaptive immunity for antigen-specific immunogenicity. **B**, Nanoparticle size influences immunological responses in both innate and adaptive immunity. Adjusting nanoparticle size affects targeting locations; smaller nanoparticles target lymph nodes, while larger ones target antigen-presenting cells (APCs). Large nanoparticles with CD3/CD28 antibodies bind more effectively to T cell receptors than small ones, enhancing T cell immunity. Mesoporous silica nanoparticles with larger pores enable rapid release of immunostimulatory molecules, sensitizing APCs to trigger antitumor responses. **C**, The surface charge of nanomaterials can directly or indirectly stimulate immune responses. Cationic nanoparticles boost innate immune signaling in APCs, leading to antitumor responses. Anionic nanoparticles, such as mRNA vaccines, when administered systemically, preferentially target the spleen, inducing tumor-specific immunity. Zwitterionic nanoparticles can capture antigens and release DAMPs from dying tumor cells, reprogramming APCs to activate antigen-specific T cell immune responses. Reprint from [130] with a permission from Springer Nature

In the realm of cancer research, several theories are relevant to understanding the interplay between tumors and the immune system. These theories provide crucial insights into the mechanisms by which cancer cells evade immune surveillance and how immunotherapy can be applied to counteract these evasive strategies [215]. One prominent theory is the Tumor Immune Evasion Theory, which posits that tumors can develop mechanisms to evade the immune system, enabling their unchecked growth. Immune checkpoint inhibitors, such as PD-1/PD-L1 or CTLA-4 inhibitors, have emerged as key tools in immunotherapy. These inhibitors block signals that would otherwise prevent immune cells from attacking cancer cells, thereby enhancing the immune response against the tumor [214]. Another pivotal theory is the Cancer Immunoediting Theory, which describes the dynamic interaction between the immune system and developing tumors. This theory identifies three phases: elimination, equilibrium, and escape [269]. Immunotherapy strategies aim to enhance the elimination phase and prevent the escape of cancer cells from immune surveillance [241]. Personalized treatments based on a patient's unique tumor antigens are also a focus of research within this theory [269]. The Tumor Microenvironment Theory underscores the significance of the tumor's surroundings [15]. Tumor microenvironments consist of various components, including immune cells, stromal cells, and blood vessels. Some elements within this microenvironment can be immunosuppressive and hinder immune responses against tumors, facilitating tumor growth [215]. Immunotherapy can modulate the tumor microenvironment to make it more conducive to immune attacks [269]. Combination therapies, like pairing checkpoint inhibitors with drugs targeting angiogenesis or stromal cells, aim to disrupt the tumor's protective shield [15]. Lastly, the Cancer Stem Cell Theory proposes that tumors contain a subpopulation of cancer stem cells with self-renewal and tumor-initiating properties. These cells are often resistant to conventional treatments and immune responses [214]. Immunotherapy approaches targeting cancer stem cells involve identifying unique markers or antigens associated with these cells. Such targeted strategies can disrupt tumor growth and prevent recurrence by eliminating the source of tumor initiation [269]. The immune system plays a crucial role in cancer's initiation and progression. Figure 20 highlights the significant role of nanomaterials' physical properties in regulating immune responses during cancer immunotherapy. Nanomaterial shape (Fig. 20-A) can directly or indirectly influence immune responses in innate immune cells. For example, spherical DNA nanoparticles stimulate the TLR9 pathway more effectively than linear DNA fragments, while pointed gold nanoparticles exhibit higher photothermal efficiency, leading to stronger antitumor immunity. The size of nanomaterials (Fig. 20-B) impacts lymph node targeting, retention kinetics, and overall immunogenicity. By adjusting nanoparticle size, one can selectively target lymph nodes or antigen-presenting cells (APCs). Larger nanoparticles, such as those with CD3/CD28 antibodies, bind more effectively to T cell receptors, thus enhancing T cell immunity. Moreover, surface charge (Fig. 20-C) can directly or indirectly stimulate immune responses, with cationic nanoparticles boosting innate immune signaling in APCs, anionic nanoparticles inducing tumor-specific immunity, and zwitterionic nanoparticles capturing antigens and releasing DAMPs from dying tumor cells to activate antigen-specific T cell immune responses. Cancer vaccine therapy, immune checkpoint blockade therapy, chimeric antigen receptor (CAR)-T cell therapy, and immune system modulator therapy are only a few of the immunotherapeutic approaches now in use [215]. These cancer immunotherapies employ either naturally occurring chemicals or synthesized substances to stimulate or restore immune system function in order to provide an anti-tumor effect. Programmed cell death protein 1 (PD-1) and programmed cell death ligand 1 (PD-L1) are two crucial immunological checkpoints (PD-L1). There has been investigation into the feasibility of loading immune checkpoint inhibitors (ICIs) that target PD-1/PD-L1 onto nanocarriers for the purpose of cancer therapy. The typical immune checkpoint inhibitors (ICIs) of PD-1/PD-L1 showed variable advantages, and research conducted by BU and other institutions suggested that over-expression of PD-1 allowed cancer cells to perform antitumor immunity evasion [215]. To provide a strong connection between PD-L1 and ICIs, multivalent poly (amidoamine) dendrimers were used. This enhanced medication accumulation at the tumor location and further enhanced the PD-L1 inhibiting action. The cytotoxic T-lymphocyte-associated protein 4, or CTLA-4, may suppress immune responses and act as an immunological checkpoint. Some common examples of such molecules are antibodies, proteins, and small-molecule inhibitors. One important role that nanoparticles play in the delivery of drugs is as carriers for the drugs themselves. Using these technologies, new nanoplatforms could be made, and it is hoped that they will be more effective and bioavailable than current therapies [269]. This approach includes the use of immune checkpoint inhibitors, oncolytic viruses, adoptive cell therapy, immune stimulators, and cytokine therapy in combination with various nanoparticle drug delivery systems. These combinations have been shown to have synergistic effects, improving the overall efficacy of the treatments [241]. The administration sequence and dose ratio of these drug combinations vary, with some being administered simultaneously and others sequentially. Despite the promising therapeutic outcomes, these combinations may lead to adverse effects such as immune-related adverse events, flu-like symptoms, injection site reactions, and neurotoxicity. Table 12 highlights the potential of combining nanomaterials with immunotherapy for the treatment of cancer patients [215]. Nanoparticles have gained significant attention in the field of cancer immunotherapy due to their versatile applications. These nanomaterials primarily target cancer cells, allowing for enhanced drug delivery while minimizing side effects. By encapsulating immunotherapeutic agents within liposomal nanoparticles, researchers have been able to achieve controlled and precise drug delivery to tumor sites [269]. However, challenges remain, such as the potential for these nanoparticles to evade the immune system and concerns regarding their toxicity. Some examples of these nanoparticles in action include liposomal nanoparticles loaded with immunotherapeutic agents for cancer treatment [15]. Quantum dots, another class of nanomaterials, are being explored for their role in targeted immunotherapy. These tiny semiconductor particles have shown promise in the precise imaging and targeting of tumor-associated antigens, allowing for real-time monitoring of immunotherapy progress. However, quantum dots also face challenges, including potential toxicity and concerns about immunogenicity. Researchers have developed quantum dot-based systems for targeted drug delivery with the goal of improving cancer treatment [270]. Nanotubes, such as carbon nanotubes, have emerged as potential vehicles for delivering immune checkpoint inhibitors in cancer immunotherapy. They offer advantages like controlled drug release and sustained immunomodulation. However, issues related to their clearance from the body and concerns about biocompatibility have to be addressed. Some studies have explored the use of carbon nanotubes for the delivery of immune checkpoint inhibitors, aiming to enhance their therapeutic effectiveness [109]. Nanoparticles have also found applications in the development of vaccines for immunotherapy. By improving antigen presentation and enhancing the immune response, nanoparticle-based vaccines show promise. However, challenges include limited vaccine stability and potential toxicity. Researchers have investigated the use of gold nanoparticles to develop cancer vaccines, with the aim of boosting their efficacy [15].

**Table 12:** The use of nanomaterials in combination with immunotherapy treats cancer patients

**Therapeutic Approach**	**Drug Combination**	**Administration Sequence**	**Synergy**	**Adverse Effects**	**References**
**Immune Checkpoint Inhibitor + Nanoparticle Drug Delivery System**	Anti-PD-1 antibody + Paclitaxel-loaded polymeric nanoparticles	Simultaneous	Synergistic	Immune-related adverse events (irAEs) such as rash, fatigue, diarrhea	[215]
**Oncolytic Virus + Nanoparticle Drug Delivery System**	T-VEC + Docetaxel-loaded liposomal nanoparticles	Sequential	Synergistic	Influenza-like symptoms, mild rash	[215]
**Adoptive Cell Therapy + Nanoparticle Drug Delivery System**	CAR-T cells + siRNA-loaded nanoparticles	Simultaneous	Synergistic	Cytokine release syndrome, neurotoxicity	[214]
**Immune Stimulator + Nanoparticle Drug Delivery System**	CpG-ODN + Doxorubicin-loaded liposomal nanoparticles	Sequential	Synergistic	Flu-like symptoms, injection site reactions	[269]
**Nanoparticle Drug Delivery System + Cytokine Therapy**	IL-2-loaded nanoparticles + Doxorubicin-loaded liposomes	Simultaneous	Synergistic	Fever, chills, hypotension	[271]
**Immune Checkpoint Inhibitor + Nanoparticle Drug Delivery System**	Anti-CTLA-4 antibody + Sirolimus-loaded polymeric nanoparticles	Sequential	Synergistic	Diarrhea, rash, colitis	[241]
**Oncolytic Virus + Nanoparticle Drug Delivery System**	Adenovirus + Docetaxel-loaded lipid nanoparticles	Simultaneous	Synergistic	Fatigue, fever, nausea	[215]
**Adoptive Cell Therapy + Nanoparticle Drug Delivery System**	TILs + Paclitaxel-loaded dendrimers	Simultaneous	Synergistic	Cytokine release syndrome, neurotoxicity	[215]
**Immune Stimulator + Nanoparticle Drug Delivery System**	CpG-ODN + Curcumin-loaded polymeric nanoparticles	Sequential	Synergistic	Injection site reactions, mild gastrointestinal symptoms	[269]
**Nanoparticle Drug Delivery System + TLR Agonist**	Imiquimod-loaded nanoparticles + Gemcitabine	Simultaneous	Synergistic	Injection site reactions, flu-like symptoms	[43]
**Immune Checkpoint Inhibitor + Nanoparticle Drug Delivery System**	Anti-PD-1 antibody + Docetaxel-loaded nanocrystals	Sequential	Synergistic	Fatigue, neutropenia, anemia	[15]
**Oncolytic Virus + Nanoparticle Drug Delivery System**	Measles virus + Paclitaxel-loaded liposomes	Simultaneous	Synergistic	Injection site reactions, fever, fatigue	[215]
**Adoptive Cell Therapy + Nanoparticle Drug Delivery System**	CAR-T cells + Nanogels loaded with anti-PD-1 antibody	Simultaneous	Synergistic	Cytokine release syndrome, hypotension	[214]
**Immune Stimulator + Nanoparticle Drug Delivery System**	Poly(I:C) + Doxorubicin-loaded liposomes	Sequential	Synergistic	Flu-like symptoms, injection site reactions	[269]
**Nanoparticle Drug Delivery System + Cancer Vaccine**	Polymeric nanoparticles loaded with tumor antigens + Adjuvant	Simultaneous	Synergistic	Injection site reactions, fever	[101]
**Immune Checkpoint Inhibitor + Nanoparticle Drug Delivery System**	Anti-PD-L1 antibody + Paclitaxel-loaded nanofibers	Sequential	Synergistic	Nausea, fatigue, neuropathy	[215]
**Nanoparticle Drug Delivery System + Chemotherapy**	Oxaliplatin-loaded nanoparticles + 5-FU	Simultaneous	Synergistic	Peripheral neuropathy, diarrhea	[49, 50]
**Immune Checkpoint Inhibitor + Nanoparticle Drug Delivery System**	Anti-CTLA-4 antibody + Paclitaxel-loaded micelles	Sequential	Synergistic	Diarrhea, fatigue, neutropenia	[272]
**Oncolytic Virus + Nanoparticle Drug Delivery System**	Newcastle disease virus + Irinotecan-loaded liposomes	Simultaneous	Synergistic	Influenza-like symptoms, mild rash	[25, 26]
**Adoptive Cell Therapy + Nanoparticle Drug Delivery System**	CAR-T cells + DOX-loaded gold nanoparticles	Simultaneous	Synergistic	Cytokine release syndrome, neurotoxicity	[214]
**Immune Stimulator + Nanoparticle Drug Delivery System**	MPLA + Gemcitabine-loaded liposomes	Sequential	Synergistic	Injection site reactions, flu-like symptoms	[269]
**Nanoparticle Drug Delivery System + TLR Agonist**	R848-loaded nanoparticles + Docetaxel	Simultaneous	Synergistic	Injection site reactions, myelosuppression	[43]
**Immune Checkpoint Inhibitor + Nanoparticle Drug Delivery System**	Anti-PD-1 antibody + Doxorubicin-loaded carbon nanotubes	Sequential	Synergistic	Fatigue, neutropenia, anemia	[109]
**Oncolytic Virus + Nanoparticle Drug Delivery System**	Reovirus + Cisplatin-loaded liposomes	Simultaneous	Synergistic	Injection site reactions, flu-like symptoms	[215]
**Adoptive Cell Therapy + Nanoparticle Drug Delivery System**	TCR-T cells + Paclitaxel-loaded solid lipid nanoparticles	Simultaneous	Synergistic	Cytokine release syndrome, neurotoxicity	[215]
**Immune Stimulator + Nanoparticle Drug Delivery System**	R848 + Gemcitabine-loaded dendrimers	Sequential	Synergistic	Injection site reactions, flu-like symptoms	[43]
**Nanoparticle Drug Delivery System + Cancer Vaccine**	PLGA nanoparticles loaded with tumor antigen + CpG-ODN	Simultaneous	Synergistic	Injection site reactions, fever	[273]
**Immune Checkpoint Inhibitor + Nanoparticle Drug Delivery System**	Anti-PD-L1 antibody + Paclitaxel-loaded nanocapsules	Sequential	Synergistic	Nausea, fatigue, neuropathy	[215]

### Metabolic effects of nanomaterials on drugs

**Fig. 21:**
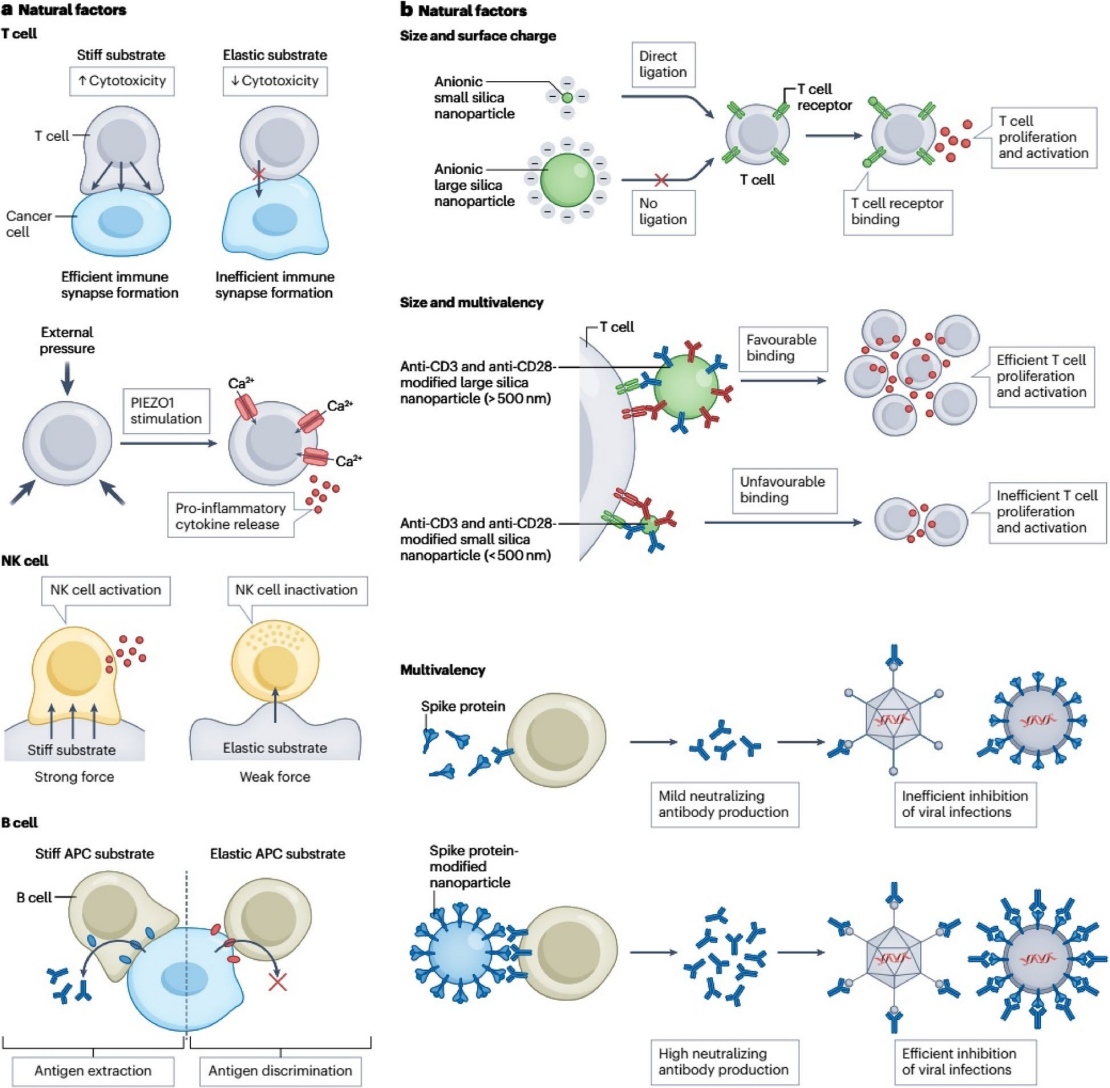
Impact of nanomaterial physical properties on immune cell function. **A**, T cell immunity is affected by substrate stiffness and external forces. Rigid substrates facilitate the formation of immunological synapses with T cells, enhancing their cytotoxic capabilities. Mechanical stress activates T cells by stimulating PIEZO1 mechanosensory ion channels. Substrate rigidity is also crucial for regulating natural killer (NK) cell immunity. B cells selectively extract antigens from APCs based on the rigidity of the APC membranes. **B**, Several physical factors in nanomaterial design influence T cell activation. The dimensions and surface charges of nanomaterials modify direct binding to T cell receptors. The size and multivalency of nanoparticles mimicking APCs affect T cell activation and growth. Multivalent spiky protein nanoparticles interact more effectively with B cell receptors, promoting antibody production compared to uncoated spike proteins. Reprint from [130] with a permission from Springer Nature

**Fig. 22:**
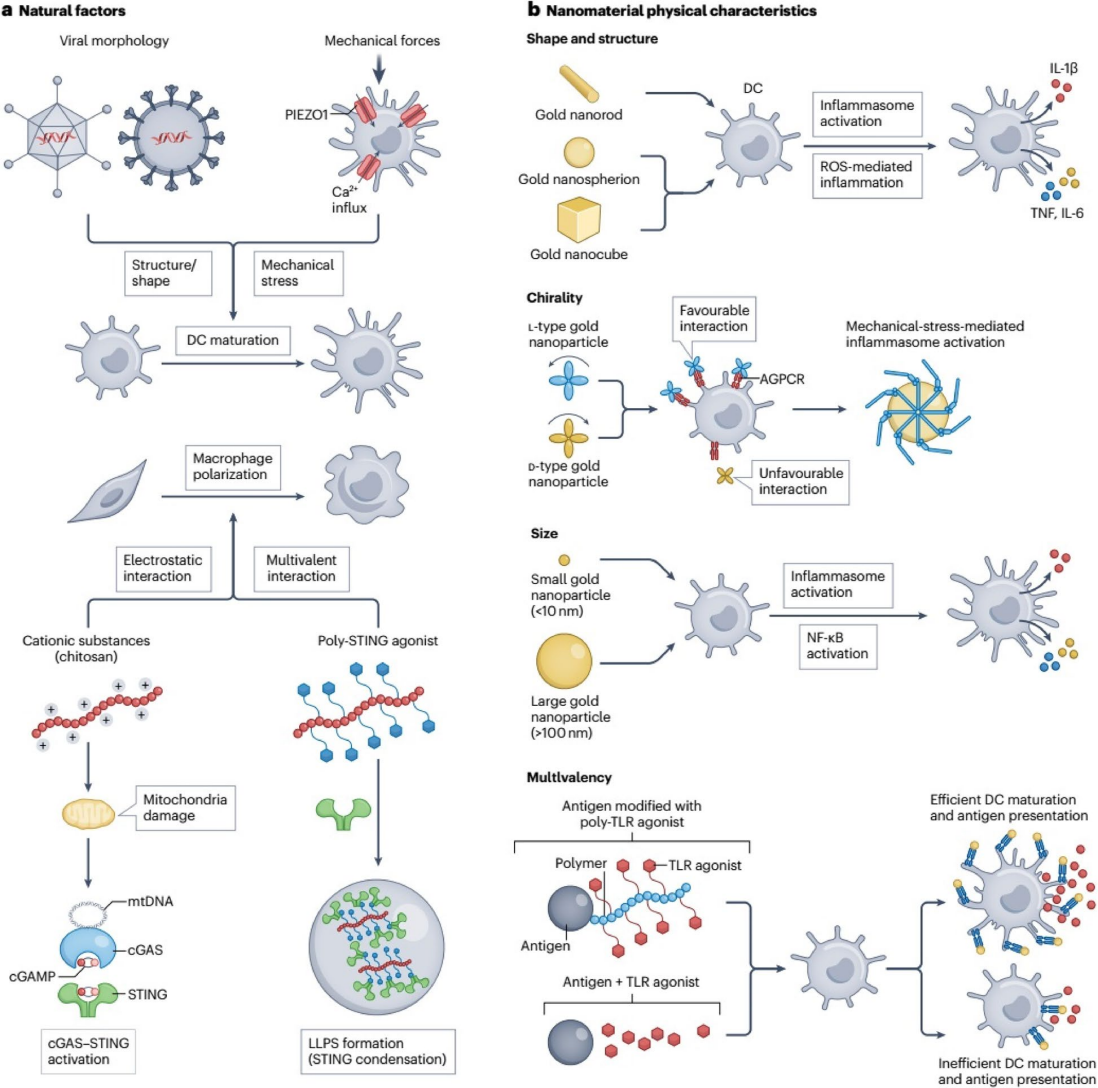
Factors affecting immune functions in dendritic cells and macrophages. **A**, The immune responses of dendritic cells (DCs) and macrophages are influenced by the physical properties of their surrounding environment, including shape, mechanical forces, surface charge, and multivalency. DCs and macrophages can detect the shape of foreign substances (e.g., viruses) and modify immune signaling accordingly. Mechanical stress activates PIEZO1 ion channels in antigen-presenting cells, leading to calcium influx and cell activation. Natural polysaccharides with cationic charges (e.g., chitosan) can damage mitochondria, causing the release of mitochondrial DNA (mtDNA) and the upregulation of type I interferon responses via the cGAS-STING pathway. Poly-STING agonists activate STING signaling through multivalent interactions that cause STING condensation. **B**, The physical properties of nanomaterials, such as shape, structure, chirality, size, and multivalency, can affect innate immune signaling in DCs and macrophages. Different shapes and structures of gold nanoparticles can alter pro-inflammatory signaling pathways (nanorods activate NLRP3 inflammasomes; nanospheres and nanocubes induce ROS-mediated inflammation). The chirality of inorganic nanoparticles can also impact immunogenicity by interacting with specific chiral receptors like adhesion G protein-coupled receptors (AGPCRs). Small gold nanoparticles (< 10 nm) stimulate the inflammasome axis, while large gold nanoparticles (> 100 nm) activate NF-κB pathways. Multivalent TLR agonists combined with antigens enhance DC maturation and antigen cross-presentation more efficiently. cGAMP, cyclic GMP–AMP; LLPS, liquid–liquid phase separation; TNF, tumor necrosis factor. Reprint from [130] with a permission from Springer Nature

Figure 21 illustrates the significant impact of nanomaterial physical properties on immune cell function. In particular, T cell immunity is influenced by substrate stiffness and external forces, with rigid substrates enhancing cytotoxic capabilities through the facilitation of immunological synapse formation. Additionally, mechanical stress serves as a stimulant for T cells by activating PIEZO1 mechanosensory ion channels. Substrate rigidity plays a crucial role in regulating natural killer (NK) cell immunity as well. Furthermore, B cells demonstrate selectivity in antigen extraction from antigen-presenting cells (APCs) based on the rigidity of the APC membranes. The physical factors in nanomaterial design, such as dimensions and surface charges, also have implications for T cell activation by modifying direct binding to T cell receptors. The size and multivalency of nanoparticles that mimic APCs are important determinants of T cell activation and growth. Notably, multivalent spiky protein nanoparticles exhibit enhanced interaction with B cell receptors, leading to a more efficient promotion of antibody production compared to uncoated spike proteins. Drug metabolism is a convoluted process. The MPS, which is also known as the reticuloendothelial system or the macrophage system, is a network of immune cells that includes both blood-borne monocytes and tissue-based macrophages. Components of the MPS, such as immune cells in the liver, spleen, or lungs, may react to exogenous molecules, in this case, chemical drugs [92]. The drugs' half-life will be significantly shortened due to the rapid elimination by activated macrophages or leukocytes. Surface modifications, such as PEG or a specific peptide, on nanocarriers have been demonstrated to inhibit MPS clearance, resulting in an increased half-life of the medication. Important to the function of the kidneys is their ability to filter blood and other chemicals. Numerous characteristics, including particle size, shape, and surface charge, correlate with renal clearance rate. Renal clearance is a crucial component in the dispersion of traditional pharmacologic medicines [9]. Optimal renal clearance is crucial for decreasing nanocarrier toxicity. Table 13 highlights the toxicity profiles of various nanocarrier types, revealing important information about their potential hazards and how to mitigate them. For example, liposomes can induce dose-dependent hepatotoxicity through the generation of reactive oxygen species (ROS), resulting in apoptosis in an acute time frame. To counteract this, the use of antioxidants or reduction of drug dose can be employed. Polymeric nanoparticles, on the other hand, exhibit nonlinear nephrotoxicity in a sub-acute duration, primarily due to accumulation in renal tubules and glomeruli. PEGylation and adjustment of molecular weight are viable mitigation strategies for this issue. Carbon nanotubes cause dose-dependent pulmonary toxicity, which manifests as inflammation, oxidative stress, fibrosis, and granuloma formation over a chronic time course. Surface modification and reducing the length and aspect ratio of the nanotubes can minimize these adverse effects. Gold nanoparticles display dose-dependent cytotoxicity in a sub-chronic period, primarily through the uptake and accumulation in mitochondria, inducing oxidative stress and apoptosis. Surface coating and the use of size-limited particles can help mitigate these risks. Iron oxide nanoparticles lead to dose-dependent hemotoxicity in an acute time frame, stemming from ROS-induced apoptosis and complement activation. Surface coating and chelation of iron ions can address these concerns. Lastly, dendrimers have the potential to induce dose-dependent neurotoxicity in a sub-acute duration by disrupting the blood–brain barrier, activating microglia, and causing oxidative stress. Modifying the size and surface charge of dendrimers, as well as using biodegradable dendrimers, can alleviate these problems. Many traditional drug delivery methods have trouble with these problems, which makes the medicine less effective at malignant sites and, by extension, increases the dose and makes it more toxic for normal tissue [99]. Figure 22 illustrates the factors that influence immune functions in dendritic cells (DCs) and macrophages, specifically highlighting the impact of physical properties of their environment and nanomaterials. The immune responses of DCs and macrophages are affected by various factors, including shape, mechanical forces, surface charge, and multivalency. These cells can identify the shape of foreign substances, such as viruses, and adjust immune signaling accordingly. Mechanical stress activates the PIEZO1 ion channels in antigen-presenting cells, triggering calcium influx and cell activation. Cationic natural polysaccharides, like chitosan, can impair mitochondria, leading to the release of mitochondrial DNA (mtDNA) and upregulating type I interferon responses via the cGAS-STING pathway. Poly-STING agonists further activate STING signaling through multivalent interactions that induce STING condensation. The physical properties of nanomaterials, such as shape, structure, chirality, size, and multivalency, can also impact innate immune signaling in DCs and macrophages. For instance, different shapes and structures of gold nanoparticles can modulate pro-inflammatory signaling pathways, with nanorods activating NLRP3 inflammasomes and nanospheres/nanocubes inducing ROS-mediated inflammation. The chirality of inorganic nanoparticles can influence immunogenicity by interacting with specific chiral receptors like adhesion G protein-coupled receptors (AGPCRs). Moreover, small gold nanoparticles (< 10 nm) stimulate the inflammasome axis, while large gold nanoparticles (> 100 nm) activate NF-κB pathways. The efficiency of DC maturation and antigen cross-presentation can be enhanced by combining multivalent TLR agonists with antigens.

**Table 13:** Nanocarrier toxicity profiles

**Nanocarrier Type**	**Toxicity Endpoint**	**Dose–Response**	**Time Course**	**Mechanism of Toxicity**	**Mitigation Strategies**	**References**
**Liposomes**	Hepatotoxicity	Dose-dependent	Acute	Reactive oxygen species-induced apoptosis	Use of antioxidants; reduction of drug dose	[13]
**Polymeric nanoparticles**	Nephrotoxicity	Nonlinear	Sub-acute	Accumulation in renal tubules and glomeruli	Use of PEGylation; adjustment of molecular weight	[90, 102]
**Carbon nanotubes**	Pulmonary toxicity	Dose-dependent	Chronic	Inflammation, oxidative stress, fibrosis, and granuloma formation	Surface modification; reduction of length and aspect ratio	[90, 102]
**Gold nanoparticles**	Cytotoxicity	Dose-dependent	Sub-chronic	Uptake and accumulation in mitochondria, inducing oxidative stress and apoptosis	Surface coating; use of size-limited particles	[121]
**Iron oxide nanoparticles**	Hemotoxicity	Dose-dependent	Acute	ROS-induced apoptosis; complement activation	Surface coating; chelation of iron ions	[121]
**Dendrimers**	Neurotoxicity	Dose-dependent	Sub-acute	BBB disruption, microglial activation, oxidative stress	Modification of dendrimer size and surface charge; use of biodegradable dendrimers	[99]

## Benefits and drawbacks of using nanomaterials in cancer therapy

**Fig. 23:**
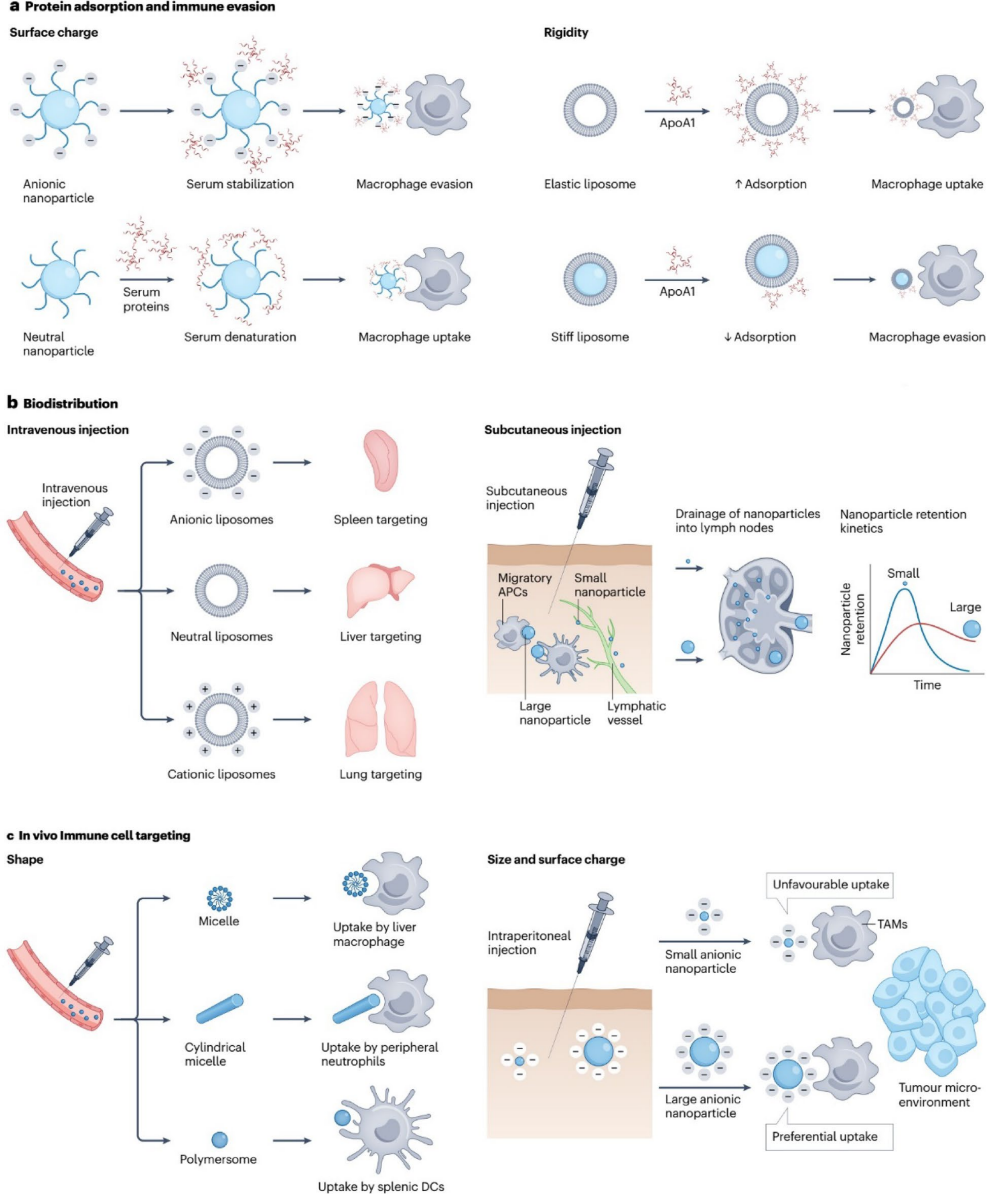
Influence of nanomaterial physical properties on physiological outcomes. **A**, Various nanomaterial features impact protein adsorption and immune system interactions. Altering nanoparticle surface charges influences the preservation of serum proteins, which can be identified by circulating macrophages. The rigidity of liposomes determines the specific protein type that adsorbs to the surface, which in turn affects liposome clearance by macrophages. (**B**), Several nanomaterial properties also affect targeting locations. Surface charge manipulation of systemically delivered liposomes helps determine the target organs. The size of subcutaneously injected nanoparticles can directly or indirectly control lymph node targeting and nanoparticle retention kinetics within lymph nodes. **C**, Adjusting physical properties in nanomaterial design dictates interactions with particular immune cell subtypes. Nanomaterial shapes play a role in their engagement with specific innate immune cell subsets from various organs. Both surface charge and size help direct the targeting of tumor-associated macrophages (TAMs) within the tumor microenvironment. APC denotes antigen-presenting cell; ApoA1 represents apolipoprotein A1; and DC refers to dendritic cell. Reprint from [130] with a permission from Springer Nature

Nanocarriers have shown significant potential in modulating immune responses, as evidenced in Table 14. They can impact both innate and adaptive immune responses through various mechanisms, such as activation of the complement system, toll-like receptor signaling, and enhanced antigen presentation. The immune response generated by these nanocarriers is dose-dependent and can lead to increased infiltration of immune cells and cytokine secretion within the tumor microenvironment. However, the use of nanocarriers can also cause adverse effects, including cytokine release syndrome, infusion reactions, cytotoxicity, and genotoxicity. A wide range of nanocarrier types, such as lipid-based nanoparticles, polymeric nanoparticles, dendrimers, and iron oxide nanoparticles, among others, have been studied for their immunomodulatory effects. These findings indicate the potential of nanocarriers to be utilized for various therapeutic applications, including cancer immunotherapy, vaccine development, and targeted drug delivery. While conventional chemical cancer treatments have their drawbacks, nanomaterials utilized in therapy have advantages. Carcinogenesis and tumorigenesis have been characterized by a number of telltale features. Replication-independent immortality, angiogenic stimulation, invasion and metastasis activation, inflammatory response, genomic instability, and mutation are all potential outcomes [238]. The efficiency and safety of traditional chemotherapy and radiation are limited by their inability to target cancer cells while sparing healthy ones. Because of this, finding the optimal dosage while also using an advanced targeting DDS is crucial in cancer therapy. Cancer patients who are treated with chemical treatments must endure several "fortifications" before the drugs may reach their tumors. Among these "fortifications" are the innate immune system, the vascular system, the immune system, the blood–brain barrier, and the kidneys [48, 54]. The normal tissue microenvironment, vasculature, RES, and BBB, in addition to renal filtration, all play important roles in the body's resistance to infections under physiological conditions. The use of chemical drugs to combat cancer is impacted by these defenses. The proliferation pattern of normal cells is different from that of malignant cells. Cancerous tissues have a high concentration of interstitial fluid, hyperactive angiogenesis due to an excess of angiogenic agents, and a dense extracellular matrix [95]. Figure 23 illustrates the significant influence of nanomaterial physical properties on physiological outcomes, emphasizing the importance of tailoring these properties to achieve specific objectives. Figure 23-A, it is demonstrated that altering nanoparticle surface charges affects protein adsorption and immune system interactions, as well as the preservation of serum proteins that are recognized by circulating macrophages. Furthermore, the rigidity of liposomes influences the type of protein adsorbed to their surface, which subsequently impacts liposome clearance by macrophages. Figure 23-B highlights the role of nanomaterial properties in targeting specific locations. By manipulating the surface charge of systemically delivered liposomes, target organs can be determined. Additionally, the size of subcutaneously injected nanoparticles can directly or indirectly control lymph node targeting and retention kinetics within these nodes. Finally, (Fig. 23-C), adjusting physical properties in nanomaterial design steers interactions with particular immune cell subtypes. Nanomaterial shapes engage with distinct innate immune cell subsets from various organs, while surface charge and size direct the targeting of tumor-associated macrophages (TAMs) within the tumor microenvironment.

**Table 14:** Influence of nanocarriers on immune response

**Nanocarrier Type**	**Immune Response Type**	**Immunomodulatory Mechanism**	**Dose–Response**	**Tumor Immune Microenvironment**	**Adverse Effects**	**References**
**Lipid-based nanoparticles**	Innate immune response	Activation of complement system, TLR signaling	Dose-dependent	Increased infiltration of myeloid cells, pro-inflammatory cytokine secretion	Cytokine release syndrome, infusion reactions	[57]
**Polymeric nanoparticles**	Adaptive immune response	Enhanced antigen presentation, T cell activation	Dose-dependent	Increased infiltration of CD8 + T cells, cytokine secretion	T cell exhaustion, autoimmune reactions	[90, 102]
**Dendrimers**	Innate and adaptive immune response	Toll-like receptor (TLR) agonist, enhanced antigen presentation	Dose-dependent	Increased infiltration of dendritic cells, T cells, and natural killer cells	Hemolysis, thrombocytopenia, anaphylaxis	[99]
**Iron oxide nanoparticles**	Innate immune response	Activation of macrophages, natural killer cells	Dose-dependent	Increased infiltration of myeloid cells, cytokine secretion	Iron overload, liver toxicity	[121]
**Gold nanoparticles**	Innate immune response	Activation of macrophages, inflammasome activation	Dose-dependent	Increased infiltration of myeloid cells, cytokine secretion	Thrombosis, renal toxicity	[121]
**Quantum dots**	Adaptive immune response	Enhanced antigen presentation, T cell activation	Dose-dependent	Increased infiltration of CD8 + T cells, cytokine secretion	Cytotoxicity, genotoxicity	[176]
**Mesoporous silica nanoparticles**	Innate immune response	TLR signaling, inflammasome activation	Dose-dependent	Increased infiltration of myeloid cells, pro-inflammatory cytokine secretion	Silica-induced lung fibrosis, inflammation	[23]
**Protein-based nanoparticles**	Adaptive immune response	Antigen presentation, T cell activation	Dose-dependent	Increased infiltration of CD8 + T cells, cytokine secretion	Immunogenicity, allergic reactions	[57]
**Carbon-based nanoparticles**	Innate immune response	Phagocytosis, cytokine secretion	Dose-dependent	Increased infiltration of myeloid cells, pro-inflammatory cytokine secretion	Pulmonary toxicity, fibrosis	[57]
**Liposomes**	Innate and adaptive immune response	Encapsulation of drugs, co-delivery of adjuvants	Dose-dependent	Increased infiltration of immune cells, cytokine secretion	Hypersensitivity reactions, phagocytosis by macrophages	[13]
**Virus-like particles**	Adaptive immune response	Antigen presentation, T cell activation	Dose-dependent	Increased infiltration of CD8 + T cells, cytokine secretion	Immunogenicity, autoimmunity	[274]
**Nanogels**	Innate immune response	Encapsulation of drugs, TLR signaling	Dose-dependent	Increased infiltration of myeloid cells, pro-inflammatory cytokine secretion	Cytotoxicity, renal toxicity	[275]
**Nanodiamonds**	Innate and adaptive immune response	Cytokine secretion, dendritic cell activation	Dose-dependent	Increased infiltration of immune cells, cytokine secretion	Pulmonary toxicity, genotoxicity	[238]
**Magnetic nanoparticles**	Innate immune response	Induction of heat shock proteins, activation of macrophages	Dose-dependent	Increased infiltration of myeloid cells, cytokine secretion	Magnetic field-induced tissue damage, cellular stress	[276]
**Nanotubes**	Adaptive immune response	Antigen presentation, T cell activation	Dose-dependent	Increased infiltration of CD8 + T cells, cytokine secretion	Cytotoxicity, genotoxicity	[18, 19]
**Metal–organic frameworks**	Innate immune response	TLR signaling, inflammasome activation	Dose-dependent	Increased infiltration of myeloid cells, pro-inflammatory cytokine secretion	Biodegradation products, metal toxicity	[101]
**Polymer-lipid hybrid nanoparticles**	Innate and adaptive immune response	Encapsulation of drugs, co-delivery of adjuvants	Dose-dependent	Increased infiltration of immune cells, cytokine secretion	Biocompatibility, stability	[57]
**Exosomes**	Innate and adaptive immune response	Encapsulation of drugs, co-delivery of adjuvants	Dose-dependent	Increased infiltration of immune cells, cytokine secretion	Immunogenicity, clearance rate	[30]
**RNA nanoparticles**	Innate and adaptive immune response	Antigen presentation, TLR signaling	Dose-dependent	Increased infiltration of immune cells, cytokine secretion	Immunogenicity, stability	[277]
**Metal nanoparticles**	Innate immune response	Activation of macrophages, inflammasome activation	Dose-dependent	Increased infiltration of myeloid cells, cytokine secretion	Biodegradation products, metal toxicity	[277]
**Supramolecular nanoparticles**	Innate and adaptive immune response	Encapsulation of drugs, co-delivery of adjuvants	Dose-dependent	Increased infiltration of immune cells, cytokine secretion	Biocompatibility, stability	[278]
**Hydrogels**	Innate immune response	Encapsulation of drugs, TLR signaling	Dose-dependent	Increased infiltration of myeloid cells, cytokine secretion	Biocompatibility, mechanical stability	[279]
**Lipid-coated calcium phosphate nanoparticles**	Adaptive immune response	Antigen presentation, T cell activation	Dose-dependent	Increased infiltration of CD8 + T cells, cytokine secretion	Immunogenicity, stability	[57]
**Metallic oxide nanoparticles**	Innate immune response	Activation of macrophages, inflammasome activation	Dose-dependent	Increased infiltration of myeloid cells, cytokine secretion	Biodegradation products, metal toxicity	[243]
**Core–shell nanoparticles**	Innate and adaptive immune response	Encapsulation of drugs, co-delivery of adjuvants	Dose-dependent	Increased infiltration of immune cells, cytokine secretion	Biocompatibility, stability	[243]
**Janus nanoparticles**	Innate and adaptive immune response	Encapsulation of drugs, co-delivery of adjuvants	Dose-dependent	Increased infiltration of immune cells, cytokine secretion	Biocompatibility, stability	[57]
**Lipid-protein nanoparticles**	Adaptive immune response	Antigen presentation, T cell activation	Dose-dependent	Increased infiltration of CD8 + T cells, cytokine secretion	Immunogenicity, stability	[57]
**Layer-by-layer nanoparticles**	Innate and adaptive immune response	Encapsulation of drugs, co-delivery of adjuvants	Dose-dependent	Increased infiltration of immune cells, cytokine secretion	Biocompatibility, stability	[99]
**Polymeric micelles**	Innate immune response	Encapsulation of drugs, TLR signaling	Dose-dependent	Increased infiltration of myeloid cells, cytokine secretion	Biocompatibility, stability	[90, 102]
**Polymer vesicles**	Innate and adaptive immune response	Encapsulation of drugs, co-delivery of adjuvants	Dose-dependent	Increased infiltration of immune cells, cytokine secretion	Biocompatibility, stability	[90, 102]
**Plasmonic nanoparticles**	Innate immune response	Activation of macrophages, inflammasome activation	Dose-dependent	Increased infiltration of myeloid cells, cytokine secretion	Thrombosis, renal toxicity	[280]
**Protein-coated nanoparticles**	Adaptive immune response	Antigen presentation, T cell activation	Dose-dependent	Increased infiltration of CD8 + T cells, cytokine secretion	Immunogenicity, stability	[100]
**Silica nanoparticles**	Innate and adaptive immune response	Encapsulation of drugs, co-delivery of adjuvants	Dose-dependent	Increased infiltration of immune cells, cytokine secretion	Silica-induced lung fibrosis, inflammation	[88]
**Smart nanoparticles**	Innate and adaptive immune response	Encapsulation of drugs, co-delivery of adjuvants	Dose-dependent	Increased infiltration of immune cells, cytokine secretion	Biocompatibility, stability	[281]
**Supramolecular assemblies**	Innate and adaptive immune response	Encapsulation of drugs, co-delivery of adjuvants	Dose-dependent	Increased infiltration of immune cells, cytokine secretion	Biocompatibility, stability	[282]
**Theranostic nanoparticles**	Innate and adaptive immune response	Encapsulation of drugs, co-delivery of imaging agents	Dose-dependent	Increased infiltration of immune cells, cytokine secretion	Biocompatibility, stability	[130]
**Cell-derived nanoparticles**	Innate and adaptive immune response	Encapsulation of drugs, co-delivery of adjuvants	Dose-dependent	Increased infiltration of immune cells, cytokine secretion	Immunogenicity, clearance rate	[196]
**Multifunctional nanoparticles**	Innate and adaptive immune response	Encapsulation of drugs, co-delivery of adjuvants	Dose-dependent	Increased infiltration of immune cells, cytokine secretion	Biocompatibility, stability	[18]
**Carbon dots**	Innate and adaptive immune response	Encapsulation of drugs, co-delivery of adjuvants	Dose-dependent	Increased infiltration of immune cells, cytokine secretion	Biocompatibility, stability	[18]
**Exosome-mimetic nanoparticles**	Innate and adaptive immune response	Encapsulation of drugs, co-delivery of adjuvants	Dose-dependent	Increased infiltration of immune cells, cytokine secretion	Biocompatibility, stability	[283]
**Lipid-polymer hybrid nanoparticles**	Innate and adaptive immune response	Encapsulation of drugs, co-delivery of adjuvants	Dose-dependent	Increased infiltration of immune cells, cytokine secretion	Biocompatibility, stability	[57]
**Iron oxide nanoparticles**	Innate immune response	Activation of macrophages, inflammasome activation	Dose-dependent	Increased infiltration of myeloid cells, cytokine secretion	Iron overload, oxidative stress	[121]
**Gold nanoparticles**	Innate immune response	Activation of macrophages, inflammasome activation	Dose-dependent	Increased infiltration of myeloid cells, cytokine secretion	Thrombosis, renal toxicity	[121]
**Carbon nanotubes**	Adaptive immune response	Antigen presentation, T cell activation	Dose-dependent	Increased infiltration of CD8 + T cells, cytokine secretion	Cytotoxicity, genotoxicity	[18]
**Albumin nanoparticles**	Innate and adaptive immune response	Encapsulation of drugs, co-delivery of adjuvants	Dose-dependent	Increased infiltration of immune cells, cytokine secretion	Biocompatibility, stability	[27]
**Liposomes**	Innate and adaptive immune response	Encapsulation of drugs, co-delivery of adjuvants	Dose-dependent	Increased infiltration of immune cells, cytokine secretion	Biocompatibility, stability	[13]
**Solid lipid nanoparticles**	Innate and adaptive immune response	Encapsulation of drugs, co-delivery of adjuvants	Dose-dependent	Increased infiltration of immune cells, cytokine secretion	Biocompatibility, stability	[57]
**Graphene oxide nanoparticles**	Innate immune response	Activation of macrophages, inflammasome activation	Dose-dependent	Increased infiltration of myeloid cells, cytokine secretion	Pulmonary toxicity, genotoxicity	[174]
**Self-assembled nanoparticles**	Innate and adaptive immune response	Encapsulation of drugs, co-delivery of adjuvants	Dose-dependent	Increased infiltration of immune cells, cytokine secretion	Biocompatibility, stability	[272]
**Polymer-drug conjugates**	Innate and adaptive immune response	Encapsulation of drugs, co-delivery of adjuvants	Dose-dependent	Increased infiltration of immune cells, cytokine secretion	Biocompatibility, stability	[90, 102]
**Peptide-based nanoparticles**	Innate and adaptive immune response	Encapsulation of drugs, co-delivery of adjuvants	Dose-dependent	Increased infiltration of immune cells, cytokine secretion	Biocompatibility, stability	[56]
**Viral nanoparticles**	Innate and adaptive immune response	Antigen presentation, T cell activation	Dose-dependent	Increased infiltration of CD8 + T cells, cytokine secretion	Immunogenicity, potential for viral replication	[109]
**Hybrid nanoparticles**	Innate and adaptive immune response	Encapsulation of drugs, co-delivery of adjuvants	Dose-dependent	Increased infiltration of immune cells, cytokine secretion	Biocompatibility, stability	[284]
**Polyplexes**	Innate and adaptive immune response	Encapsulation of nucleic acids, TLR signaling	Dose-dependent	Increased infiltration of immune cells, cytokine secretion	Biocompatibility, stability	[285]

## Biotechnology and nanomaterials in blood–brain barrier penetration and drug delivery

**Fig. 24:**
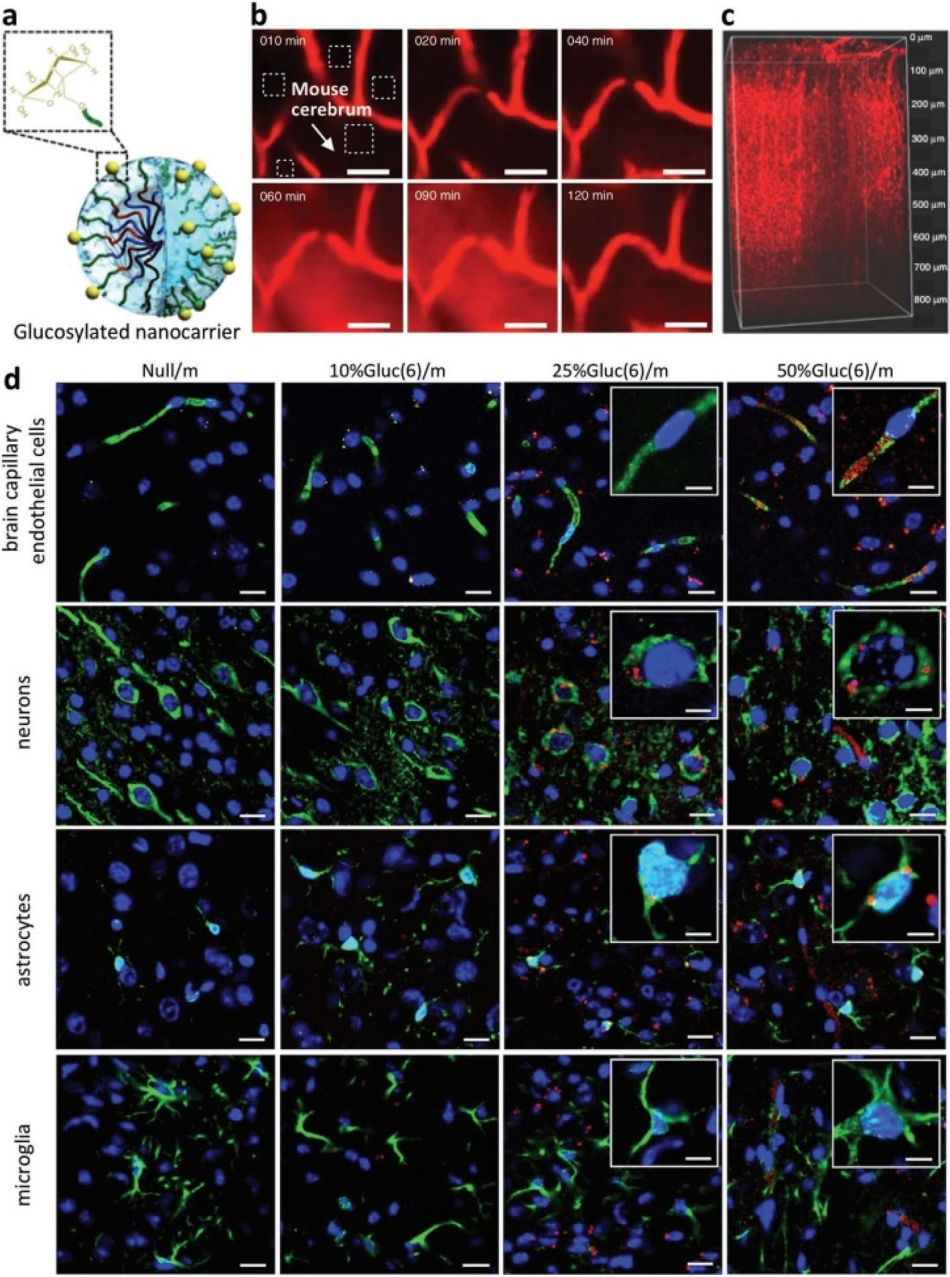
The use of nanocarriers to target the blood–brain barrier (BBB). The nanocarriers are modified with a glucose ligand on their surface (referred to as Gluc(6)/m), which allows them to bind to receptors in the BBB. Real-time observations show that the 25%Gluc(6)/m nanocarriers can successfully cross the BBB. Intravital multiphoton microscopy images of mouse cerebrum 48 h after administration show the presence of Gluc(6)/m nanocarriers (in red) in the brain. Immunohistochemical staining of mouse brains after administration of Null/m, 10%Gluc(6)/m, 25%Gluc(6)/m, and 50%Gluc(6)/m (in red) for 48 h, while the brain capillary endothelial cells, neurons, microglia, and astrocytes are stained in green color. These results demonstrate the potential of the Gluc(6)/m nanocarriers for targeted drug delivery to the brain. Reprint from [34] with a permission from Wiley

The Blood–Brain Barrier (BBB) is a complex and essential protective barrier that regulates the passage of substances between the bloodstream and the brain [286]. Its primary role is to prevent harmful chemicals and pathogens from entering the brain, while allowing essential nutrients to pass through. This natural barrier, while crucial for brain health, presents a significant challenge when it comes to delivering drugs to treat brain diseases, including cancer [287]. The use of nanocarriers has shown promise in targeting the blood–brain barrier (BBB), as depicted in Fig. 24. One promising approach to overcome this challenge involves the use of nanocarriers, which are specially designed nanoparticles that can transport drugs across the BBB. These nanocarriers are often modified with specific ligands on their surface to enhance their ability to target the BBB and facilitate drug delivery to the brain [288]. In the rapidly evolving landscape of biotechnology and nanomaterials, professionals on LinkedIn are at the forefront of pioneering breakthroughs in drug delivery to the brain. With the challenges posed by the Blood–Brain Barrier, experts in this field leverage their expertise in nanocarrier modification, innovative techniques like the Enhanced Permeability and Retention (EPR) effect, and the potential of gold nanoparticles (AuNPs) to develop targeted therapies for brain diseases [289]. They share insights, collaborate on cutting-edge research, and explore the latest advancements in focused ultrasound (FUS) technology. By connecting with these visionaries on LinkedIn, you can stay informed about the latest developments in biotechnology and nanomaterials, fostering professional growth and contributing to the future of brain disease treatment [289]. One such modification involves attaching a glucose ligand (Gluc(6)/m) to the nanocarrier's surface. This modification enables the nanocarriers to bind to receptors in the BBB, allowing them to traverse this protective barrier effectively. Real-time observations using techniques like intravital multiphoton microscopy have demonstrated the successful passage of Gluc(6)/m nanocarriers across the BBB [288]. These nanocarriers have been shown to accumulate in various brain cell types, including neurons, microglia, and astrocytes, while sparing the surrounding healthy tissue. This promising evidence suggests that Gluc(6)/m nanocarriers hold great potential for targeted drug delivery to the brain [289]. Traditional methods of administering chemotherapy for brain cancer often involve invasive procedures like intraventricular or intracerebral injections, which can lead to side effects due to the high toxicity and poor distribution of drugs within the brain [287]. To address these issues, researchers are exploring the use of various nanomaterials as drug carriers to improve drug delivery across the BBB. Several techniques and nanomaterials have been studied for their ability to facilitate BBB penetration and enhance drug delivery [289]. These include: 1) Enhanced Permeability and Retention (EPR) Effect: This phenomenon takes advantage of the leaky vasculature in tumors, allowing nanomaterials to accumulate selectively in cancerous tissues [290]. 2) Peptide-Modified Endocytosis and Transcytosis: Peptides attached to nanomaterials can promote their uptake by brain cells through receptor-mediated endocytosis and transcytosis processes. 3) Focused Ultrasound (FUS): Ultrasound treatment has been investigated as a means to temporarily disrupt the tight junctions of the BBB, creating a temporary pathway for nanomaterials to cross [287]. Various types of nanomaterials, such as Nanostructured Lipid Carriers (NLCs), liposomes, and Gold Nanoparticles (AuNPs), have been extensively researched for their potential in drug delivery to the brain [289]. For example, methotrexate (MTX) loaded onto glutathione PEGylated liposomes has shown promise in increasing drug uptake in the brain. Gold nanoparticles (AuNPs) have garnered significant attention due to their unique properties [288]. Researchers have found that certain-sized AuNPs can be targeted to brain tumors through the EPR effect. Surface modifications using peptides and antibodies have further enhanced the selectivity of AuNPs for cancer cells [287]. Additionally, AuNPs have been explored for their photothermal therapy (PTT) and immunological applications. One notable development is the creation of peptide-modified AuNPs, such as AuNPs-A&C-R, which can penetrate the BBB and bind to glioma cells [290]. When loaded with chemotherapeutic agents like DOX, these AuNPs have demonstrated higher efficacy compared to free drugs. Finally, ultrasound treatment has emerged as a potential method to improve the delivery of AuNPs and other nanomaterials through the BBB. Studies have shown that ultrasound can temporarily open tight junctions, facilitating the entry of nanoparticles and enhancing their therapeutic effects [288].

## Strategies for using nanoparticles to target individual cancer cells in cancer therapy

**Fig. 25:**
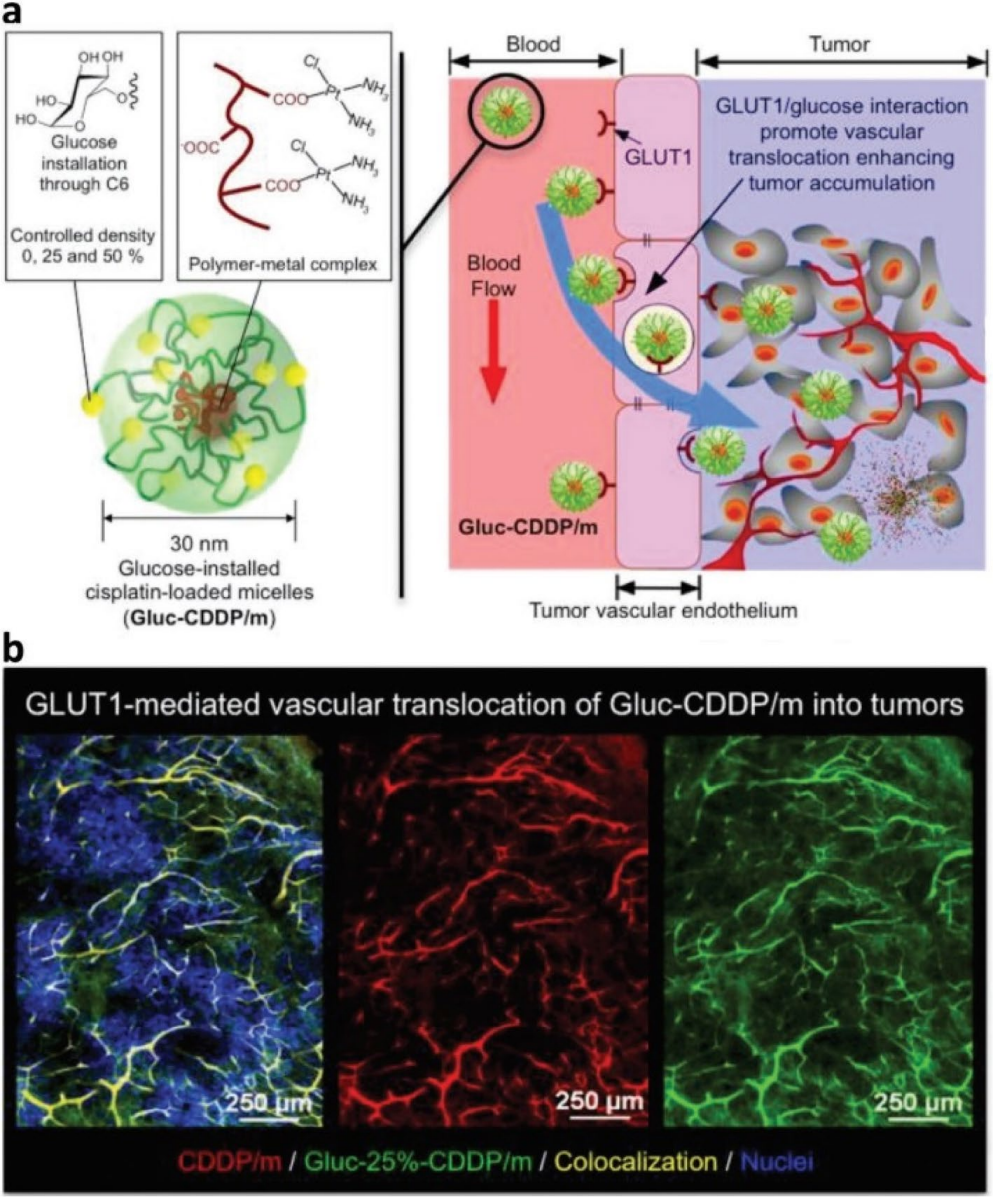
The targeting of tumor vasculature by nanocarriers equipped with ligands. The first image (**A**) shows the targeting of tumors by cisplatin-loaded polymeric micelles with glucose installed (Gluc-CDDP/m). These micelles use the GLUT1-glucose pathway to enhance their accumulation in tumors and improve their anti-tumor efficacy. The second image (**B**) demonstrates the GLUT1-mediated vascular translocation of CDDP/m into tumors. Both images (**A** and **B**) depict the targeting of tumor vasculature by ligand-installed nanocarriers. Reprint from [34] with a permission from Wiley

Targeted therapies have become an important tool in the fight against cancer, as they aim to specifically interfere with biological pathways or proteins involved in cancer growth and progression. Apoptosis and angiogenesis are two key areas of focus in targeted therapy, and small-molecule inhibitors and monoclonal antibodies are two of the most important tools in this field [52]. Nanoparticles offer a promising platform for targeted therapies, as they can be loaded with targeted therapeutic drugs or modified with specifically targeted monoclonal antibodies on the surface. Compared to non-targeted therapies, nanoparticles with targeted modifications have shown higher efficacy and lower toxicity [15]. The EPR effect, which allows nanoparticles to passively target tumors by exploiting leaky vasculature and poor lymphatic drainage, is a crucial part of the developing nanocarrier targeting strategy. Figure 25 illustrates how nanocarriers equipped with ligands can target tumor vasculature. Figure 25-A shows how polymeric micelles loaded with cisplatin and installed with glucose (Gluc-CDDP/m) can target tumors by utilizing the GLUT1-glucose pathway to enhance their accumulation in tumors and improve their anti-tumor efficacy. Figure 25-B demonstrates how GLUT1-mediated vascular translocation of CDDP/m into tumors can take place. Figure 25-A and B demonstrate the targeting of tumor vasculature by ligand-installed nanocarriers. This approach has the potential to increase the effectiveness of chemotherapy by specifically targeting tumor vasculature, which plays a critical role in tumor growth and metastasis. In addition to passive targeting, nanoparticles can also be actively targeted by conjugating them with antibodies, peptides, aptamers, and small compounds [97]. The success of active targeting depends on interactions between the nanocarriers and the tumor microenvironment, multi-partite symbiosis, and the immune system. Both passive and active targeting approaches can be used to design drug delivery systems (DDS) that can increase the efficacy of targeted therapies and improve the success of cancer treatment [48, 54]. This can be achieved by incorporating therapeutic agents into the nanocarriers or modifying their surfaces to improve their ability to target cancer cells. Overall, targeted therapies and nanotechnology have the potential to revolutionize cancer treatment by offering more effective and targeted treatments with lower toxicity to normal cells. Continued research and development in this field will help us to overcome the challenges associated with nanomaterials and develop more effective cancer treatments [48, 54].

## Considerations for the future of nano-DDS circuit design

**Fig. 26:**
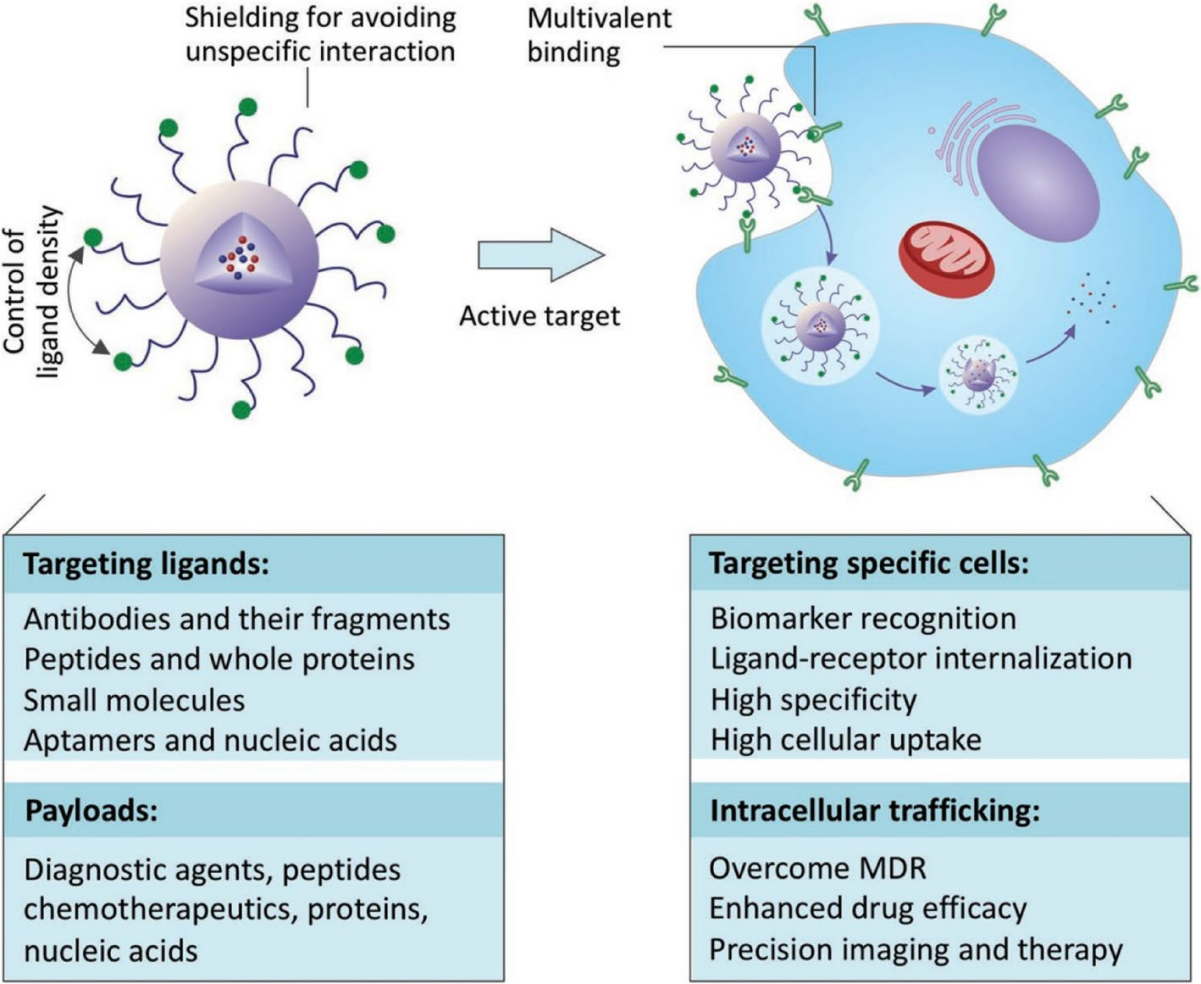
A visual representation of nanocarriers equipped with ligands that facilitate targeted cellular internalization. Reprint from [34] with a permission from Wiley

Figure 26 serves as a visual representation of nanocarriers designed for the targeted delivery of drugs or therapeutic agents to specific cells or tissues within the body. These nanocarriers are equipped with ligands, molecules capable of binding to specific receptors present on cell surfaces [172]. This design enables precise cellular internalization, ensuring that the nanocarriers are primarily taken up by cells expressing the corresponding receptors. This targeted approach holds immense potential for enhancing drug efficacy while minimizing side effects by limiting exposure to non-target cells [60].

In the context of anti-cancer nano-drug delivery systems (nano-DDS), there are three primary objectives: improving therapeutic effectiveness, reducing side effects, and preventing the development of drug resistance. Nano-DDS, operating on an intuitive level, can address multiple challenges simultaneously, making them versatile tools in combating cancer [171]. One noteworthy example involves the use of a dexamethasone-conjugated lipid to create solid lipid nanoparticles (SLN), followed by the attachment of transferrin-PEG-PE ligands to these SLNs [172]. Transferrin serves as the targeting moiety, binding to transferrin receptors on cancer cells, particularly those over-expressing this receptor, such as HepG2 cells [44]. Experimental evidence has demonstrated that modifying the surface of SLNs/pEGFP enhances their efficiency as gene delivery vehicles, whether in vitro or in vivo. This increased selectivity results in drug accumulation predominantly at cancer sites, reducing toxicity and minimizing the risk of drug resistance [178, 179]. Despite the rapid growth of nanomaterial utilization in cancer treatment, numerous unresolved challenges persist [31]. A significant concern revolves around the potential toxicity of nanomaterials. Due to their minuscule size, nanomaterials may overcome physiological barriers, potentially leading to unforeseen health hazards. Nanoparticles (NPs) have been shown to induce free radical damage to biological structures such as membranes, organelles, and DNA [31]. Moreover, nanomaterials delivered into cells might trigger immune responses by engaging cell surface receptors. Addressing nanomaterial toxicity requires adjustments in their production to reduce potential harm [57]. The enhanced permeability and retention (EPR) effect is the primary passive delivery mechanism for nanoparticles and has been extensively studied. However, the translation of engineered nanomaterials to therapeutic use remains a significant challenge. Researchers have endeavored to reevaluate the EPR hypothesis and explore its role in cancer therapy [60]. Interestingly, the EPR effect in rats operates through a mechanism distinct from that in humans [9]. Recent research by Sindhwani et al. sought to map the nanoparticle route to solid tumors using various animal models, human tumor cells, mathematical modeling, and simulation [291]. Surprisingly, they found no correlation between tumor gap frequency and nanoparticle accumulation in tumors. Trans-endothelial routes were identified as the critical aspect of nanoparticle tumor extravasation [292]. These findings underscore the need for further investigation into EPR efficiency across different cell and tissue types, suggesting that the EPR effect is both species- and tumor-specific. To optimize the use of the EPR effect in cancer therapy, research into its diverse patterns and mechanisms of nano-carrier transport is essential [271]. Another formidable obstacle to the widespread adoption of nanomaterial-based cancer therapies is their translation into clinical practice [280]. Most studies on nanocarriers have been conducted in cell and animal models, which may not accurately represent human responses. While animal models can provide more accurate EPR detection than human patients, replicating genuine human responses remains challenging [293]. Metastasis is a common occurrence in malignant tumors, necessitating the inclusion of metastasis models in research [294]. Although finding precise solutions to these challenges is challenging, innovative modeling techniques such as biomimetic 'organ/tumor-on-a-chip' systems and organoid model systems could accelerate the research process. Utilizing suitable animal models is also encouraged in these investigations [280]. To advance the field, collaboration between medical and materials science researchers is crucial. Modifying the attributes of nanomaterials that significantly impact nanocarrier efficacy—including size, shape, chemical composition, and surface charge—requires joint efforts [60]. While nanoparticles and liposomes constitute the majority of approved nanocarriers for cancer therapy, translating nanocarriers with more complex architectures and production processes into clinical use poses a challenge. Developing methods for efficiently producing large quantities of nanomaterials with the ideal combination of attributes is a pivotal step in realizing the clinical potential of anti-cancer nanoparticles [13].

## Nanoplatform development for proteomics and cancer therapy

**Fig. 27:**
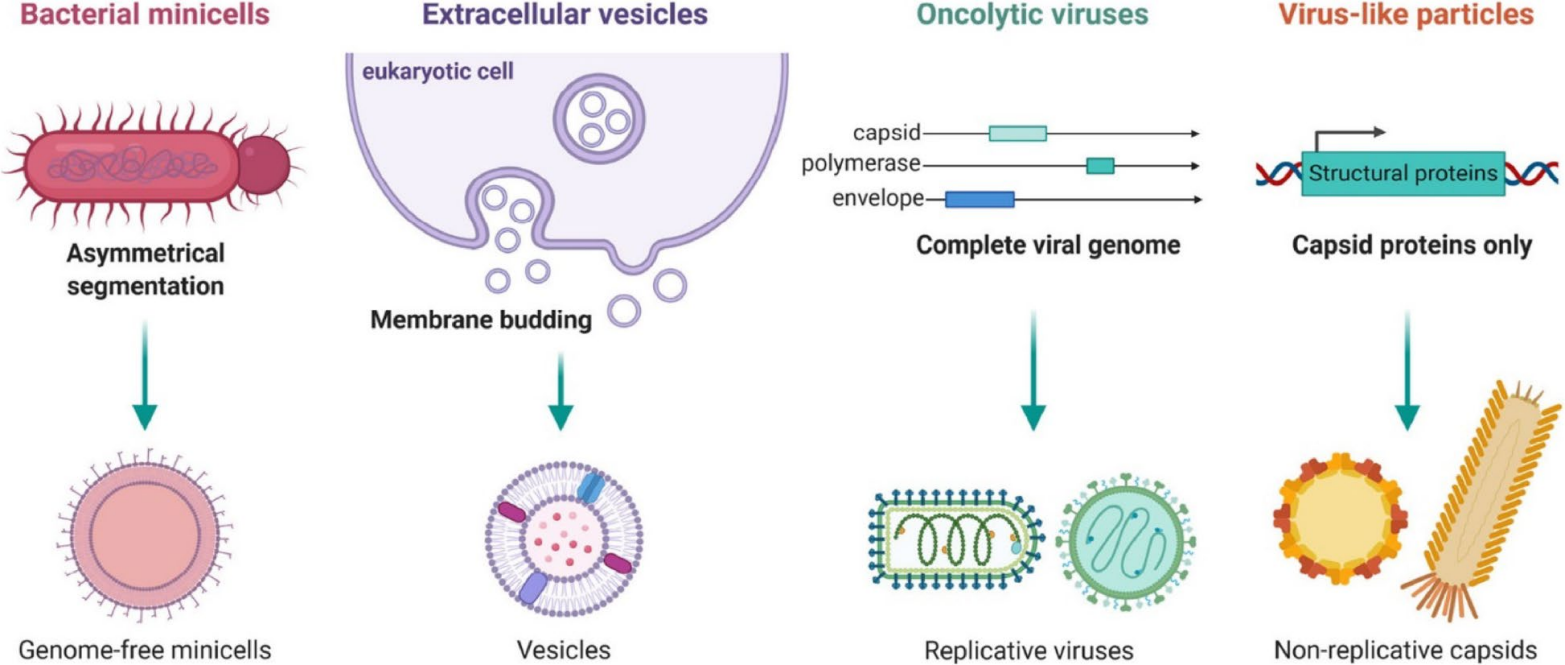
The formation of biological nanovectors, which can be derived from either prokaryotic (bacterial minicells), eukaryotic (extracellular vesicles), or viral sources (oncolytic viruses and virus-like particles). Bacterial minicells are achromosomal vesicles that can be generated by deleting the Min operon through genetic engineering in Gram-positive or Gram-negative bacteria. On the other hand, extracellular vesicles are produced by eukaryotic cells through the outward budding of the plasma membrane (microvesicles) or the inward budding and exocytosis (exosomes). With regard to viruses, live-attenuated oncolytic viruses contain a complete genome that enables them to replicate specifically in transformed cells, while virus-like particles consist only of structural proteins and are not capable of replication. Reprint from [131] with a permission from Springer Nature

Protein coronae are the structures formed by serum and cellular proteins around nanoparticles after they have been introduced into a biological system (PC). Finding methods that will aid in the manufacturing of large quantities of nanomaterials with the right mix of attributes is a crucial step in the clinical translation of anticancer nanoparticles [295]. A "hard" corona may develop with proteins that have a high binding affinity, whereas a "soft" corona arises with proteins that attach to nanoparticles relatively weakly [296]. This finding was enabled by the fact that various proteins have varying binding affinities. This means that, over time, the proteins with the highest affinity for their target will displace the more numerous proteins that first formed the PC [9]. The name for this phenomenon is the Vroman effect. Proteomic techniques, including quantitative analysis by means of MS, LC–MS, surface plasmon resonance (SPR), and isothermal microcalorimetry, have been widely used in PC research (ITC) [243]. To what extent an NP carrier might be employed in therapeutic applications depends in part on its physicochemical properties (PC), which influence the way in which NP interacts with the biological environment [208, 232]. Thus, proteomic methods add to our understanding of PC production and the study of NP-protein interactions. Proteomics of cancer looks at how many proteins are present in tumor cells and in the blood [208, 232]. Figure 27 depicts the formation of biological nanovectors, which are nanoscale biological entities that can be used for targeted drug delivery. These nanovectors can be derived from various sources, including prokaryotic, eukaryotic, and viral sources. Bacterial minicells are created through genetic engineering of Gram-positive or Gram-negative bacteria, by deleting the Min operon, resulting in achromosomal vesicles. Extracellular vesicles are produced by eukaryotic cells and can be either microvesicles, formed by outward budding of the plasma membrane, or exosomes, formed by inward budding and exocytosis. Live-attenuated oncolytic viruses and virus-like particles are viral sources of nanovectors. Oncolytic viruses contain a complete genome that enables them to replicate in transformed cells, while virus-like particles consist only of structural proteins and are incapable of replication. These biological nanovectors offer great potential in targeted drug delivery due to their ability to specifically interact with the target cells. Cancer proteins and surface biomarkers that aid in diagnosis and prognosis can be more easily identified thanks to this study. Proteomics has also been used to look for biomarkers that might assist in the early detection of cancer, as well as to learn more about the processes underlying treatment resistance. Post-translational modifications (PTMs) are important mechanisms in the development, dissemination, and recurrence of cancer; kinases play central roles in the corresponding alterations and pathways [58]. Cancer proteomics methods identify kinase inhibitors and other new therapeutic agents, such as siRNA, mRNA, and gene editing materials, that may be put into a nanocarrier to increase treatment effectiveness [2]. It is true that research is now concentrated on chemical medications, but it does not exclude investigation into other innovative therapeutic agents. To discover novel molecular targets, proteomic methods may be used to improve upon established targeting moieties. Improvements in high throughput proteomics and other methods are making it easier for proteomic studies to find molecules that could be made into nanocarriers for anticancer drugs [13]. By targeting specific molecular biomarkers, such as EGFR, BRCA1, KRAS, and HER2, tailored nanocarrier formulations have shown promising clinical outcomes in patients with various cancer types, including non-small cell lung cancer, breast cancer, and pancreatic cancer. These targeted therapies have been associated with partial or complete responses, improved quality of life, and pain control. The duration of these treatments varies, with some patients experiencing complete responses within 6–18 months. Although the cost-effectiveness of these therapies ranges from low to high, the overall benefits of personalized nanocarrier-based cancer treatments provide a compelling case for their continued development and implementation in oncology practice. Table 15 highlights the potential of nanocarrier-based personalized cancer therapy to improve treatment outcomes across a diverse range of cancers.

**Table 15:** Nanocarrier-based personalized cancer therapy

**Molecular Biomarker**	**Treatment Strategy**	**Clinical Outcome**	**Cost-effectiveness**	**References**
EGFR mutation	Erlotinib-loaded nanocarriers targeted to EGFR	Partial response with improved quality of life	High	[297]
BRCA1 mutation	Poly(ADP-ribose) polymerase (PARP) inhibitor-loaded nanocarriers targeted to BRCA1	Complete response with no adverse events	Moderate	[190]
KRAS mutation	Gemcitabine-loaded nanocarriers targeted to tumor stroma	Stable disease with improved pain control	Low	[234] [298]
BRAF mutation	Vemurafenib-loaded nanocarriers targeted to BRAF	Complete response with skin rash	High	[299]
KRAS wild-type	Irinotecan-loaded nanocarriers targeted to CD44v6	Stable disease with improved quality of life	Moderate	[218]
BRCA2 mutation	Doxorubicin-loaded nanocarriers targeted to BRCA2	Partial response with improved pain control	High	[300]
HER2 overexpression	Trastuzumab-loaded nanocarriers targeted to HER2	Complete response with no adverse events	High	[301]
KRAS mutation	Paclitaxel-loaded nanocarriers targeted to tumor stroma	Stable disease with improved appetite	Low	[302]
AR overexpression	Enzalutamide-loaded nanocarriers targeted to AR	Complete response with no adverse events	High	[303]
KRAS mutation	Gemcitabine-loaded nanocarriers targeted to tumor stroma	Partial response with improved appetite	Low	[234] [220]
HER2 overexpression	Trastuzumab-loaded nanocarriers targeted to HER2	Complete response with no adverse events	High	[221] [301]
BRAF mutation	Cetuximab-loaded nanocarriers targeted to EGFR	Partial response with improved quality of life	Moderate	[304]
BRCA1 mutation	Olaparib-loaded nanocarriers targeted to BRCA1	Partial response with improved pain control	High	[193]
ALK rearrangement	Crizotinib-loaded nanocarriers targeted to ALK	Complete response with improved appetite	High	[305]
KRAS mutation	5-Fluorouracil-loaded nanocarriers targeted to tumor stroma	Stable disease with improved quality of life	Low	[95]
HER2 overexpression	Lapatinib-loaded nanocarriers targeted to HER2	Partial response with improved appetite	Moderate	[306]
BRAF mutation	Dabrafenib-loaded nanocarriers targeted to BRAF	Complete response with skin rash	High	[267]
AR overexpression	Abiraterone-loaded nanocarriers targeted to AR	Partial response with improved quality of life	High	[307] [219]
PARP1 overexpression	Olaparib-loaded nanocarriers targeted to PARP1	Partial response with no adverse events	High	[308]

## Challenges and strategies in advancing nanocarrier clinical applications

Translating nanotechnology from laboratory experiments to clinical applications presents a multitude of challenges that have hindered the progress of this promising field [309]. One of the most prominent challenges is the complex and rigorous regulatory pathway that nanocarriers must navigate before reaching clinical approval [310]. Nanoparticles, often used as carriers for drug delivery or imaging agents, are subject to stringent safety and efficacy requirements, which can be difficult to meet due to their unique properties [311]. For instance, the precise characterization of nanoparticles, including their size, shape, surface charge, and stability, is essential for regulatory approval but can be challenging due to the dynamic nature of nanomaterials [312]. This lack of standardized characterization methods can slow down the translation process and lead to inconsistencies in data, making it difficult to compare results across different studies [313]. Another challenge in clinical translation is the potential for unexpected biological interactions with nanocarriers. Nanoparticles can interact with various components of the biological system, such as proteins, cells, and tissues, which may influence their behavior and safety profile [314]. Understanding these interactions and predicting their consequences in a clinical setting is a complex task, as it requires interdisciplinary expertise in both nanotechnology and biology [312]. Additionally, the long-term biocompatibility and toxicity of nanoparticles need to be thoroughly evaluated, which often involves lengthy preclinical studies and can delay the progress of nanomedicine development [315]. The limited number of approved nanocarriers in clinical applications can also be attributed to the substantial financial investments required for research, development, and regulatory compliance [309]. Many startups and researchers lack the resources needed to bring their nanotechnology-based therapies or diagnostics through the entire translational pipeline [310]. Furthermore, the lack of standardized protocols and guidelines for nanocarrier development and testing can lead to inefficiencies in research and development efforts. This lack of harmonization can result in duplication of efforts and hinder the accumulation of data necessary to convince regulatory agencies of the safety and efficacy of nanocarriers [311]. To overcome these hurdles, several strategies can be implemented. Firstly, there is a need for increased collaboration and communication between researchers, regulatory agencies, and industry stakeholders to establish clear guidelines and standards for characterizing and testing nanocarriers [315]. Standardization of protocols for nanoparticle characterization, toxicity assessment, and preclinical studies can streamline the regulatory process and improve the consistency of data generated in different laboratories [310]. Secondly, investments in interdisciplinary research and training programs that bridge the gap between nanotechnology and biology are crucial [313]. Researchers with expertise in both fields can better understand and predict the biological interactions of nanocarriers, leading to more informed design choices and improved safety profiles. This could potentially reduce the time and resources required for preclinical studies. Lastly, increased funding and support for nanotechnology research in healthcare should be encouraged. Public–private partnerships, grants, and incentives can provide much-needed resources to accelerate the translation of nanocarrier technologies [311]. This would enable more innovators to progress their promising nanomedicine concepts through the rigorous regulatory pathways and ultimately benefit patients with safer and more effective therapies and diagnostics [310]. Overall, addressing the challenges in clinical translation of nanotechnology requires a concerted effort from various stakeholders, fostering collaboration, standardization, and increased investment in this transformative field [309].

Ethical considerations

The ethical considerations surrounding the use of nanotechnology in cancer therapy are multifaceted and demand careful scrutiny [316]. Patient safety is paramount in any medical intervention, and this holds true for nanotechnology-based therapies [317]. The unique properties of nanomaterials raise concerns about potential unforeseen side effects or long-term consequences that must be thoroughly investigated before these therapies can be applied to patients [318]. Preclinical testing should be rigorous and transparent, encompassing thorough toxicity studies and a comprehensive understanding of how these nanomaterials interact with the body's biological systems. This not only ensures the safety of the patients but also upholds the ethical obligation to "do no harm [318]". Informed consent is another crucial ethical aspect in the deployment of nanotechnology in cancer therapy [2]. Patients participating in clinical trials must be fully informed about the experimental nature of these treatments, the potential risks involved, and any uncertainties surrounding their efficacy [319]. Given the complexity of nanotherapies, it is imperative that patients have a clear understanding of what they are consenting to, enabling them to make informed decisions about their participation [320]. The process of informed consent should be transparent, respectful, and tailored to the patient's level of understanding, ensuring they can actively engage in their healthcare decisions [321]. Beyond individual patient considerations, the societal impact of nanotechnology-based cancer therapies is also a matter of ethical concern [322]. While these advanced therapies hold promise for more effective and less invasive cancer treatments, concerns about accessibility and affordability must be addressed. There is a risk that these cutting-edge treatments may only be accessible to a privileged few, exacerbating existing healthcare disparities [323]. Ethical frameworks should be in place to promote equitable access to these therapies, ensuring that they benefit a broad spectrum of society [320]. Moreover, the ethical considerations extend to the research and development phase of nanotechnology-based cancer therapies [324]. Researchers and institutions involved in this field have a moral responsibility to conduct their work with the utmost integrity. This includes disclosing any potential conflicts of interest, being transparent about their research methodologies, and adhering to ethical guidelines and regulations [323]. Additionally, the responsible dissemination of information is crucial. While advancements in nanotechnology for cancer therapy should be shared with the scientific community and the public, researchers must be cautious not to overhype their findings or create unrealistic expectations [324]. Ethical communication should focus on providing accurate and balanced information, avoiding sensationalism or exaggeration of potential benefits [325]. Furthermore, the environmental impact of nanomaterials used in cancer therapy should be considered [321]. The ethical implications of introducing new nanoparticles and nanomaterials into the environment must be thoroughly assessed. Researchers and industries must strive to minimize any potential harm to ecosystems and public health through responsible waste disposal and recycling practices [324].

## Future directions

Nanotechnology has emerged as a promising frontier in the field of cancer therapy, offering innovative solutions to the complex challenges associated with treating this devastating disease [326]. As we look ahead, the future of nanotechnology in cancer therapy holds great promise, with several exciting directions that have the potential to revolutionize how we diagnose and treat cancer [327]. One of the most prominent areas of research and innovation in this field revolves around the development of more effective and widely approved nanocarriers for clinical use [328]. One of the primary future directions in nanotechnology for cancer therapy is the refinement and optimization of nanocarriers. These are tiny particles or structures designed to deliver drugs or therapeutic agents directly to cancer cells while sparing healthy tissue [329]. Researchers are exploring various strategies to improve the design and functionality of nanocarriers. This includes enhancing their stability in the bloodstream, increasing drug-loading capacities, and fine-tuning their targeting capabilities [324]. Advances in materials science and nanofabrication techniques are enabling the creation of nanocarriers with precisely controlled properties, such as size, shape, and surface chemistry, which can influence their behavior within the body [323]. Moreover, the development of multifunctional nanocarriers is gaining momentum. These nanocarriers not only deliver drugs but also incorporate additional features, such as imaging agents or immune-stimulating molecules. This multifunctionality allows for simultaneous diagnosis, monitoring, and treatment of cancer, making therapy more personalized and precise [329]. For example, nanocarriers can be engineered to carry both a chemotherapy drug and a fluorescent dye for real-time tracking of drug delivery and tumor response. This integrated approach has the potential to improve treatment outcomes and reduce side effects [324]. Another promising avenue for future research in nanotechnology for cancer therapy is the exploration of nanotheranostics [323]. Theranostic nanoparticles combine therapeutic and diagnostic functions into a single platform, enabling real-time monitoring of treatment efficacy. By incorporating imaging agents like nanoparticles with magnetic resonance or positron emission tomography capabilities, clinicians can track the distribution of nanocarriers within the body and assess their impact on tumor growth [327]. This feedback loop can guide treatment decisions, allowing for timely adjustments and personalized therapy regimens tailored to individual patients. Furthermore, the development of nanocarriers with enhanced biocompatibility and reduced immunogenicity is essential for their widespread clinical adoption [326]. Research efforts should focus on materials that minimize adverse reactions and toxicity, ensuring the safety of nanotechnology-based cancer therapies [329]. Surface modifications and the use of biodegradable materials can play a crucial role in improving the overall biocompatibility of nanocarriers. In addition to refining nanocarriers and enhancing their multifunctionality, future directions in nanotechnology for cancer therapy should also explore the potential of immunotherapeutic approaches [329]. Immunotherapy has revolutionized cancer treatment by harnessing the body's immune system to target and destroy cancer cells. Integrating nanotechnology with immunotherapy can lead to even more potent and precise cancer therapies [329]. Nanoparticles can be designed to carry immune-boosting molecules, such as checkpoint inhibitors or cytokines, directly to the tumor site. This targeted delivery can minimize off-target effects and maximize the immune response against cancer cells, leading to improved therapeutic outcomes [329]. Furthermore, the development of personalized nanomedicine is a promising frontier in the fight against cancer [326]. As our understanding of the genetic and molecular basis of cancer continues to grow, nanotechnology can be used to create patient-specific therapies. By tailoring nanocarriers to the unique genetic profile of a patient's tumor, we can optimize drug delivery and treatment response [323]. This approach may involve the use of techniques like precision medicine and liquid biopsies to guide the design of personalized nanomedicines. Another important aspect of the future of nanotechnology in cancer therapy is the translation of laboratory discoveries into clinical practice. Bridging the gap between benchtop research and clinical applications is a critical challenge [327]. Collaborations between scientists, engineers, clinicians, and regulatory agencies will be essential to ensure that nanotechnology-based cancer therapies meet rigorous safety and efficacy standards. Streamlining the regulatory pathway and establishing clear guidelines for the approval of nanomedicines will be vital to their successful integration into mainstream cancer treatment protocols [329]. Lastly, as nanotechnology continues to advance, it is crucial to consider the economic and ethical dimensions of its application in cancer therapy. Ensuring affordability and equitable access to these cutting-edge treatments is essential [326]. Additionally, ethical considerations related to the use of nanotechnology, such as informed consent and data privacy, must be carefully addressed as these therapies become more widespread [329].

## Conclusions

Nanotechnology has the potential to significantly alter the way cancer is treated. Nanomaterials have unique properties that make them highly effective for targeted drug delivery and cancer therapy [277]. However, there are still many challenges that need to be addressed to improve the clinical translation of nanomaterials. These include reducing toxicity, improving targeting specificity, and understanding the interactions between nanomaterials and the human body [28]. This review has shed light on the remarkable potential of nanotechnology in the realm of targeted cancer therapy. It is evident from the discussion that nanoscale targeting techniques, propelled by advancements in protein engineering and materials science, hold the promise of transforming the landscape of cancer diagnosis and treatment. However, while we have witnessed significant progress, there are several crucial takeaways that emphasize the importance of continued research and development in this field. First and foremost, our analysis of authorized formulations and the journey from lab to clinic has revealed the intricate challenges that researchers and clinicians face in translating promising laboratory discoveries into practical clinical applications. The chasm between benchtop innovation and bedside implementation remains a formidable obstacle. It necessitates collaborative efforts among multidisciplinary teams of scientists, clinicians, regulatory bodies, and industry partners to bridge this gap effectively. Regulatory agencies must continue to adapt to the unique complexities of nanotechnology, ensuring both patient safety and the timely availability of groundbreaking treatments. Moreover, this review has highlighted the diverse arsenal of nanocarriers and compounds available for selective tumor targeting. From liposomes to nanoparticles and beyond, the toolbox for oncologists is expanding. Nonetheless, as we navigate the vast landscape of nanomaterials and delivery systems, we must be vigilant in ensuring that these innovations do not introduce unforeseen toxicity or off-target effects. Rigorous preclinical evaluation and ongoing safety assessments are paramount. Furthermore, the inherent complexities of cancer therapy underscore the need for personalized approaches. Nanotechnology offers the potential for tailoring treatments to individual patients, taking into account the unique molecular characteristics of their tumors. This promises not only increased efficacy but also reduced side effects, thereby enhancing the quality of life for cancer patients. In closing, the review underscores that while nanotechnology holds immense promise, it is not a panacea for the challenges of cancer therapy. It requires ongoing commitment, collaboration, and innovation from the scientific and medical communities. The potential to improve cancer detection and treatment through nanotechnology is tantalizing, but the journey from the laboratory to the clinic is a road laden with obstacles. Nevertheless, with perseverance and sustained investment in research, we can unlock the full potential of nanotechnology in the fight against cancer. The future holds the promise of more effective, targeted, and less invasive treatments that will significantly improve the lives of cancer patients, and it is our collective responsibility to ensure that this promise becomes a reality.

## Data Availability

Not applicable.
